# Dual Opioid–Neuropeptide FF Small Molecule Ligands Demonstrate Analgesia with Reduced Tolerance Liabilities

**DOI:** 10.3390/molecules30132851

**Published:** 2025-07-03

**Authors:** Marco Mottinelli, V. Blair Journigan, Samuel Obeng, Victoria L. C. Pallares, Christophe Mѐsangeau, Coco N. Kapanda, Stephen J. Cutler, Janet A. Lambert, Shainnel O. Eans, Michelle L. Ganno, Wanhui Sheng, Tamara King, Abhisheak Sharma, Catherine Mollereau, Bonnie A. Avery, Jay P. McLaughlin, Christopher R. McCurdy

**Affiliations:** 1 Department of BioMolecular Sciences, School of Pharmacy, University of Mississippi, University, MS 38677, USA; m.mottinelli@northeastern.edu (M.M.); sjcutler@cop.sc.edu (S.J.C.);; 2 Department of Medicinal Chemistry, College of Pharmacy, University of Florida, 1345 Center Drive, Gainesville, FL 32610, USA; 3 Department of Cellular and Systems Pharmacology, College of Pharmacy, University of Florida, 1345 Center Drive, Gainesville, FL 32610, USAshaieans@ufl.edu (S.O.E.);; 4 Torrey Pines Institute for Molecular Studies, Port St. Lucie, FL 34987, USA; 5 Department of Pharmaceutics and Drug Delivery, School of Pharmacy, University of Mississippi, University, MS 38677, USAasharma1@ufl.edu (A.S.);; 6 Centre de Recherches sur la Cognition Animale (CRCA-CBI), Université de Toulouse, 169, Avenue Marianne Grunberg-Manago, 31062 Toulouse, France; catherine.mollereau@utoulouse.fr

**Keywords:** opioid, neuropeptide FF, designed multiple ligands, nociception, tolerance

## Abstract

Neuropeptide FF (NPFF) receptor antagonists prevent morphine-mediated antinociceptive tolerance, and compounds with dual mu opioid receptor (MOR) agonist and NPFF antagonist activity produce antinociception without tolerance. Compounds synthesized showed affinities in radioligand competition binding assays in the nM and µM range at the opioid and NPFF receptors, respectively, and displayed substitution-dependent functional profiles in the [^35^S]GTPγS functional assay. From six compounds screened in vivo for antinociception and ability to prevent NPFF-induced hyperalgesia in mouse warm water tail withdrawal tests, compound **22b** produced dose-dependent MOR-mediated antinociception with an ED_50_ value (and 95% confidence interval) of 6.88 (4.71–9.47) nmol, i.c.v., and also prevented NPFF-induced hyperalgesia. Meanwhile, **22b** did not demonstrate the respiratory depression, hyperlocomotion, or impaired intestinal transit of morphine. Moreover, repeated treatment with **22b** produced a 1.6-fold rightward shift in antinociceptive dose response, significantly less acute antinociceptive tolerance than morphine. Evaluated for microsomal stability in vitro and in vivo pharmacokinetic profile, **22b** showed suitable microsomal stability paired in vivo with a large apparent volume of distribution and a clearance smaller than the hepatic flow in rats, suggesting no extra-hepatic metabolism. In conclusion, the present study confirms that dual-action opioid–NPFF ligands may offer therapeutic promise as analgesics with fewer liabilities of use.

## 1. Introduction

Mu opioid receptor (MOR) agonists are a commonly prescribed class of medication for the treatment of moderate to severe pain in the United States [1], with more than 142 million prescriptions for opioid analgesics dispensed in 2020 [2]. However, the use of opioids is hampered by several adverse effects, including physiological analgesic tolerance, respiratory depression, and opioid-induced hyperalgesia (OIH), as well as the more complex phenomena of dependence and addiction [3,4,5]. Opioid overdose caused 54,743 deaths in the U.S. in 2024 [6], underscoring the need for new potent analgesics with reduced liabilities of use.

Numerous mechanisms are thought to contribute to opioid analgesic tolerance, such as opioid receptor desensitization mediated by phosphorylation from intracellular kinases and subsequent arrestin recruitment [7,8]. Additional evidence demonstrates the induction of tolerance by opioid system activation of endogenous anti-opioid systems as a homeostatic adaptation, producing paradoxical but clinically relevant opioid-induced hyperalgesia (OIH). The development of OIH reduces the analgesic effect of an opioid, requiring greater dosages to achieve the same analgesic response (tolerance), while persistent adverse effects reduce the safety index [9,10,11,12,13,14].

Signaling of the neuropeptide FF (NPFF) system may mediate OIH [15,16]. The NPFF system is comprised of two receptor subtypes, NPFF1-R and NPFF2-R, and four endogenous ligands belonging to the RFamide family (NPFF, NPSF, NPAF, and NPVF) [16]. The NPFF system was characterized as an anti-opioid, pro-nociceptive system based on the in vivo activity of the non-selective dipeptide antagonist RF9 [17]. Co-administration of RF9 with the MOR agonist heroin maintains analgesic activity while preventing OIH and associated tolerance via selective antagonism of NPFF1-R [18,19] and NPFF2-R [17]. Similarly, co-administration of the NPFF1-selective dipeptide antagonist 1 with the MOR agonist fentanyl prolongs fentanyl-induced analgesia and decreases its associated hyperalgesia [20]. Notably, while these studies defined an anti-opioid role for the NPFF1-R subtype, other studies report an indirect antinociceptive function for NPFF2-R [21], although a mechanism remains uncertain. Despite these alternatives, most of the limited evidence available and the non-selective binding affinity of RF9 suggest that both NPFF1-R and NPFF2-R subtypes mediate anti-opioid activity.

Further studies with the dipeptide RF9 and recent analogs solidify a role for NPFF antagonists as novel pharmacotherapies to improve the analgesic efficacy of opioids [22,23,24]. Pretreatment with RF9 before fentanyl or morphine abolishes acute and long-term hyperalgesia, as well as tolerance, in Swiss and C57BL/6N mice and decreases naltrexone-precipitated withdrawal symptoms [22]. Interestingly, RF9 prevents the development but not the expression of morphine tolerance; when RF9 is given after morphine tolerance is established, the reduced tail flick latency indicates that a single dose of an NPFF antagonist alone cannot reverse pre-existing tolerance [22]. This evidence highlights the advantages of having an adjunct NPFF-R antagonist present at the initiation of opioid therapy, providing the premise for our approach of targeting a combined pharmacologically multifunctional activity in a single ligand.

**Figure 1:**
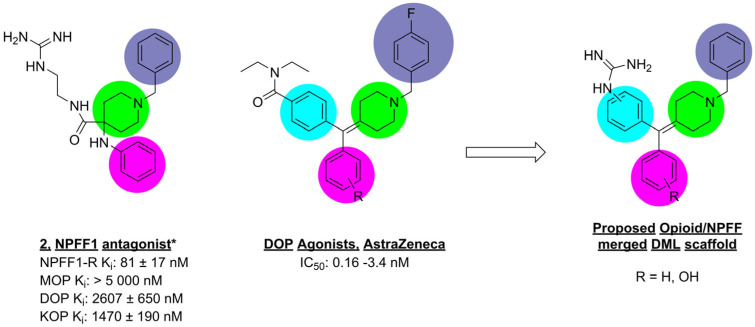
Merged Designed Multiple Ligand (DML) approach for nonpeptide small molecule opioid agonist/NPFF antagonist ligands. In green, purple, and magenta are highlighted the envisaged common pharmacophoric properties * [29].

Multiple receptors with desired activities can be targeted by specifically designed molecules, an approach termed “designed multiple ligand” (DML). In general, DMLs offer attractive features over a drug cocktail or multicomponent drug, such as increased patient compliance, more controllable pharmacokinetic (PK) and pharmacodynamic profiles, and a lower risk of drug–drug interactions [25]. Multiple articles have reported the search for dual opioid/NPFF ligands, but these generally involve the use of small peptides [26,27,28]. To our knowledge, no small molecule dual ligands simultaneously targeting opioid and NPFF1 or NPFF2 receptors have been reported. We previously reported a guanidino–piperidine series of NPFF1 and NPFF2 receptor ligands, exemplified by NPFF1-R preferential antagonist 2 (Figure 1), which prevents NPFF-induced hyperalgesia in the mouse 48 °C warm water tail withdrawal test [29]. Specifically, an opioid scaffold containing a 1,4-diaryl substituted piperidine core was desired to maintain affinity at NPFF-Rs, as we previously demonstrated for our NPFF piperidine-based scaffolds [29]. AstraZeneca reported a series of N,N-diethyl-4-(phenylpiperidin-4-ylidenemethyl)benzamides [30] as high affinity delta opioid receptor agonists (Figure 1). This opioid scaffold was chosen to install NPFF-Rs antagonist activity, as an additional phenyl ring at the 4-position of the piperidine ring is tolerated on our NPFF-binding guanidino–piperidine scaffold, affording analogs with reduced albeit measurable NPFF1-R and NPFF2-R affinity (0.9–2.4 µM) [29,30,31,32,33]. To this end, the 4-N,N-diethylaminocarbonyl group of the core 4-(diphenylmethylene)piperidine opioid scaffold was replaced with an NPFF pharmacophoric guanidine moiety [29,31].

We report here the synthesis of a series of designed ligands on a guanidino–piperidine scaffold and the biological evaluation of affinity, selectivity, and efficacy that led to the first small molecule opioid/NPFF-R DMLs. (Figure 1).

## 2. Results

### 2.1. Chemistry: Synthesis of the Opioid Agonist/NPFF Antagonist DML

**Scheme 1:**
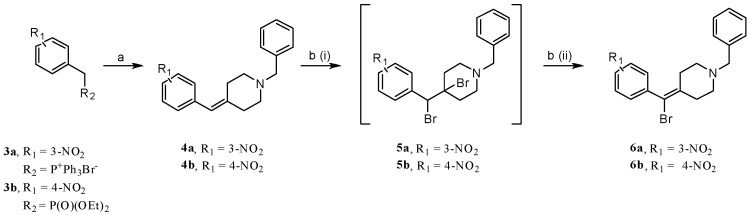
Synthesis of vinyl bromo intermediates **6a–b**. Reagents and conditions: (a) LDA, 1-benzyl-4-piperidone, THF_(ah)_, 19–67%; (b) (i) Br_2_, CH_2_Cl_2_, rt; (ii) NaOH, MeOH, 40 °C, 50–53%.

**Scheme 2:**
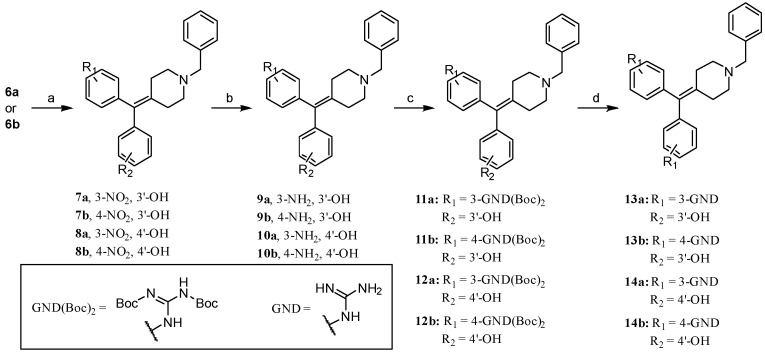
Synthesis of di-substituted 4-(diphenylmethylene)piperidines **13a–b** and **14a–b**. Reagents and conditions: (a) 3- or 4-hydroxyphenylboronic acid, 2 mol% Pd(PPh_3_)_4_, K_2_CO_3_, C_6_H_6_/EtOH 3/1, reflux, 50–93%; (b) 2.7 atm H_2(*g*)_, Pd/C 10%, MeOH, rt, 43–80%; (c) HgCl_2_, 1,3-bis(tert-butoxycarbonyl)-2-methyl-2-thiopseudourea, Et_3_N, CH_2_Cl_2_, rt, 27–74%; (d) TFA, CH_2_Cl_2_, rt, 17–79%.

To explore various positions for the hydroxyl and guanidine moieties, the synthesis of four regioisomers was carried out. Di-substituted diphenylmethylidene piperidines **13a–b** and **14a**–**b**, containing hydroxyl and guanidine groups placed at the 3- and 4- positions, were synthesized using a previously reported synthetic approach (Scheme 1 and Scheme 2) [31,34]. Wittig or Horner–Wadsworth–Emmons olefination of **3a** and commercially available **3b** with 1-benzyl-4-piperidone afforded 3- and 4-nitro substituted monophenylmethylidene piperidines **4a**–**b** [35]. Dibromination of **4a–b** gave unstable analogs **5a–b**, which then underwent in situ elimination with NaOH to give vinyl bromo intermediates **6a–b** (Scheme 1). Suzuki coupling of **6a–b** with 3- and 4-hydroxyphenylboronic acid afforded disubstituted diphenylmethylidene piperidines **7a–b** and **8a–b**. Reduction of the nitro group in **7a–b** and **8a–b** through catalytic hydrogenation afforded anilines **9a–b** and **10a–b**, which were subjected to HgCl_2_-assisted guanidinylation to yield Boc-protected guanidines **11a–b** and **12a–b** [36]. Finally, deprotection with trifluoroacetic acid afforded the desired disubstituted regioisomers **13a–b** and **14a–b** (Scheme 2).

**Scheme 3:**
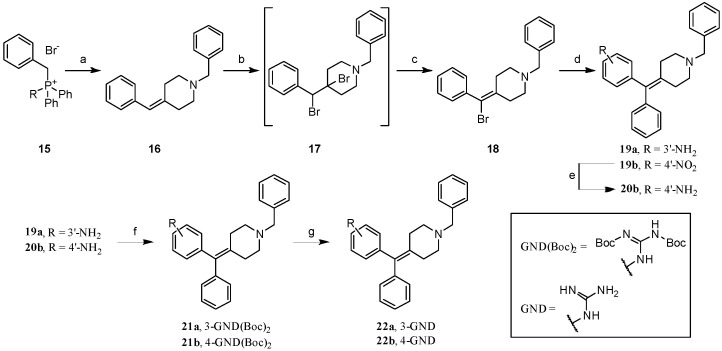
Synthesis of mono-substituted 4-(diphenylmethylene)piperidines **22a–b**. Reagents and conditions: (a) LDA, N-benzylpiperidone, THF(ah), 69%; (b) Br2, CH2Cl2, 67%; (c) NaOH, MeOH, 40 °C, 89%; (d) 3-aminophenylboronic acid hydrochloride or 4-nitrophenylboronic acid, 2 mol% Pd(PPh_3_)_4_, K_2_CO_3_, C_6_H_6_/EtOH 3/1, reflux, 70–98%; (e) 2.7 atm H_2*(g)*_, 10% Pd/C, MeOH, 95%; (f) HgCl_2_, 1,3-bis(tert-butoxycarbonyl)-2-methyl-2-thiopseudourea, Et_3_N, CH_2_Cl_2_, 71–75%; (g) TFA, CH_2_Cl_2_, or 4N HCl in dioxane, CH_2_Cl_2_ 49–74%.

Utilizing an approach similar to that described above (Scheme 1), monosubstituted analogs **22a** and **22b** (Scheme 3) were prepared starting with the Wittig olefination of commercially available salt **15** with 1-benzyl-4-piperidone to afford the unsubstituted monophenylmethylidene piperidine **16** with considerably higher yield (69%) than the corresponding nitro analogs **4a–b**. Dibromination of olefin **16** again afforded the unstable intermediate **17**, which, upon subsequent in situ elimination with NaOH, furnished the vinyl bromide **18** with 89% yield. Similar chemistry as described in Scheme 2 was then employed to ultimately afford 3- and 4- guanidine regioisomers **22a** and **22b** in decent yields (Scheme 3).

### 2.2. Determination of Binding Affinity of Lead Analogs at NPFF1-R, NPFF2-R, MOR, DOR, and KOR

Evaluation of compounds **13a–b**, **14a–b**, and **22a–b** in radioligand competition binding assays at opioid and NPFF receptors (Table 1; see also Figure S1 in the Supporting Information) identified binding affinities ranging from low nanomolar to low micromolar values at all five targets. Within the 3-guanidino series, the 3′-hydroxy derivative **13a** showed the highest affinities at all of the opioid receptors. Shifting the hydroxyl group into the 4′- position (**14a**) reduced affinity at MOR by 10-fold, at DOR by 4-fold, and at KOR by 11-fold. Conversely, the presence (**13a**) or absence (**22a**) of the 3′-hydroxyl group did not have a significant effect on the binding at MOR, but decreased affinity at DOR and KOR. Similarly, in this series, NPFF1-R and NPFF2-R seemed to be significantly unaffected by the absence or presence of the hydroxyl group in either position 3′- or 4′-, with the compounds **13a**, **14a**, and **22a** showing K_i_ variations generally smaller than one-fold. Within the 4-guanidino series, the 4′-hydroxyl (**14b**) group did not significantly affect opioid binding, but shifting the hydroxyl group to position 3- (**13b**) somewhat increased the affinity at all opioid receptors compared to the unsubstituted derivative **22b**. On the contrary, affinity for NPFF2-R was negatively affected by the presence of the hydroxyl group at either the 3′- or 4′- position (**13b** and **14b**, respectively), while affinities for NPFF1-R remained within a one-fold difference. Shifting the guanidine group from the 3- to the 4- position had a limited effect on the binding at both DOR and KOR (1- to 2-fold decrease in affinity), whereas MOR binding was more negatively affected (an approximately 10-fold decrease in affinity). Interestingly, the change from the 3- to the 4- position of the guanidine group somewhat increased affinity at NPFF1-R for the unsubstituted and 3′-hydroxyl substituted compounds (**22a–b** and **13a–b**, respectively) but had no significant effect for the 4′-hydroxyl substituted compounds (**14a–b**). Conversely, shifting the position of the guanidino group from the 3- to the 4- position had limited to no effect on the affinities at NPFF2-R when a hydroxyl group was present (**13a–b** and **14a–b**), but led to a 10-fold increase in affinity among the unsubstituted compounds **22a–b**. Among the series, compound **22b** showed the most balanced affinities at the targets of interest, NPFF1-R, NPFF2-R, and MOR.

### 2.3. Determination of Functional Efficacy of Lead Analogs at NPFF1-R, NPFF2-R, MOR, DOR, and KOR

Compounds **13a–b**, **14a–b**, and **22a–b** demonstrated no agonistic activity at both NPFF receptors (see Table S1 of the Supporting Information); the functional profile at the opioid receptors was more varied, and the substitution pattern had a greater impact on both activity and receptor subtype selectivity (Table 2; see also Figure S2 in the Supporting Information). In fact, moving the hydroxyl group from the 3′- (**13a–b**) to the 4′- position (**14a–b**) reduced the overall activity at most of the opioid receptors, with the exception of the 4-guanidino compound **14b**, which showed a small but significant increase in KOR efficacy. Shifting the guanidine group from the 3- (**13a**, **14a**, **22a**) to the 4- position (**13b**, **14b**, **22b**) generally decreased the activity at MOR. This effect also modulated the type of activity the analogs were showing, transforming the full agonist **13a** into the partial agonist **13b** and the partial agonist **14a** into the antagonist **14b**. The same modification also moderately reduced efficacy at DOR and KOR, with the exception of compound **14b**; these changes resulted in a small increase in efficacy at both DOR and KOR. Among the series of compounds, **22b** showed the greatest selectivity for MOR and DOR over the undesired agonism at KOR, which leads to dysphoric effects.

### 2.4. In Vivo Screening with the Warm Water Tail Withdrawal Assay

**Figure 2:**
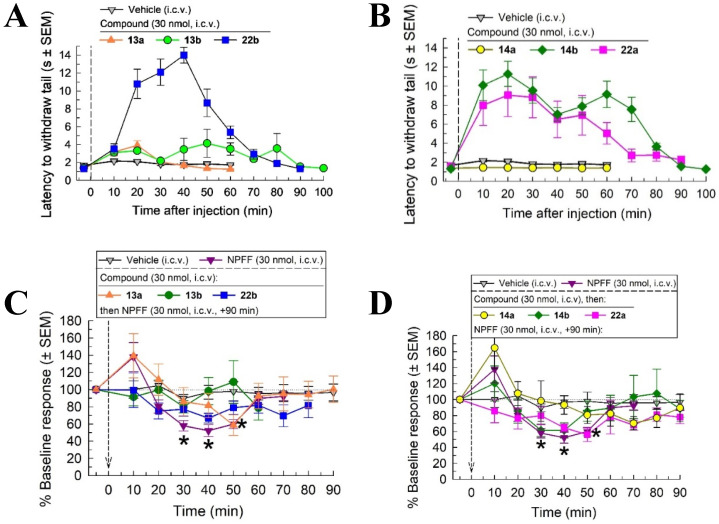
Screening of compound-induced antinociception and prevention of NPFF-induced hyperalgesia. Following i.c.v. administration of a 30 nmol dose to C57BL/6J mice, antinociceptive activity of **13a**, **13b,** and **22b** (**A**) and **14a**, **14b,** and **22a** (**B**) was measured repeatedly over time using the 55 °C warm water tail flick withdrawal assay. Two hours after administration of **13a**, **13b,** and **22b** (**C**) and **14a**, **14b,** and **22a** (**D**), mice were administered NPFF (30 nmol i.c.v.; dashed arrow). Hyperalgesic response was assessed by measuring the latencies to withdraw their tails from a 48 °C warm water stimulus measured repeatedly for 90 min. Administration of vehicle (3% DMSO, i.c.v., white triangles) alone had no significant effect in either assay. Data on the left of the arrow represent baseline (pretreatment) response in each assay. Graphs present the mean latency to withdraw the tail ± SEM from 8 mice per group (**A**,**B**) or % baseline response ± SEM for 7–12 mice per group and 17 mice for NPFF (**C**,**D**). * *p* < 0.05 compared with the baseline response with one-way ANOVA with Tukey’s HSD post hoc test.

The antinociceptive activity over time of compounds **13a–b**, **14a–b**, and **22a–b** was screened in vivo following intracerebroventricular (i.c.v.) administration of a 30 nmol dose to C57BL/6J mice and repeated testing in the 55 °C warm water tail withdrawal assay. Compound **13a**, which showed the best activity at all three opioid receptors in vitro, did not show statistically significant antinociceptive activity in vivo, whereas compound **22b**, which was moderately active at mu and delta, and weaker at kappa opioid receptors in vitro, induced the greatest tail withdrawal latencies in vivo (Figure 2A). Compounds **14b** and **22a**, which had a similar in vitro profile at DOR and KOR but opposite effects at MOR, displayed antinociception in vivo second only to **22b** (Figure 2B). Interestingly, compound **14a** did not produce statistically significant antinociception despite possessing an in vitro activity profile similar to **22b** at MOR and DOR and being five-fold more active at KOR.

Each compound demonstrated the ability to prevent NPFF-induced hyperalgesia in the 48 °C warm-water tail-withdrawal assay, with **13b** and **14a** showing the greater anti-NPFF activity among the compounds in the series (Figure 2C,D); more moderate, yet still statistically significant, effect was observed for compounds **13a**, **14b**, **22a** and **22b**. Compound **22b** showed the greatest analgesic efficacy when compared to the other compounds while preventing NPFF-induced hyperalgesia and was consequently selected for further investigation.

### 2.5. In Vivo Pharmacological Characterization of **22b**

**Figure 3:**
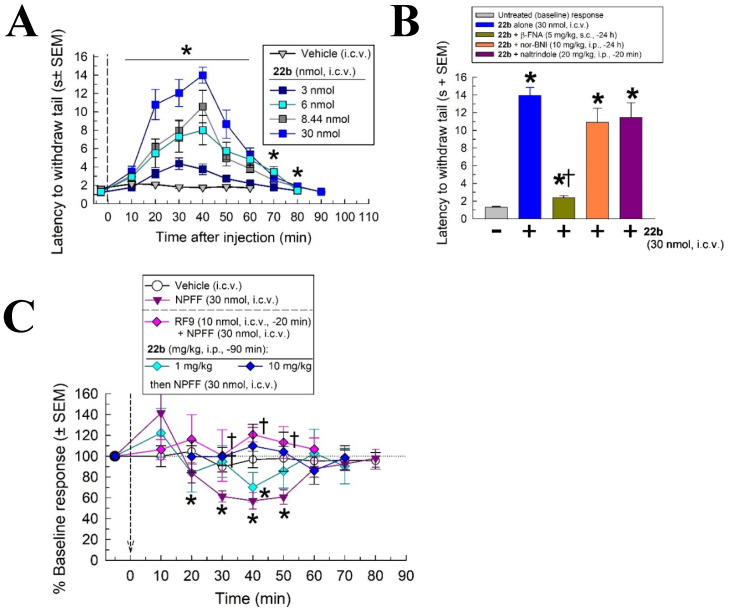
Pharmacological characterization of **22b** for antinociception, anti-hyperalgesia, and receptor selectivity. Following i.c.v. administration of a graded dose (3–30 nmol) of **22b** to C57BL/6J mice, latency to withdraw the tail was measured repeatedly over time using the 55 °C warm water tail withdrawal assay (**A**). The i.c.v. administration of vehicle alone (3% DMSO) had no significant effect. Opioid receptor selective agonism of **22b** (30 nmol, i.c.v.) was assessed in mice 24 h after pretreatment with β-FNA (5 mg/kg, s.c.) or nor-BNI (10 mg/kg, i.p.) or 20 min after pretreatment with naltrindole (20 mg/kg, i.p.). Tail withdrawal latencies were measured in mice 40 min after administration of **22b** (**B**). Two hours after administration (i.p.) of **22b** (**C**), mice were administered NPFF (30 nmol i.c.v.; en dashed arrow). Hyperalgesic response was assessed by measuring the latencies to withdraw their tails from a 48 °C warm water stimulus measured repeatedly for 90 min. Data left of arrow represent baseline (pretreatment) response in each assay. Graphs present the mean latency to withdraw the tail ± SEM from 8 mice per group (**A**,**B**) or % baseline response ± SEM for 8 mice per dose and 17 mice for NPFF alone (**C**). * *p* < 0.05 compared with the baseline response with one-way ANOVA with Tukey’s HSD post hoc test; *† *p* < 0.05 compared with both baseline response and **22b** effect alone with one-way ANOVA; † *p* < 0.05 compared with NPFF response with two-way ANOVA.

Compound **22b** was evaluated in vivo in the 55 °C warm water tail withdrawal antinociceptive assay to assess its opioid agonist activity after i.c.v. administration. Compound **22b** exhibited dose-dependent antinociception with an ED_50_ value (and 95% confidence interval) of 6.88 (4.71–9.47) nmol, i.c.v., 40 min after administration (Figure 3A). Over the first 90 min, **22b** exhibited significant antinociception dependent on dose and time (F_(36,315)_ = 9.64, *p* < 0.0001; two-way RM ANOVA), whereas the vehicle proved ineffective. The antinociceptive activity of **22b** was surprising, given the modest in vitro agonist activity in the [^35^S]GTPγS assay and the relatively low opioid receptor affinity. Therefore, we evaluated whether the antinociceptive activity was mediated through opioid receptors by pretreating mice with antagonists selective for MOR, KOR, and DOR. Pretreatment with the MOR-selective antagonist β-funaltrexamine (β-FNA) almost completely abolished the antinociceptive activity of **22b** (F_(4,59)_ = 75.8, *p* < 0.0001, one-way ANOVA with Tukey’s HSD post hoc test; Figure 3B). In contrast, pretreatment with the KOR-selective antagonist nor-binaltorphimine (nor-BNI) or the DOR-selective antagonist naltrindole did not alter the antinociceptive response. Together, these results suggest that the antinociceptive activity of **22b** is almost exclusively mediated by MOR. Notably, **22b** produced rapid, dose-dependent antinociception after i.p. administration with an ED_50_ value (and 95% confidence interval) of 3.61 (2.52–5.30) mg/kg, i.p., 10 min after administration, but the effect lasted no more than 50 min. Peripheral pretreatment for 90 min with **22b** also dose-dependently prevented NPFF-induced hyperalgesia, with significant antagonism after a 10 mg/kg i.p. pretreatment (F_(24,295)_ = 1.90, *p* = 0.008; two-way ANOVA; Figure 3C). The NPFF-R antagonism of **22b** was equivalent to those observed after a 20 min i.c.v. pretreatment with RF9, the NPFF-R inhibitor reported to prevent the development of acute opioid tolerance [17].

### 2.6. Evaluation of Potential Opioid-like Liabilities of **22b**

**Figure 4:**
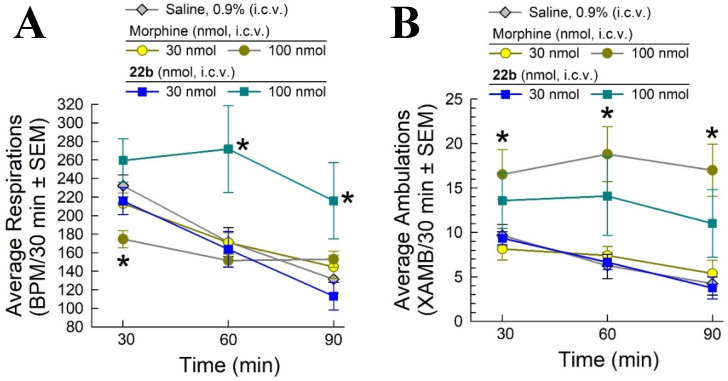
Respiratory and locomotor effects of **22b**. Mice were administered vehicle (0.9% saline; white diamonds), a graded 30 or 100 nmol i.c.v. dose of **22b** (blue squares), or morphine (yellow circles), and the effect was measured for 90 min on (**A**) respiration rate (as breaths/min (BPM)) and (**B**) spontaneous locomotor effects (ambulations/min) in the Comprehensive Lab Animal Monitoring System (CLAMS). * = significant difference from the vehicle-treated control group (*p* < 0.05; two-way ANOVA with Tukey’s HSD post hoc test). Points equal 8 mice (for doses of **22b**), 11 mice (for vehicle), and 12 mice (for doses of morphine).

A 90 min pretreatment with morphine significantly reduced the number of excreted fecal boli compared to saline-treated mice (1.8 ± 0.8 (30 nmol) and 1.5 ± 0.6 (100 nmol) vs. 5.6 ± 1.0 boli for vehicle-treated mice; F_(4,29)_ = 3.20, *p* = 0.03; one-way ANOVA with Dunnett’s multiple comparison post hoc test), consistent with the reduced intestinal motility attributed to MOR agonists. In contrast, **22b** treatment was without significant effect on excreted boli (3.5 ± 0.7 and 4.1 ± 0.6 for 30 and 100 nmol, respectively). The effect of **22b** on spontaneous locomotor activity and respiration rate was assessed over 90 min after i.c.v. administration of a therapeutic (30 nmol) or supratherapeutic (100 nmol) dose using the Comprehensive Lab Animal Monitoring System (CLAMS). As expected, treatment with morphine (100 nmol, i.c.v.) produced both significant respiratory depression (F_(4,138)_ = 10.6, *p* < 0.001; two-way ANOVA with Tukey’s HSD post hoc test; Figure 4A) and increased ambulation (F_(4,138)_ = 15.0, *p* < 0.0001; two-way ANOVA with Tukey’s HSD post hoc test; Figure 4B).

In contrast, no marked reduction in respiration was observed following administration of **22b** at the 30 nM dose; conversely, an increased respiration rate was evident after treatment with the higher dose of 100 nmol (i.c.v.). Although **22b** dose-dependently increased ambulation, the response following a 100 nmol, i.c.v. dose was not significantly greater than that of the vehicle, unlike that obtained with morphine (Figure 4B).

### 2.7. Assessment of Antinociceptive Tolerance

**Figure 5:**
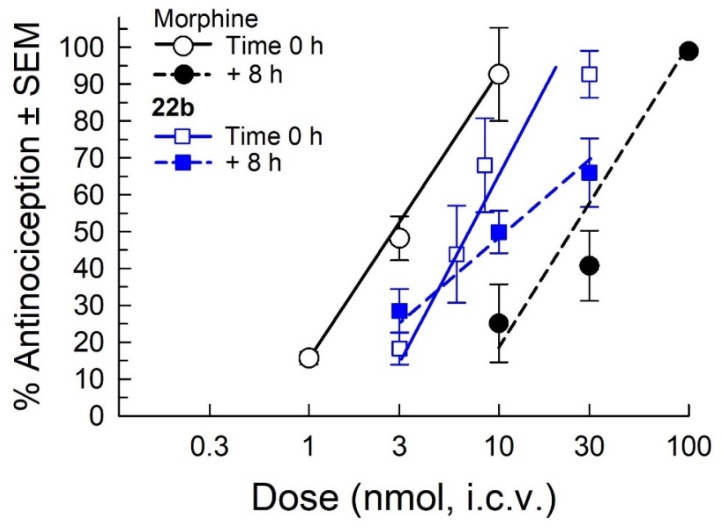
Acute tolerance development of **22b** compared with morphine. Repeated administration of **22b** did not demonstrate acute antinociceptive tolerance in the 55 °C warm water tail withdrawal assay. Morphine (3 nmol, i.c.v., open circles) or **22b** (8.7 nmol, i.c.v., open squares) was administered at time 0, followed 8 h later by a graded dose (3–100 nmol, i.c.v.) of the same compound. At 8 h, significant tolerance developed to morphine, demonstrated by a 9.6-fold rightward shift in the ED_50_ value (filled circles), but the 1.6-fold rightward shift produced by **22b** was not significant (filled squares). (n = 7–8 mice/point).

NPFF receptor antagonism has been reported to prevent opioid-induced antinociceptive tolerance [17,22], suggesting that a dual-acting opioid agonist/NPFF antagonist like **22b** may show less tolerance than a MOR agonist like morphine. To test this, an acute tolerance assay [37] was performed with centrally administered (i.c.v.) **22b** in the 55 °C warm water tail withdrawal assay. Morphine produced dose-dependent antinociception with an ED_50_ value (and 95% CI) of 2.35 (1.13–5.03) nmol, but the antinociceptive dose response of a second administration of morphine given 8 h after a first dose of 3 nmol, i.c.v., was shifted 9.6-fold rightward (ED_50_ value and 95% CI = 22.5 (8.48–61.9) nmol), demonstrating significant acute antinociceptive tolerance (F_(2,112)_ = 9.96, *p* < 0.0001, nonlinear regression analysis; Figure 5). In contrast, a second administration of varying doses of the dual opioid agonist–NPFF antagonist **22b** given 8 h after an 8.7 nmol (i.c.v.) dose resulted in dose-dependent antinociception with an ED_50_ (and 95% CI) value of 10.8 (5.89–21.1) nmol, a 1.6-fold rightward shift that did not differ significantly from either the morphine or the **22b** baseline responses (F_(1,81)_ = 0.55, *p* = 0.46, nonlinear regression analysis).

### 2.8. Pharmacokinetic (PK) Studies

The metabolic stability of **22b** (1 µM) was studied in vitro using Sprague Dawley rat liver microsomes (RLM), showing that 25.5% of the compound remained unchanged after 1 h of incubation, with an in vitro half-life of 31.0 ± 0.8 min (see Figure S3 of the Supporting Information). The intrinsic clearance (CL_int_) was 0.045 ± 0.001 mL/min per milligram of microsomal protein in pooled rat liver microsomes. These in vitro microsomal stability results prove that the compound was relatively stable in liver microsomes. However, metabolism of the compound was also observed in the negative control reaction mixture (without NADPH), indicating that additional non-cytochrome P_450_ mediated mechanisms could be responsible for the compound’s loss. For the evaluation of the in vivo pharmacokinetic parameters of **22b**, a single-dose PK study was performed with male adult Sprague Dawley rats. The plasma concentration–time profile after intravenous administration of **22b** (5 mg/Kg) showed a bi-exponential distribution (Figure S5 of the Supporting Information), and a large volume of distribution (V_d_ = 2.0 ± 0.2 L) was observed, indicating extensive extravascular distribution of the compound. Adequate systemic exposure (AUC/dose) of **22b** was estimated to be 3004.7 ± 108.1 h*ng/mL/mg, with low clearance (CL, 0.3 ± 0.0 L/h) and moderate elimination half-life (4.1 ± 0.5 h) (Table 3).

## 3. Discussion

From the published findings with RF9 and heroin, NPFF is strongly implicated in the development of opioid antinociceptive tolerance. Accordingly, a DML approach targeting both opioid and NPFF receptors is appealing both in terms of PK predictability and management for future development as a pain treatment alternative to the present clinical options available [17,25]. This study demonstrates that simple substitutions could be swiftly introduced on the chosen guanidino–piperidine scaffold and that such substitutions were able to direct both activity and selectivity at the different opioid receptor subtypes (Table 2). Specifically, in the presented examples, the relative positions of the guanidine and hydroxyl groups seem to play an important role in selectivity between mu, delta, and kappa opioid receptors. Removal of the hydroxyl group generally resulted in a moderate but significant increase in binding at the NPFF receptors, again indicating that simple modification of the substitution patterns could modulate the activity ratio between opioid and NPFF receptors. A methodical focus on such substitutions could accomplish a pivotal objective of optimizing the ratio of opioid agonism and NPFF-R antagonism during the later stage of compound development, suggesting a target for future studies. Moreover, while our previous [29] and present work suggests that a positively charged group (i.e., guanidine) is essential for NPFF receptor recognition, the neutral scaffold has not yet been investigated, suggesting an additional target for future studies.

In addition to the in vitro pharmacological studies, each compound was evaluated in vivo for antinociception and anti-hyperalgesic properties to obtain preliminary data for each member of the series (specifically, **13a–b**, **14a–b**, and **22a–b**). Central administration was utilized to initially measure the inherent activity of the ligands while minimizing pharmacokinetic factors. In the warm water tail withdrawal assay, compounds **14a** and **13b** were able to antagonize NPFF-induced hyperalgesia but lacked any marked analgesic activity (Figure 2A,B). However, **22b** showed the highest analgesic efficacy and significantly reduced NPFF-induced hyperalgesia (Figure 2A–D). These results highlight a poor correlation between activities in vitro and in vivo, which will require further investigation to clarify, but which are not uncommon when comparing the results of non-physiological and physiological assays of drug characterization [38]. The biological complexity of whole organisms includes the interplay of different organs, systems, and physiological processes, like absorption, distribution, metabolism, and excretion, all of which may account for the differences from membrane- or cell-based assays, but which are beyond the scope of this initial synthesis and characterization. Regardless, the present findings clearly establish the analgesic properties of compounds **14b**, **22a,** and, in particular, **22b**. Interestingly, the differing in vitro receptor subtype selectivity of **22a** and **22b** at the NPFF receptors and their similar in vivo anti-hyperalgesic profile could support the hypothesis that both NPFF1 and NPFF2 receptors may be involved in NPFF-mediated hyperalgesia. These findings are in contrast to recent studies with the peptide BN-9, a dual opioid and NPFF-Rs agonist that prevented the development of analgesic tolerance and opioid-induced hyperalgesia [39,40], which the authors attribute to the possible involvement of NPFF receptor agonism instead of antagonism. Although the present findings are consistent with a number of additional reports demonstrating the absence of OIH in compounds possessing dual opioid agonism and NPFF antagonism [21,26], further work is needed to better understand this pharmacological relationship.

Our present in vivo studies confirmed that the dose-dependent antinociceptive effects of **22b** (Figure 3A) were primarily MOR-mediated (Figure 3B), whereas its anti-hyperalgesic activity was suggested to be due to NPFF-R antagonism (Figure 3C). Of interest, and in contrast to clinically used MOR agonists such as morphine, **22b** did not reduce breathing rates, and displayed significantly lower increases in ambulation rates than the response induced by morphine. Most importantly, **22b** alone produced significant analgesia with very limited development of acute antinociceptive tolerance in the animal model employed. Although more extensive testing, including effects in different mouse strains and assessment of β-Arrestin signaling by the lead compounds, would be of potential future interest, it is notable that these responses were similar to those reported following treatment with a combination of the mu opioid agonist morphine and the NPFF antagonist RF9 [17], further validating the feasibility of the current strategic approach.

Compound **22b** showed relatively low intrinsic clearance in the in vitro microsomal stability analysis, but the decay of the compound in the negative control reaction mixture (in absence of NADPH; see Figure S3 of the Supporting Information) indicates non-enzymatic mechanisms of compound loss. In the in vivo preclinical PK study using a bioanalytical method that was able to accurately quantify the compound in plasma up to 10 h after dosing, **22b** exhibited a very large apparent volume of distribution (V_d_ = 2.0 ± 0.2 L), which generally correlates to extensive tissue distribution. The semi-log graph of the plasma concentration–time profile (Figure S5 of the Supporting Information) indicates an apparent bi-exponential decay, which could be translated into a two-compartment PK model. The calculated clearance (CL) of 0.3 ± 0.0 L/h is lower than the average hepatic blood flow, which could indicate that there is no extrahepatic elimination present [41]. The presence of anti-hyperalgesic effects of **22b** after i.p. administration (Figure 3C) could suggest that the compound is able to pass the blood–brain barrier despite the presence of a charged guanidine group at physiological pH. However, further PK studies are required to fully characterize the distribution into various body compartments to allow for a clear PK-PD model representation of this new, promising lead compound.

## 4. Materials and Methods

### 4.1. Chemistry

Unless otherwise stated, reagents and starting materials were obtained from commercial suppliers and used without purification. Fresh anhydrous (ah) THF was obtained through distillation over sodium with benzophenone as the indicator. Pre-coated silica gel GF Uniplates (Analtech; Miles Scientific, Newark, DE, USA) were used for thin-layer chromatography (TLC). Column chromatography was performed with silica gel 60 (Sorbent Technologies, Norcross, GA, USA). The mass spectra (MS) were recorded on a WATERS ACQUITY Ultra Performance Liquid Chromatography (UPLC) with a ZQ detector (Waters Corporation, Milford, MA, USA) in Electro-Spray Ionization (ESI) mode. Furthermore, 1H and 13C spectra were obtained on a Bruker 500 MHz, 400 MHz, or Bruker 400 MHz Ultra Shield. The high-resolution mass spectra (HRMS) were recorded on a Waters Micromass Q-Tof Micromass spectrometer (Waters Corporation, Milford, MA, USA) with a lock spray source in ESI mode. High-Performance Liquid Chromatography (HPLC) analyses were determined on a Waters 2695 system coupled with a Waters 996 UV detector (Waters Corporation, Milford, MA, USA). Measurement conditions were as follows. Condition A: column: XTerra R8 (5 µm) column (4.6 × 100 mm). Mobile phase: gradient run from 30% of a 10% NH_4_OH(aq) and 70% of CH_3_CN; flow rate: 1 mL/min. Detection condition: 254 nm. Condition B: column: Waters X-Bridge C18 column. Mobile Phase: gradient run from 50% H_2_O, 40% CH_3_CN, and 10% H_2_O/TFA (99.9:0.1) to 0% H_2_O, 90% CH_3_CN, and 10% H_2_O/TFA (99.9:0.1) for 12 min; flow rate: 1 mL/min. Detection condition: 254 nm. All final compounds tested were confirmed to be of ≥95% purity through the HPLC methods described above, except for **13a** (94.0%).

*General procedure A—Wittig and Horner*–*Wadsworth*–*Emmons olefination*. To a mixture of the appropriate phosphonium bromide or phosphonate ester (34.62 mmol) in distilled THF(ah) (200 mL) under Ar*_(g)_* at −78 °C or 0 °C, respectively, was added, dropwise, a solution of 2 M LDA in THF/n-heptane/ethyl benzene (19.04 mL, 38.08 mmol), which was stirred at 0 °C for 0.5 h. To this mixture was added a solution of 1-benzyl-4-piperidone (34.62 mmol) in distilled THF(ah) (180 mL) using a cannula, and the reaction was stirred at 0 °C for 0.5 h and then at rt overnight. The mixture was quenched with sat. NH_4_Cl_(aq)_, extracted with EtOAc, and the combined organic layers were washed with sat. NaCl_(aq)_, dried over Na_2_SO_4_, filtered, and concentrated. The residue was purified through flash column chromatography using a gradient of hexane/diethyl ether of 95:5→60:40 as the eluent to afford the title material.

*General procedure B—Bromination*. Bromine (0.63 mL, 12.35 mmol) was added dropwise to a mixture of olefin (5.42 mmol) in CH_2_Cl_2_ (100 mL) at 0 °C under Ar*_(g)_* and stirred at 0 °C for 1.5 h until full conversion to the dibromo intermediate was evident through TLC. NaOH (2.96 g, 74.09 mmol,) in MeOH (250 mL) was added, and the reaction was heated to 40 °C for 1 h. The mixture was then cooled to rt and partitioned between H_2_O and EtOAc. The aqueous layer was then extracted with EtOAc, and the combined organic layers were washed with sat. NaCl_(aq)_, dried over Na_2_SO_4_, filtered, and concentrated. The residue was purified through flash column chromatography using a gradient of hexane/diethyl ether 9:1→6:4 as the eluent to afford the title material.

*General procedure C—Suzuki coupling*. Ar*_(g)_* was bubbled into a mixture of vinyl bromide (5.81 mmol), substituted phenylboronic acid (6.98 mmol), and K_2_CO_3_ (2.00 g, 14.54 mmol) in benzene/ethanol 3:1 (130 mL). Pd(PPh_3_)_4_ (2 mol%) was added to the reaction mixture, and the vessel was wrapped in tin foil and heated to reflux for 16 h. The mixture was then cooled to rt and partitioned between H_2_O and EtOAc, and the aqueous layer was extracted twice with EtOAc. The combined organic layers were washed with sat. NaCl_(aq)_, dried over Na_2_SO_4_, filtered, and concentrated. The residue was purified through flash column chromatography using a gradient of hexane/diethyl ether of 8:2→6:4 (for **7a–b**, **8a–b**, **19b**) or hexane/EtOAc 2:8 (for **19a**) as the eluent to afford the desired material.

*General procedure D—Reduction of nitro group*. To a solution of aryl nitro intermediate (1.90 mmol) in MeOH (190 mL) was added a slurry of 10% Pd/C (20 wt% of substrate), and the mixture was reacted at rt under a hydrogen atmosphere (2.7 atm) for 4–6 h. The mixture was filtered through a pad of Celite^®^, concentrated, and purified through flash column chromatography using a gradient of CH_2_Cl_2_/MeOH 100/0→96/4 (for **9a**), hexane/EtOAc 80/20→20/80 (for **10a** and **20b**), or hexane/diethyl ether 70/30→0/100 (for **9b** and **10b**) to afford the title material.

*General procedure E—Guanidinylation*. To a mixture of amine (1.86 mmol) in CH_2_Cl_2_ (25 mL) was added 1,3-bis(tert-butoxycarbonyl)-2-methyl-2-thiopseudourea (0.60 g, 2.05 mmol), HgCl_2_ (0.56 g, 2.05 mmol), and Et_3_N (0.8 mL, 5.59 mmol), and the reaction was stirred for 16 h at rt. The mixture was then filtered through a pad of Celite^®^ and concentrated. The residue was purified via flash column chromatography using a gradient of hexane/ethyl acetate of 90/10→60/40 (for **11a**, **12b,** and **21a**), CHCl_3_/MeOH 100/0→99/1 (for **12a**), or hexane/diethyl ether 8/20→40/60 (for **11b**, **21b**) to afford the title material.

*General Procedure F—Boc deprotection*. To a solution of Boc-protected intermediate in CH_2_Cl_2_ (0.03 M) was added a mixture of CH_2_Cl_2_/TFA (3:1 ratio), and the reaction was stirred at rt for 2–5 h. The mixture was concentrated and then purified via flash column chromatography using CHCl_3_ or a gradient of CH_2_Cl_2_/MeOH satd. with NH_3_ (100/0→70/30). The title material was converted into the corresponding HCl salt through the addition of 1.25 HCl/MeOH and then triturated with EtOAc and ACN.

*(3-Nitrobenzyl)triphenylphosphonium bromide* (**3a**). 3-Nitrobenzyl bromide (30.00 g, 138.56 mmol) and PPh_3_ (36.42 g, 138.56 mmol) were dissolved in toluene (500 mL) and heated to reflux overnight. The suspension obtained was cooled to rt and filtered, and the precipitate was washed with toluene to afford the desired compound (63.24 g, 95% yield). ^1^H NMR: (400 MHz, DMSO-*d_6_*) δ 8.15–8.13 (m, 1H), 7.92–7.89 (m, 3H), 7.76–7.73 (m, 13H), 7.58–7.55 (m, 2H), 5.48 (d, *J_PH_* = 15.8 Hz, 2H). ^13^C NMR: (101 MHz, DMSO- *d_6_*) δ 147.9 (d, *J_PC_* = 3 Hz), 137.7, 135.8 (d, *J_PC_* = 2 Hz), 134.6 (d, *J_PC_* = 10 Hz), 130.8 (d, *J_PC_* = 31 Hz), 128.8, 126.0 (d, *J_PC_* = 5 Hz), 126.0, 123.7, 117.7 (d, *J_PC_* = 86 Hz), 28.1 (d, *J_PC_* = 46 Hz). MS(ESI+): 398.0 *m*/*z* [M]^+^.
*Diethyl (4-nitrobenzyl)phosphonate* (**3b**). 4-Nitrobenzyl bromide (5.00 g, 23.14 mmol) was suspended in triethylphosphite (16.0 mL, 92.57 mmol), heated to ca. 100 °C for 2 h, and then concentrated. The residue was purified via flash column chromatography using a gradient of hexanes/EtOAc to afford the desired product (4.98 g, 79% yield), which was spectroscopically equivalent to what was reported in reference [41]. ^1^H NMR: (400 MHz, CDCl_3_) δ 8.01 (d, *J* = 8.5 Hz, 2H), 7.39–7.33 (m, 2H), 3.97–3.87 (m, 4H), 3.13 (d, *J_PH_* = 22.3 Hz, 2H), 1.12 (t, *J* = 7.1 Hz, 6H). ^13^C NMR: (101 MHz, CDCl_3_) δ 146.8 (d, *J_PC_* = 4 Hz, C), 139.8 (d, *J_PC_* = 9 Hz, C), 130.5 (d, *J_PC_* = 6 Hz, CH), 123.5 (d, JPC = 3 Hz, CH), 62. 3 (d, *J_PC_* = 7 Hz, OCH_2_), 33.7 (d, *J_PC_* = 137 Hz, PCH_2_), 16.2 (d, *J_PC_* = 6 Hz, CH_3_).
*1-Benzyl-4-(3-nitrobenzylidene)piperidine* (**4a**). Prepared from **3a** according to general procedure A using (3-nitrobenzyl)(triphenyl)phosphonium bromide to afford the title compound with 19% yield. ^1^H NMR: (400 MHz, CDCl_3_) δ 8.06–8.04 (m, 2H), 7.53–7.43 (m, 2H), 7.40–7.23 (m, 5H), 6.31 (s, 1H), 3.56 (s, 2H), 2.59–2.43 (m, 8H). ^13^C NMR: (101 MHz, CDCl_3_) δ 148.2, 142.8, 139.5, 138.2, 134.9, 129.1, 128.9, 128.2, 127.1, 123.6, 121.1, 120.9, 62.8, 54.8, 54.1, 36.4, 29.1. MS(ESI+): 309.0 *m*/*z* (M+H)^+^.
*1-Benzyl-4-(4-nitrobenzylidene)piperidine* (**4b**). Prepared from **3b** according to general procedure A using diethyl(4-nitrobenzyl)phosphonate to afford the title compound with 67% yield. ^1^H NMR: (400 MHz, CDCl_3_) δ 8.17 (d, 2H, *J* = 8.7 Hz), 7.45–7.22 (m, 7H), 6.33 (s, 1H), 3.56 (s, 2H), 2.59–2.44 (m, 8H). ^13^C NMR (101 MHz, CDCl_3_) δ 145.9, 144.8, 144.2, 138.3, 129.5, 129.1, 128.3, 127.1, 123.5, 121.6, 62.8, 54.8, 54.1, 36.7, 29.4. MS(ESI+): 309.1 *m*/*z* (M+H)^+^.
*1-Benzyl-4-(bromo(3-nitrophenyl)methylene)piperidine* (**6a**). Prepared from **4a** according to general procedure B to afford the title material with 40% yield. ^1^H NMR (400 MHz, CDCl_3_) δ 8.15–8.13 (m, 2H), 7.61–7.59 (m, 1H), 7.54–7.49 (m, 1H), 7.32–7.31 (m, 4H), 7.28–7.26 (s, 1H), 3.54 (s, 2H), 2.72–2.70 (m, 2H), 2.59–2.56 (m, 2H), 2.45–2.37 (m, 2H), 2.30–2.22 (m, 2H). ^13^C NMR (126 MHz, CDCl_3_) δ 148.1, 141.9, 140.2, 138.1, 135.5, 129.3, 129.1, 128.3, 127.2, 124.5, 122.8, 112.2, 62.6, 53.9, 53.6, 34.5, 31.9. MS(ESI+): 388.9 *m*/*z* (M+H)^+^ for ^81^Br.
*1-Benzyl-4-(bromo(4-nitrophenyl)methylene)piperidine* (**6b**). Prepared from **4b** according to general procedure B to afford the title material with 27% yield. ^1^H NMR: (400 MHz, CDCl_3_) δ 8.20 (d, 2H, *J* = 8.6 Hz), 7.46 (d, 2H, *J* = 8.6 Hz), 7.40–7.19 (m, 5H), 3.56 (s, 2H), 2.77–2.68 (m, 2H), 2.65–2.56 (m, 2H), 2.45–2.37 (m, 2H), 2.34–2.25 (m, 2H). ^13^C NMR: (101 MHz, CDCl_3_) δ 147.1, 146.7, 140.3, 138.0, 130.5, 129.1, 128.3, 127.2, 123.6, 112.4, 62.6, 53.9, 53.6, 34.6, 32.1. MS(ESI+): 388.9 *m*/*z* (M+H)^+^ for ^81^Br.
*3-((1-Benzylpiperidin-4-ylidene)(3-nitrophenyl)methyl)phenol* (**7a**). Prepared from **6a** according to general procedure C using 3-hydroxyphenylboronic acid to give the title material with 80% yield. ^1^H NMR: (400 MHz, CDCl_3_) δ 8.07–8.00 (m, 1H), 7.94 (s, 1H), 7.42–7.41 (m, 2H), 7.35–7.22 (m, 6H), 7.16 (t, 1H, *J* = 7.8 Hz), 6.73–6.60 (m, 2H), 6.50 (s, 1H), 3.56 (s, 2H), 2.56–2.46 (s, 4H), 2.44–2.36 (m, 2H), 2.36–2.29 (m, 2H). ^13^C NMR: (101 MHz, CDCl_3_) δ 156.1, 148.0, 143.8, 142.5, 137.4, 136.9, 135.9, 133.7, 129.6, 129.5, 128.9, 128.3, 127.4, 124.4, 121.6, 121.4, 116.9, 114.3, 62.9, 54.7, 54.7, 31.2, 31.1. MS(ESI+): 401.0 *m*/*z* (M+H)^+^.
*3-((1-Benzylpiperidin-4-ylidene)(4-nitrophenyl)methyl)phenol* (**7b**). Prepared from **6b** according to general procedure C using 3-hydroxyphenylboronic acid to give the title material with 81% yield. ^1^H NMR: (400 MHz, DMSO-*d_6_*) δ 9.46 (bs, 1H), 8.14 (d, 2H, *J* = 8.4 Hz), 7.39–7.17 (m, 6H), 7.11 (t, 1H, *J* = 7.7 Hz), 6.66 (d, 1H, *J* = 7.3 Hz), 6.57–6.44 (m, 2H), 3.45 (s, 2H), 2.49–2.23 (m, 8H). ^13^C NMR: (101 MHz, DMSO-*d_6_*) δ 157.7, 149.5, 146.2, 142.6, 138.7, 138.1, 136.4, 134.0, 131.1, 129.8, 129.2, 128.6, 127.3, 123.8, 120.7, 116.8, 114.4, 62.3, 54.8, 31.9, 31.7. MS(ESI+): 401.1 *m*/*z* (M+H)^+^.
*4-((1-Benzylpiperidin-4-ylidene)(3-nitrophenyl)methyl)phenol* (**8a**). Prepared from **7a** according to general procedure C using 4-hydroxyphenylboronic acid to give the title material with 50% yield. ^1^H NMR: (400 MHz, DMSO-*d_6_*) δ 8.07–8.05 (d, 1H, *J* = 7.7 Hz), 7.79 (s, 1H), 7.59 (t, 1H, *J* = 7.9 Hz), 7.53–7.51 (m, 1H), 7.30–7.21 (m, 5H), 6.90–6.88 (m, 2H), 6.72–6.69 (m, 2H), 3.48 (s, 2H), 2.45–2.41 (m, 4H), 2.32–2.30 (m, 2H), 2.23–2.21 (m, 2H). ^13^C NMR: (101 MHz, DMSO--*d_6_*) δ 156.7, 148.1, 144.6, 138.6, 137.0, 136.6, 133.5, 132.0, 131.2, 130.1, 129.3, 128.6, 127.4, 124.1, 121.8, 115.6, 62.2, 54.8, 31.7. MS(ESI+): 401.1 *m*/*z* (M+H)^+^.
*4-((1-Benzylpiperidin-4-ylidene)(4-nitrophenyl)methyl)phenol* (**8b**). Prepared from **7b** according to general procedure C using 4-hydroxyphenylboronic acid to give the title material with 93% yield. ^1^H NMR: (500 MHz, DMSO-*d_6_*) δ 9.48 (s, 1H), 8.15 (d, 2H, *J* = 8.1 Hz), 7.32–7.24 (m, 7H), 6.88 (d, 2H, *J* = 7.8 Hz), 6.72 (d, 2H, *J* = 8.3 Hz), 3.48 (s, 2H), 2.49–2.12 (m, 8H). ^13^C NMR: (126 MHz, DMSO--*d_6_*) δ 156.8, 150.2, 146.2, 138.8, 137.6, 133.9, 131.9, 131.2, 129.3, 128.6, 127.4, 123.8, 115.6, 62.3, 54.9, 31.9. MS(ESI+): *m*/*z* 401.1 (M+H)^+^.
*3-((3-Aminophenyl)(1-benzylpiperidin-4-ylidene)methyl)phenol* (**9a**). Prepared from **7a** according to general procedure D to give the title material, which was used directly in the next reaction.
*3-((4-Aminophenyl)(1-benzylpiperidin-4-ylidene)methyl)phenol* (**9b**). Prepared from **7b** according to general procedure D to give the title material with 56% yield. ^1^H NMR: (500 MHz, CDCl_3_) δ 7.43–7.20 (m, 5H), 7.12 (t, 1H, *J* = 7.7 Hz), 6.84 (d, 2H, *J* = 7.9 Hz), 6.66 (d, 2H, *J* = 7.2 Hz), 6.56 (d, 2H, *J* = 7.9 Hz), 6.44 (s, 1H), 3.55 (bs, 4H), 2.35–2.50 (m, 8H). ^13^C NMR: (126 MHz, CDCl_3_) δ 155.8, 144.5, 144.5, 137.2, 135.5, 133.9, 132.8, 130.8, 129.7, 128.9, 128.2, 127.3, 121.8, 117.1, 114.7, 113.5, 63.0, 55.0, 54.9, 31.3, 31.2.
*4-((3-Aminophenyl)(1-benzylpiperidin-4-ylidene)methyl)phenol* (**10a**). Prepared from **8a** according to general procedure D to give the title material with 43% yield. ^13^C NMR: (126 MHz, DMSO-*d_6_*) δ 155.9, 148.5, 143.7, 138.4, 136.1, 133.6, 133.4, 130.8, 129.5, 128.9, 128.6, 127.4, 117.7, 115.5, 115.2, 112.6, 62.4, 55.0, 54.9, 31.7, 31.4. MS (ESI+): 371.2 *m*/*z* (M+H)^+^.
*4-((4-Aminophenyl)(1-benzylpiperidin-4-ylidene)methyl)phenol* (**10b**). Prepared from **7b** according to general procedure D to give the title material with 52% yield. ^1^H NMR: (500 MHz, CDCl_3_) δ 9.30 (s, 1H), 7.31–7.24 (m, 5H), 6.83–6.81 (m, 2H), 6.69–6.65 (m, 3H), 6.47–6.45 (m, 2H), 4.99 (s, 2H), 3.46 (s, 2H), 2.39–2.25 (m, 8H). ^13^C NMR: (126 MHz, CDCl_3_) δ 156.1, 147.4, 138.9, 135.9, 134.1, 132.6, 131.1, 130.7, 130.5, 130.5, 129.3, 128.6, 127.3, 115.1, 113.8, 62.6, 55.3, 55.2, 31.9.
*tert-Butyl N-[({3-[(1-benzylpiperidin-4-ylidene)(3-hydroxyphenyl)methyl]phenyl}amino)({[(tert-butoxy)carbonyl]imino})methyl]carbamate* (**11a**). Prepared from **9a** according to general procedure E to give the title material with 27% yield. ^1^H NMR: (400 MHz, CDCl_3_) δ 11.60 (bs, 1H), 10.20 (s, 1H), 7.55 (d, 1H, *J* = 7.9 Hz), 7.29 (dd, 5H, *J* = 12.6, 4.3 Hz), 7.19–7.05 (m, 3H), 6.79 (d, 1H, *J* = 7.6 Hz), 6.67–6.62 (m, 2H), 6.47 (s, 1H), 3.56 (s, 2H), 2.52–2.48 (m, 4H), 2.37 (dd, 4H, *J* = 22.2, 4.6 Hz), 1.61–1.38 (m, 18H). ^13^C NMR: (126 MHz, CDCl_3_) δ 163.5, 155.9, 153.7, 153.3, 143.4, 142.7, 136.2, 135.1, 129.6, 129.1, 128.6, 128.3, 127.3, 126.5, 123.7, 121.7, 120.9, 117.0, 113.8, 83.7, 79.7, 62.9, 55.0, 54.9, 31.2, 31.2, 28.2, 28.1. MS(ESI+): 613.2 *m*/*z* (M+H)^+^.
*tert-Butyl N-[({4-[(1-benzylpiperidin-4-ylidene)(3-hydroxyphenyl)methyl]phenyl}amino)({[(tert-butoxy)carbonyl]imino})methyl]carbamate* (**11b**). Prepared from **9b** according to general procedure E to give the title material with 74% yield. ^1^H NMR: (500 MHz, CDCl3) δ 11.64 (s, 1H), 10.29 (s, 1H), 7.48 (d, 2H, *J* = 8.4 Hz), 7.40–7.21 (m, 5H), 7.10 (t, 1H, *J* = 7.8 Hz), 7.01 (d, 2H, *J* = 8.4 Hz), 6.65–6.60 (m, 2H), 6.48 (s, 1H), 3.56 (s, 2H), 2.66–2.24 (m, 8H), 1.51 (s, 9H), 1.55 (s, 9H). ^13^C NMR: (126 MHz, CDCl3) δ 163.5, 155.9, 153.6, 153.3, 143.7, 138.9, 137.2, 135.2, 134.9, 130.3, 129.7, 129.1, 128.3, 127.3, 121.8, 117.1, 113.7, 83.7, 79.8, 62.9, 55.0, 54.9, 31.4, 31.2, 28.2, 28.1. MS(ESI+): 613.1 *m*/*z* (M+H)^+^.
*tert-Butyl N-[({3-[(1-benzylpiperidin-4-ylidene)(4-hydroxyphenyl)methyl]phenyl}amino)({[(tert-butoxy)carbonyl]imino})methyl]carbamate* (**12a**). Prepared from **10a** according to general procedure E to give the title material with 27% yield. ^1^H NMR: (500 MHz, CDCl_3_) δ 11.35 (s, 1H), 9.93 (s, 1H), 9.35 (s, 1H), 7.31–7.25 (m, 7H), 6.89–6.88 (m, 2H), 6.80 (d, 1H, *J* = 5.2 Hz), 6.69 (d, 2H, *J* = 7.7 Hz), 3.48 (s, 2H), 2.41–2.29 (m, 8H), 1.48–1.37 (m, 18H). ^13^C NMR: (126 MHz, CDCl3) δ 156.4, 153.2, 143.4, 139.0, 137.8, 136.7, 135.3, 135.0, 132.9, 131.0, 129.2, 128.7, 128.6, 127.3, 126.4, 124.5, 121.0, 115.3, 83.7, 62.5, 55.2, 54.9, 31.8, 31.7, 28.2, 28.2. MS(ESI+): 613.1 *m*/*z* (M+H)^+^.
*tert-Butyl N-[({4-[(1-benzylpiperidin-4-ylidene)(4-hydroxyphenyl)methyl]phenyl}amino)({[(tert-butoxy)carbonyl]imino})methyl]carbamate* (**12b**). Prepared from **10b** according to general procedure E to give the title material with 69% yield. ^1^H NMR: (500 MHz, DMSO-d6) δ 11.42 (s, 1H), 9.99 (s, 1H), 9.39 (s, 1H), 7.47 (d, 1H, *J* = 7.6 Hz), 7.31–7.25 (m, 5H), 7.02 (d, 2H, *J* = 7.8 Hz), 6.87–6.85 (m, 2H), 6.70–6.68 (m, 2H), 3.48 (s, 2H), 2.47–2.15 (m, 8H), 1.55–1.30 (m, 18H). ^13^C NMR: (126 MHz, DMSO-d_6_) δ 156.4, 153.1, 139.6, 138.9, 135.3, 135.3, 134.9, 133.1, 131.1, 130.2, 129.3, 128.6, 127.3, 122.6, 115.3, 62.5, 55.1, 31.8, 31.8, 28.2. MS(ESI+): 613.2 *m*/*z* [M+H]^+^.
*1-(3-((1-Benzylpiperidin-4-ylidene)(3-hydroxyphenyl)methyl)phenyl)guanidine* (**13a**). Prepared from **11a** according to general procedure F to give the title material as a hydrochloride salt with 40% yield. ^1^H NMR: (600 MHz, Methanol-*d_4_*) δ 7.49 (dd, *J* = 6.6, 3.0 Hz, 2H), 7.42–7.35 (m, 3H), 7.34 (t, *J* = 7.9 Hz, 1H), 7.10 (dd, *J* = 7.7, 2.0 Hz, 1H), 7.08–7.01 (m, 2H), 6.96 (t, *J* = 1.9 Hz, 1H), 6.60 (ddd, *J* = 8.2, 2.5, 0.8 Hz, 1H), 6.54 (dt, *J* = 7.4, 1.1 Hz, 1H), 6.49 (t, *J* = 2.0 Hz, 1H), 4.27 (s, 2H), 3.52–3.25 (m, 2H), 3.14–2.89 (m, 2H), 2.81–2.33 (m, 4H). ^13^C NMR: (151 MHz, Methanol-*d_4_*) δ 158.7, 158.0, 144.4, 143.3, 140.0, 136.3, 132.5, 131.2, 131.1, 130.6, 130.5, 130.3, 129.5, 127.2, 125.0, 121.5, 117.2, 115.4, 61.4, 54.2, 54.2, 29.4, 29.2. HRMS (ESI+): *m*/*z* calcd. for C_26_H_29_N_4_O [M+H]^+^ 413.2341, found 413.2348. Purity by HPLC: 94.0%, t_R_ = 3.2 min.
*1-(4-((1-Benzylpiperidin-4-ylidene)(3-hydroxyphenyl)methyl)phenyl)guanidine* (**13b**). Prepared from **11b** according to general procedure F to give the title material as a hydrochloride salt with 36% yield. ^1^H NMR: (500 MHz, Methanol-*d_4_*) δ 7.52–7.43 (m, 2H), 7.43–7.36 (m, 3H), 7.26–7.13 (m, 4H), 7.05 (td, *J* =7.9, 2.0 Hz, 1H), 6.60 (d, *J* =8.1 Hz, 1H), 6.54 (d, *J* =7.7 Hz, 1H), 6.51–6.43 (m, 1H), 4.26 (s, 2H), 3.42 (br s, 2H), 3.03 (br s, 2H), 2.69 (br s, 2H), 2.45 (br s, 2H). ^13^C NMR: (126 MHz, Methanol-*d_4_*) δ 158.7, 143.4, 141.8, 140.3, 135.1, 132.4, 132.0, 131.3, 130.6, 130.4, 126.3, 121.4, 117.2, 115.3, 61.5, 54.3, 54.2, 49.0, 29.4, 29.2. HRMS (ESI+) *m*/*z* calcd. for C_26_H_29_N_4_O [M+H]^+^ 413.2341, found 413.2333. Purity by HPLC: >99%, t_R_ = 3.84 min.
*1-(3-((1-Benzylpiperidin-4-ylidene)(4-hydroxyphenyl)methyl)phenyl)guanidine* (**14a**). Prepared from **12a** according to general procedure F to give the title material as a hydrochloride salt with 79% yield. ^1^H NMR: (600 MHz, Methanol-*d_4_*) δ 7.60 (dd, *J* = 6.6, 2.9 Hz, 2H), 7.53–7.48 (m, 3H), 7.46 (t, *J* = 7.8 Hz, 1H), 7.25–7.19 (m, 1H), 7.17 (dt, *J* = 7.7, 1.3 Hz, 1H), 7.06 (t, *J* = 1.9 Hz, 1H), 7.00 (d, *J* = 8.5 Hz, 2H), 6.77 (d, *J* = 8.5 Hz, 2H), 4.38 (s, 2H), 3.69–3.40 (m, 2H), 3.27–3.01 (m, 2H), 2.97–2.42 (m, 4H). ^13^C NMR: (151 MHz, Methanol-*d_4_*) δ 156.7, 156.6, 143.6, 138.6, 134.9, 131.6, 131.0, 130.4, 129.8, 129.6, 129.1, 129.0, 128.3, 128.2, 126.0, 123.6, 114.8, 60.1, 52.9, 28.1, 28.0. HRMS (ESI+) *m*/*z* calcd. for C_26_H_29_N_4_O [M+H]^+^ 413.2341, found 413.2325. Purity by HPLC: >99%, t_R_ = 2.21 min.
*1-(4-((1-Benzylpiperidin-4-ylidene)(4-hydroxyphenyl)methyl)phenyl)guanidine* (**14b**). Prepared from **12b** according to general procedure F to give the title material as a hydrochloride salt with 55% yield. ^1^H NMR: (500 MHz, Methanol-*d_4_*) δ 7.56 (dd, *J* = 6.4, 3.0 Hz, 2H), 7.52–7.43 (m, 3H), 7.24 (s, 4H), 6.96 (d, *J* = 8.1 Hz, 2H), 6.74 (d, *J* = 8.1 Hz, 2H), 4.35 (s, 2H), 3.48 (br s, 2H), 3.13 (br s, 2H), 2.95–2.34 (m, 4H). ^13^C NMR: (126 MHz, Methanol-*d_4_*) δ 156.6, 154.2, 141.0, 138.8, 133.5, 131.7, 131.0, 130.7, 130.3, 129.8, 129.1, 129.0, 128.0, 124.9, 114.8, 60.1, 52.9, 28.1, 28.0. HRMS (ESI+) *m*/*z* calcd. for C_26_H_29_N_4_O [M+H]^+^ 413.2341, found 413.2376. Purity by HPLC: >99%, t_R_ =3.80 min.
*Benzyltriphenylphosphonium bromide* (**15**). Benzyl bromide (10.4 mL, 87.7 mmol) and PPh_3_ (23.0 g, 87.7 mmol) were dissolved in toluene (500 mL) and heated to reflux overnight. The suspension obtained was cooled to rt and filtered. The precipitate was washed with toluene and dried to afford the desired compound (38.0 g, >99% yield). ^1^H NMR: (400 MHz, DMSO-*d_6_*) δ 7.94–7.85 (m, 3H), 7.79–7.65 (m, 12H), 7.29–7.26 (m, 1H), 7.20 (t, *J* = 7.5 Hz, 2H), 7.04–6.96 (m, 2H), 5.28 (d, JPH = 15.7 Hz, 2H). ^13^C NMR: (101 MHz, DMSO-d6) δ 135.6 (d, *J_PC_* = 3 Hz),134.5 (d, *J_PC_* = 10 Hz), 131.4 (d, *J_PC_* = 6 Hz), 130.5 (d, *J_PC_* = 12 Hz),129.2 (d, *J_PC_* = 3 Hz), 128.8 (d, *J_PC_* = 3 Hz), 128.5 (d, *J_PC_* = 9 Hz), 118.3 (d, *J_PC_* = 86 Hz), 28.6 (d, *J_PC_* = 46 Hz). MS(ESI+): *m*/*z* 353.99 [M]^+^.
*1-Benzyl-4-benzylidenepiperidine* (**16**). Prepared from **15** according to general procedure A to afford the title compound with 52% yield. ^1^H NMR: (400 MHz, CDCl_3_) δ 7.52–7.13 (m, 10H), 6.31 (s, 1H), 3.56 (s, 2H), 2.71–2.31 (m, 8H). ^13^C NMR: (101 MHz, CDCl_3_) δ 139.8, 138.6, 137.9, 129.2, 129.0, 128.2, 128.1, 127.0, 126.1, 123.2, 63.1, 55.1, 54.5, 36.6, 29.2. MS(ESI+): 264.2 *m*/*z* (M+H)^+^.
*1-Benzyl-4-(bromo(phenyl)methylene)piperidine* (**18**). Prepared from **16** according to general procedure B to afford the title compound with 29% yield. ^1^H NMR: (CDCl_3_) δ 7.36–7.29 (m, 10H), 3.56 (s, 2H), 2.73 (t, 2H, *J* = 5.4 Hz), 2.59 (t, 2H, *J* = 5.9 Hz), 2.40 (t, 2H, *J* = 5.1 Hz), 2.31 (t, 2H, *J* = 5.8 Hz). ^13^C NMR: (CDCl_3_) δ 140.6, 138.4, 137.8, 129.6, 129.3, 128.4, 128.4, 128.1, 127.2, 115.7, 62.9, 54.3, 54.0, 34.7, 32.1. MS (ESI+): 342.3 *m*/*z* (M+H)^+^ for ^79^Br, 344.9 *m*/*z* (M+H)^+^ for ^81^Br.
*3-((1-Benzylpiperidin-4-ylidene)(phenyl)methyl)aniline* (**19a**). Prepared from **18** according to general procedure C using 3-aminophenylboronic acid hydrochloride to give the title material with 98% yield. ^1^H NMR: (DMSO-*d_6_*) δ 7.30–7.15 (m, 8H), 7.06 (d, 2H, *J* = 7.9 Hz), 6.93 (t, 1H, *J* = 7.7 Hz), 6.40 (d, 1H, *J* = 8.0 Hz), 6.29–6.25 (m, 2H), 3.46 (s, 2H), 2.42–2.39 (m, 4H), 2.29–2.26 (m, 2H), 2.24–2.23 (m, 2H). ^13^C NMR: (DMSO- *d_6_*) δ 148.3, 142.7, 142.2, 138.4, 135.9, 134.2, 129.2, 128.8, 128.4, 128.1, 127.9, 126.8, 126.1, 116.9, 114.9, 112.1, 61.9, 54.6, 54.6, 31.3, 31.1. MS (ESI+): 355 *m*/*z* (M+H)^+^.
*1-Benzyl-4-((4-nitrophenyl)(phenyl)methylene)piperidine* (**19b**). Prepared from **18** according to general procedure C using 4-nitrophenylboronic acid to give the title material with 33% yield. ^1^H NMR: (400 MHz, CDCl_3_) δ 8.16 (d, 2H, *J* = 8.4 Hz), 7.45–7.22 (m, 10H), 7.15–7.13 (m, 2H), 3.60 (s, 2H), 2.66–2.52 (m, 4H), 2.51–2.37 (m, 4H). ^13^C NMR: (101 MHz, CDCl_3_) δ 149.5, 146.3, 138.5, 134.2, 130.8, 129.9, 129.3, 128.4, 128.3, 127.2, 127.0, 126.3, 123.4, 115.9, 62.9, 54.9, 54.9, 31.8, 31.8. MS(ESI+): 385.0 *m*/*z* (M+H)^+^.
*4-((1-Benzylpiperidin-4-ylidene)(phenyl)methyl)aniline (**20b**).* Prepared from **19b** according to general procedure D to give the title material with 95% yield. ^1^H NMR: (400 MHz, CDCl_3_) δ 7.44–7.08 (m, 10H), 6.92 (d, 2H, *J* = 8.3 Hz), 6.61 (d, 2H, *J* = 8.3 Hz), 3.61 (bs, 2H), 3.55 (s, 2H), 2.59–2.42 (m, 6H), 2.42–2.35 (m, 2H). ^13^C NMR: (101 MHz, CDCl_3_) δ 144.7, 143.1, 138.5, 135.6, 134.6, 133.0, 130.9, 129.9, 129.2, 128.2, 127.8, 127.0, 126.1, 114.6, 63.1, 55.3, 55.3, 31.8, 31.8. MS(ESI+): 355.1 *m*/*z* (M+H)^+^.
*tert-Butyl(3-((1-benzylpiperidin-4-ylidene)(phenyl)methyl)phenylamino)(tert-butoxycarbonylamino)methylenecarbamate* (**21a**). Prepared from **19a** according to general procedure E to give the title material with 75% yield. ^1^H NMR: (CDCl_3_) δ 11.61 (s, 1H), 10.21 (s, 1H), 7.57 (d, 1H, *J* = 8.1 Hz), 7.34–7.18 (m, 10H), 7.12 (d, 2H, *J* = 7.8 Hz), 6.87 (d, 1H, *J* = 7.4 Hz), 3.53 (s, 2H), 2.50–2.49 (m, 4H), 2.45–2.44 (m, 2H), 2.39–2.38 (m, 2H), 1.53 (s, 9H), 1.48 (s, 9H). ^13^C NMR: (CDCl_3_) δ 163.8, 153.7, 153.5, 143.2, 142.5, 138.7, 136.5, 136.4, 135.3, 130.0, 129.3, 128.7, 128.4, 128.1, 127.1, 126.7, 126.5, 123.9, 120.8, 83.8, 79.7, 63.2, 55.5, 55.3, 31.9, 28.4, 28.3. MS (ESI+): 597 *m*/*z* (M+H)^+^.
*Tert-butyl(4-((1-benzylpiperidin-4-ylidene)(phenyl)methyl)phenylamino)(tert-butoxycarbonylamino)methylenecarbamate* (**21b**). Prepared from **20b** according to general procedure E to give the title material with 71% yield. ^1^H NMR: (600 MHz, CDCl_3_) δ 11.65 (s, 1H), 10.33 (s, 1H), 7.54 (d, *J* = 8.5 Hz, 2H), 7.42–7.36 (m, 2H), 7.35 (t, *J* = 7.4 Hz, 2H), 7.32–7.25 (m, 3H), 7.21 (t, *J* = 7.4 Hz, 1H), 7.11 (d, *J* = 7.1 Hz, 2H), 7.08 (d, *J* = 8.6 Hz, 2H), 3.63 (s, 2H), 2.70–2.36 (m, 8H), 1.55 (s, 9H), 1.52 (s, 9H). ^13^C NMR: (151 MHz, CDCl_3_) δ 163.7, 153.5, 153.4, 142.4, 138.9, 135.8, 135.2, 130.5, 130.0, 129.6, 128.4, 128.1, 127.5, 126.5, 121.7, 83.8, 79.7, 62.9, 55.1, 31.5, 31.4, 28.3, 28.2. MS(ESI+): 597.1 *m*/*z* (M+H)^+^.
*1-(3-((1-Benzylpiperidin-4-ylidene)(phenyl)methyl)phenyl)guanidine* (**22a**). To a solution of intermediate **21a** (0.36 g, 0.587 mmol) in CH_2_Cl_2_ (20 mL) was added 4N HCl/dioxane (1.5 mL, 5.87 mmol), and the reaction mixture was stirred for 3 days at rt. The solvent was evaporated and the residue re-dissolved in 5% NaOH (aq) and then extracted with CH_2_Cl_2_ (3 × 20 mL). The combined organic layers were dried and evaporated, and the residue was purified via flash column chromatography using a gradient of CH_2_Cl_2_/MeOH satd. with NH_3_ 90:10→50:50 as the eluent to afford the title material, which was converted to the HCl salt through addition of HCl/dioxane (with presence of 1/2 molecule of dioxane, white solid, 0.21 g, 74%). ^1^H NMR: (500 MHz, Methanol-*d_4_*) δ 8.13 (s, 1H), 7.59–7.36 (m, 5H), 7.26 (t, *J* = 7.4 Hz, 2H), 7.23–7.15 (m, 5H), 7.09 (d, *J* = 7.3 Hz, 2H), 4.23 (s, 2H), 3.33–3.12 (m, 4H), 2.57 (dt, *J* = 16.5, 5.9 Hz, 4H). ^13^C NMR: (126 MHz, Methanol--*d_4_*) δ 156.6, 140.8, 140.5, 138.7, 133.7, 130.9, 130.7, 129.7, 129.5, 129.3, 129.0, 128.9, 128.2, 127.1, 124.9, 60.2, 52.9, 52.9, 28.1, 28.0. HRMS (ESI+) *m*/*z* calcd. for C_26_H_29_N_4_ [M+H]^+^ 397.2392, found 397.2728. Purity by HPLC: >99%, t_R_ =1.415 min.
*1-(4-((1-Benzylpiperidin-4-ylidene)(phenyl)methyl)phenyl)guanidine* (**22b**). Prepared from **21b** according to general procedure F to give the title material as a hydrochloride salt with 49% yield. ^1^H NMR: (500 MHz, Methanol-*d_4_*) δ 7.59–7.46 (m, 2H), 7.47–7.38 (m, 3H), 7.37 (t, *J* = 7.8 Hz, 1H), 7.26 (t, *J* = 7.4 Hz, 2H), 7.19 (t, *J* = 7.4 Hz, 1H), 7.17–7.08 (m, 4H), 7.03–6.96 (m, 1H), 4.29 (s, 2H), 3.54–3.39 (m, 2H), 3.16–2.99 (m, 2H), 2.71 (dd, *J* = 29.5, 15.1 Hz, 2H), 2.60–2.43 (m, 2H). ^13^C NMR: (126 MHz, DMSO-*d_6_*) δ 156.6, 141.4, 139.5, 137.5, 134.5, 131.9, 130.8, 130.7, 130.4, 129.8, 129.6, 129.2, 128.9, 127.5, 124.4, 59.0, 52.2, 52.2, 28.1, 28.0. HRMS (ESI+), *m*/*z* calc. for C_26_H_29_N_4_ [M+H]^+^ 397.2392, found 397.2412. Purity by HPLC: >99%, t_R_ = 3.89 min.

### 4.2. In Vitro Pharmacology

*Binding affinity at hNPFF1-R, hNPFF2-R, MOR, DOR, and KOR*. The affinities of **13a–b**, **14a–b**, and **22a–b** at NPFF1-R, NPFF2-R, MOR, DOR, and KOR were determined through radioligand competition experiments on membranes of Chinese hamster ovary (CHO) cells transfected with NPFF1 or NPFF2 receptors and HEK293 cell transfected with MOR, DOR, and KOR using [^3^H]NPVF, [^3^H]EYF, [^3^H]DAMGO, [^3^H]DPDPE, and [^3^H]U69,593 radioligands, respectively (Table 1) [29,37]. Affinities at NPFF1-R and NPFF2-R were compared to the high-affinity peptidic ligands NPFF, NPVF, and 1DMe and the non-peptidic ligand RF9, whereas affinities at the opioid receptors were compared to the well-established unselective antagonist naloxone (Table 1) [29].

*Functional efficacy at hNPFF1-R, hNPFF2-R, MOR, DOR, and KOR*. Functional behavior at NPFF1-R and NPFF2-R was determined by measuring the ability of compounds to inhibit forskolin-induced cAMP accumulation in transfected CHO cells compared with the agonists NPFF and NPVF. Functional activity at each opioid receptor subtype was determined by measuring the ability of compounds to stimulate [^35^S]GTPγS binding to the membranes of MOR, DOR, and KOR transfected into CHO cells compared with the standards DAMGO, DPDPE, and U-69,593, respectively (Table 2) [29].

### 4.3. In Vivo Behavioral Pharmacology

*Animals.* Adult (8–11 weeks old) male C57BL/6J mice were obtained from the Jackson Laboratory, Bar Harbor, ME (USA). All mice were housed five to a cage in a temperature- and humidity-controlled room at the University of Florida vivarium (Gainesville, FL, USA) on a 12:12 h light/dark cycle with free access to food and water, except during experimental sessions. All procedures were preapproved and carried out in accordance with the Institutional Animal Care and Use Committee at the University of Florida as specified by the 2008 National Institutes of Health Guide for the Care and Use of Laboratory Animals. Consistent with these guidelines, ongoing statistical testing of data collected was used to minimize the number of animals used, within the constraints of necessary statistical power. Subjects were assigned to groups randomly, and drug experiments were conducted in a blinded fashion.

*Injection techniques*. Intracerebroventricular (i.c.v.) injections were made directly into the lateral ventricle according to the modified steps detailed previously [29,42]. The volume of all i.c.v. injections was 5 μL, using a 10 μL Hamilton microliter syringe. The mouse was lightly anesthetized with isoflurane, an incision was made in the scalp, and the injection was made 2 mm lateral and 2 mm caudal to bregma at a depth of 3 mm.

*Antinociception assay (55 °C warm water tail withdrawal assay)*. Compounds were screened for antinociception according to the method previously described [42]. The nociceptive stimulus was 55 °C water, with latency to withdraw the tail taken as the end point. Briefly, each mouse was tested for baseline tail withdrawal latency; mice showing a baseline latency greater than 5 s were excluded from the study. Following baseline measurements, animals received i.c.v. or i.p. administration of the vehicle (saline, 0.9% or 3% DMSO/97% saline, 0.9% as specified) or graded doses of morphine or the test compound. Separate groups of mice were treated with different doses of the test compound, and individual mice were only used once for antinociceptive testing. Tail withdrawal latency was determined repeatedly every 10 min after administration. A maximum response time of 15 s was employed to prevent tissue damage. Where presented, the percentage of antinociception was calculated using the following equation: antinociception (%) = (test latency − base latency)/(15 − baseline latency) × 100.

*Anti-hyperalgesia assay (48 °C warm water tail withdrawal assay).* Compounds were screened for their ability to prevent NPFF-induced hyperalgesia, as described previously [29]. The nociceptive stimulus was 48 °C water, with latency to withdraw the tail taken as the end point. The water temperature of 48 °C was selected for this work to ensure a moderate tail withdrawal response with a measurable decrease in withdrawal time possible, but also a significant temperature for hyperalgesic testing. Animals showing an initial baseline latency of <4 s or >15 s were excluded from the study. A maximum response time of 30 s was used to prevent tissue damage. After determining control latencies, mice received a single i.c.v. dose of vehicle or drug. Data for hyperalgesic testing are reported as percentage baseline response to control for each animal’s baseline latency (± SEM) according to the following equation: Baseline response (%) = (test latency)/(baseline latency) × 100. Experimentally induced decreases in tail withdrawal latency indicate hyperalgesic effects [29,43,44]. Animals were pretreated with the compound approximately 1.5–2 h prior to administration of NPFF to exclude any interference with the analgesic effects.

*Respiration and locomotor testing.* Respiration and locomotion rates were monitored using the Comprehensive Lab Animal Monitoring System (CLAMS, Columbus Instruments, Columbus, OH) as previously described [42]. Mice were habituated to their individual sealed housing chambers for 60 min before testing. Mice were administered vehicle (saline, 0.9%), **22b,** or morphine, as indicated, and 5 min later confined in the CLAMS testing cages. Pressure monitoring within the sealed chambers measured the frequency of respiration. Infrared beams located in the floor measured locomotion as number of beam breaks. Ambulations and number of breaths were measured in 30 s intervals for 90 min.

*Excreta assay*. As a measure of intestinal transit [42], mice were pretreated i.c.v. with saline (0.9%), morphine, or **22b** (30 or 100 nmol each) and placed in individual cages without food or water. The number of fecal boli (excreta) was counted at the end of the 90 min period.

*Acute antinociceptive tolerance assay*. Compound **22b** was tested for tolerance development according to the method previously reported [42]. Briefly, morphine or **22b** were first administered i.c.v. at time 0 h, at a dose approximating the ED_50_ value (3 or 8.7 nmol, i.c.v., respectively). One of three graded second doses (3–100 nmol, i.c.v.) was administered again at 8 h. Antinociception was assessed 30 min after each injection in the 55 °C warm water tail withdrawal test. Previous reports have demonstrated that this dosing schedule induces relatively rapid and reliable acute morphine antinociceptive tolerance [42].

*Data analysis*. All data are presented as mean ± SEM, with significance set at *p* < 0.05. All data were statistically evaluated and graphically processed using GraphPad Prism 9.0 software (GraphPad Prism Software Inc., San Diego, CA, USA). Latency to withdraw the tail, rather than percentage of antinociception, was used to determine the statistical significance of within group effects, comparing baseline and post-treatment tail withdrawal latencies with Student’s *t*-tests (in screening tests) or through one- or two-way analysis of variance (ANOVA) with Dunnett’s post hoc test for **22b**. All tail withdrawal latency data for hyperalgesic testing are reported as percent baseline response to control for each animal’s baseline latency, ±SEM. Responses to treatment over time were analyzed through two-way ANOVA followed by Dunnett’s or Tukey’s honestly significant difference (HSD) post hoc test as appropriate, with factors of treatment and time as required. Additional tail withdrawal latency data in acute antinociceptive tolerance testing are reported as percent antinociception to control for each animal’s baseline latency response. All dose–response lines were analyzed through regression, and ED_50_ (dose producing 50% effect) values and 95% confidence limits were determined using each individual data point with Prism 9.0 software and compared using linear or nonlinear regression modeling, as appropriate. The degree of tolerance in experiments on acute antinociceptive tolerance was calculated from the shift in ED_50_ value from the singly treated to the repeatedly treated condition [45]. Data for respiration, locomotor, and excreta effects were further analyzed as needed with two-way ANOVA, with significant effects further analyzed through Tukey’s HSD post-hoc testing.

### 4.4. Metabolic Stability in Rat Liver Microsomes

In vitro *metabolism* of **22b** (1 µM) was studied using Sprague Dawley rat liver microsomes (RLM) in a bench-top shaker for 1 h at 37 ± 0.5 °C. An aliquot (2 μL) of the stock solution (250 µM) was spiked in 0.5 mL of pre-incubated metabolic reaction mixture [50 mM Tris buffer (pH 7.4), 0.5 mg protein/mL of RLM, 20 mM magnesium chloride, and 2 mM NADPH]. Verapamil was used as the positive control to ensure the activity of the liver microsomal enzymes, and a metabolic reaction mixture without NADPH was used as the negative control in the same incubation conditions. An aliquot (40 µL) of the reaction mixture was withdrawn at 0, 5, 10, 15, 30, 45, and 60 min and quenched with 200 µL of ice-cold methanol. Samples were vortex-mixed for 2 min, followed by filtration (Multiscreen, Merck Millipore, Ireland) under vacuum. The filtrate was injected onto a UPLC-MS/MS system.

The hepatic elimination rate constant (k) was calculated directly from the slope of the natural logarithm (percent drug remaining) of the concentration vs. the time profile. In vitro half-life (t_1/2_), intrinsic clearance (CL_int_), and hepatic clearance (CL_int,h_) can be calculated from the equations reported in Figure S3 of the Supporting Information, where [V] is the incubation volume in mL and [P] is the amount of microsomal protein in the incubation mixture.

In vivo *pharmacokinetic study.* For the evaluation of the in vivo pharmacokinetic parameters of **22b**, a pharmacokinetic study was performed in healthy male adult Sprague Dawley rats with in-dwelling jugular catheters weighing 225 ± 25 g. The animals were housed in single occupancy metabolic cages with mesh flooring and urine and feces receptacles. Six animals were dosed with 5 mg/kg intravenously, and the blood samples were collected at the following time points: pre-dose, 0.083, 0.17, 0.33, 0.50, 0.75, 1, 2, 4, 6, 8, and 10 h post-dose. All samples were stored at −80 °C until they were further analyzed. Collected plasma samples were analyzed using a validated UPLC-MS/MS method, and non-compartmental analysis of the plasma concentration data was performed using Phoenix^®^ 6.4.

*Bioanalytical method development and validation.* A sensitive, accurate, and robust quantitative liquid chromatography tandem mass spectrometry (LC-MS/MS, Waters Corporation, Milford, MA, USA) method was developed and validated according to the FDA guidelines. Separation was achieved using a Waters Acquity I-Class liquid chromatography system equipped with a binary pump, a degasser, a temperature-controlled autosampler, and a column oven. The analyte was separated via gradient elution utilizing LC-MS grade water and methanol acidified with 0.02% trifluoroacetic acid (TFA, % *v*/*v*) through a 2.1 × 50 mm ACQUITY UPLC^®^ CSH C18 column with 1.7 μm particle size (PN 186005296, SN 01463729915172). Mass detection was achieved via Waters Xevo TQS Micro triple quadrupole mass spectrometer in positive electrospray ionization (ESI^+^) mode with the multiple reaction monitoring (MRM) function. The method was validated in rat plasma for the linearity range of 1–200 ng/mL. The gradient schematic and fragmentation monitoring parameters are reported in Tables S2 and S3 of the Supporting Information.

## 5. Conclusions

Inspired by original studies combining RF9 and opioid agonists, a lead molecule with an improved pharmacological profile as well as pharmacokinetic properties was generated through a novel DML approach and shown to possess potentially favorable therapeutic properties. This report validates the strategy of rationally designed small molecule DML with activity at both opioid and NPFF receptors, a well-known non-opioid system capable of controlling opioid-mediated responses in vivo [16]. The results of this study could serve as a foundation for further research for novel medications retaining MOR agonism accompanied by NPFF-R antagonism, with the potential to show potent analgesia with minimal or no development of antinociceptive tolerance and therefore less overall liability.

## Figures and Tables

**Table 1:** Binding affinities of **13a–b**, **14a–b**, and **22a–b** at NPFF1-R, NPFF2-R, MOR, DOR, and KOR *.

Receptor Binding, K_i_ (nM) ± SEM							
Cpd.	R_1_	R_2_	NPFF1-R	NPFF2-R	MOR	DOR	KOR
NPFF			1.39 ± 0.08	6.09 ± 1.59	>10,000	>10,000	>10,000
NPAF			99.6 ± 5.0	3.54 ± 0.82	>10,000	>10,000	>10,000
RF9			163 ± 26	158 ± 66	1920 ± 161	>10,000	6710 ± 639
DAMGO					4.15 ± 0.34	857 ± 61	1200 ± 354
SNC80					2761 ± 322	34.6 ± 3.8	2020 ± 617
U69,593					4050 ± 534	6700 ± 739	1.62 ± 0.11
**13a**	3-	3′-OH	2180 ± 280	7080 ± 1260	5.06 ± 0.21	0.481 ± 0.192	2.82 ± 0.39
**13b**	4-	3′-OH	605 ± 136	6020 ± 1150	56.2 ± 7.5	1.67 ± 0.61	30.6 ± 1.1
**14a**	3-	4′-OH	1720 ± 308	>10,000	33.5 ± 4.7	2.33 ± 0.60	32.3 ± 3.5
**14b**	4-	4′-OH	1980 ± 288	4825 ± 1010	313 ± 11	6.46 ± 1.11	56.6 ± 3.8
**22a**	3-	H	1400 ± 61	2570 ± 423	6.45 ± 1.23	3.37 ± 0.59	91.0 ± 9.9
**22b**	4-	H	929 ± 183	272 ± 1	428 ± 33	11.4 ± 4.3	270 ± 43
** 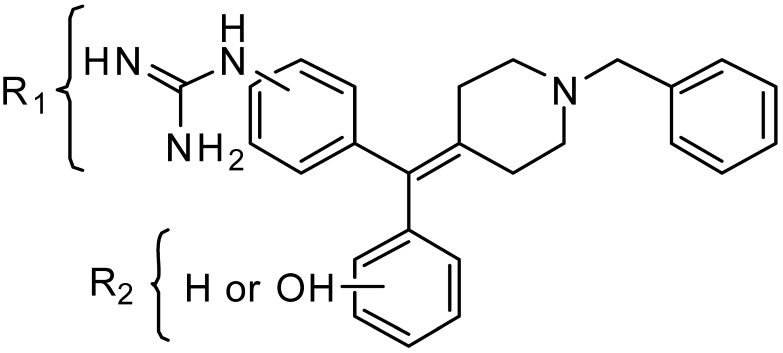 **							

* K_i_ values, expressed as mean ± S.E.M., were determined at NPFF1-R, NPFF2-R, MOR, DOR, and KOR using [^3^H]NPVF, [^3^H]EYF, [^3^H]DAMGO, [^3^H]DPDPE, and [^3^H]U-69,593, respectively.

**Table 2:** Functional agonistic activities of **13a–b**, **14a–b**, and **22a–b** in the [^35^S]GTPγS assay at MOR, DOR, and KOR *.

Functional Activity (Agonist Mode) (nM)								
			[^35^S]GTPγS MOR		[^35^S]GTPγS DOR		[^35^S]GTPγS KOR	
Cpd.	R_1_	R_2_	EC_50_ ± SEM	Type	EC_50_ ± SEM	Type	EC_50_ ± SEM	Type
DAMGO			27.7 ± 2.9	C				
DPDPE					16.3 ± 1.9	C		
U69,593							6.07 ± 0.65	C
**13a**	3-	3′-OH	28.3 ± 2.5	F	24.1 ± 2.5	F	41.8 ± 4.9	P
**13b**	4-	3′-OH	1442 ± 226	P	9.62 ±1.27	F	267.9 ± 31.9	P
**14a**	3-	4′-OH	419.8 ± 54.5	P	455.0 ± 61.7	F	481.9 ± 81.6	P
**14b**	4-	4′-OH	n/a	A	227.0 ± 12.5	F	135.8 ± 23.7	P
**22a**	3-	H	101.9 ± 21.3	P	255.9 ± 24.9	F	254.7 ± 57.7	P
**22b**	4-	H	576.2 ± 311.3	P	539.4 ± 93.2	F	2711 ± 398	P
** 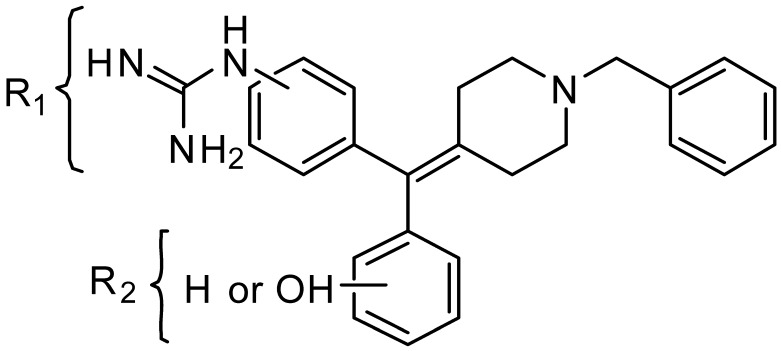 **								

* EC_50_ values were determined at MOR, DOR, and KOR using DAMGO, DPDPE, and U-69,593, respectively. C = control agonist; F = full agonist; P = partial agonist; A = antagonist.

**Table 3:** Pharmacokinetic parameters of **22b** following intravenous administration (5 mg/kg) in Sprague Dawley rats *.

Parameters	
C_0_ (ng/mL)	9152.0 ± 3736.3
AUC/dose_0_inf_ (ng × h/mL/mg)	3004.7 ± 108.1
CL (L/h)	0.3 ± 0.0
k (1/h)	0.2 ± 0.0
T_1/2_ (h)	4.1 ± 0.5
V_d_ (L)	2.0 ± 0.2

C_0_: initial concentration; V_d_: apparent volume of distribution; AUC/dose: total exposure normalized by dose; CL: clearance; k: elimination rate constant; T_1/2_: elimination half-life. * Data are shown as mean (*n* = 6) ± SEM.

## Data Availability

Data are contained within the article and the Supplementary Material.

## Supplementary Materials

The following supporting information can be downloaded at https://www.mdpi.com/article/10.3390/molecules30132851/s1, supplemental data (chemistry mass spectra, in vitro pharmacological assessments, and functional activities of **13a–b**, **14a–b**, and **22a–b**, Figures S1 and S2 and Table S1; Pharmacokinetics Figures S3–S5, Tables S2 and S3, and additional references [17,46]).

## Abbreviations

ANOVA: analysis of variance; CI: confidence interval; CLAMS: Comprehensive Lab Animal Monitoring System; CPA: conditioned place aversion; CPP: conditioned place preference; DAMGO: [D-Ala_2_,N-MePhe_4_,glyol]enkephalin; DMSO: dimethyl sulfoxide; DOR: delta opioid receptor; DPDPE: [D-Pen_2_,D-Pen_5_]enkephalin; i.c.v.: intracerebroventricular; i.p.: intraperitoneal; KOR: kappa opioid receptor; MOR: mu opioid receptor; MS: morphine sulfate; NPFF: neuropeptide FF; RM: repeated measures. Amino acids are the L-isomer unless otherwise specified; abbreviations for standard amino acids follow IUPAC-IUB Joint Commission of Biochemical Nomenclature.
