# Supplementary material for: Dual Opioid–Neuropeptide FF Small Molecule Ligands Demonstrate Analgesia with Reduced Tolerance Liabilities
Source: Molecules. 2025 Jul 3;30(13):2851. doi: 10.3390/molecules30132851 (PMC12251305; doi:10.3390/molecules30132851)
Supplement: Supplementary file 1 [file molecules-30-02851-s001.zip › molecules-3717547-supplementary.pdf]

Dual Opioid-Neuropeptide FF Small Molecule Ligands Demonstrate Analgesia with Reduced Tolerance Liabilities

Supporting information

Table of content

Chemistry

NMR spectra of 13a……………………………………………………………………..3

LCMS traces of 13a……………………………………………………………………..5

NMR spectra of 13b……………………………………………………………………..6

LCMS traces of 13b……………………………………………………………………..8

NMR spectra of 14a……………………………………………………………………..9

LCMS traces of 14a……………………………………………………………………11

NMR spectra of 14b……………………………………………………………………12

LCMS traces of 14b……………………………………………………………………14

NMR spectra of 22a……………………………………………………………………15

LCMS traces of 22a……………………………………………………………………17

NMR spectra of 22b……………………………………………………………………18

LCMS traces of 22b……………………………………………………………………20

LCMS traces of blank…………………………………………………………………21

***In vitro* pharmacology.**

Figure S1. Radioligand competition binding concentration-response curves of **13a-b, 14a-b,** and **22a-b** when tested against MOR, KOR, DOR, NPFF1-R and NPFF2-R………………………**22**

Figure S2. Concentration-response curves assessing functional agonist activities of **13a-b, 14a-b,** and **22a-b** when tested against MOR (left), KOR (middle) and DOR (right) in the [^{35}S]GTPγS assay ……………………………………………………………………………………**23**

Table S1. Functional Activities of **13a-b, 14a-b**, and **22a-b**……………………………………**24**

Pharmacokinetics.

Figure S3. Rat Liver microsomal stability of compound **22b**……………………………………..**25**

Figure S4. Equations used for the calculation of i*n-vitro* half-life ($t_{1/2}$), intrinsic clearance (CL_{int}) and hepatic clearance ($CL_{int,h}$) ……………………………………………………………**25**

Table S2. Gradient elution conditions…………………..…………………………………**25**

Table S3. MS/MS parameters…………………..…………………..………..……………**26**

Figure S5. Plasma concentration-time profile of **22b** following intravenous administration (5 mg/kg) in *Sprague Dawley* rats (Mean, SEM) ……………………………………………..**26**

References…….……………………………………………………………………………..**27**

Chemistry. ^{1}H NMR spectrum of 13a

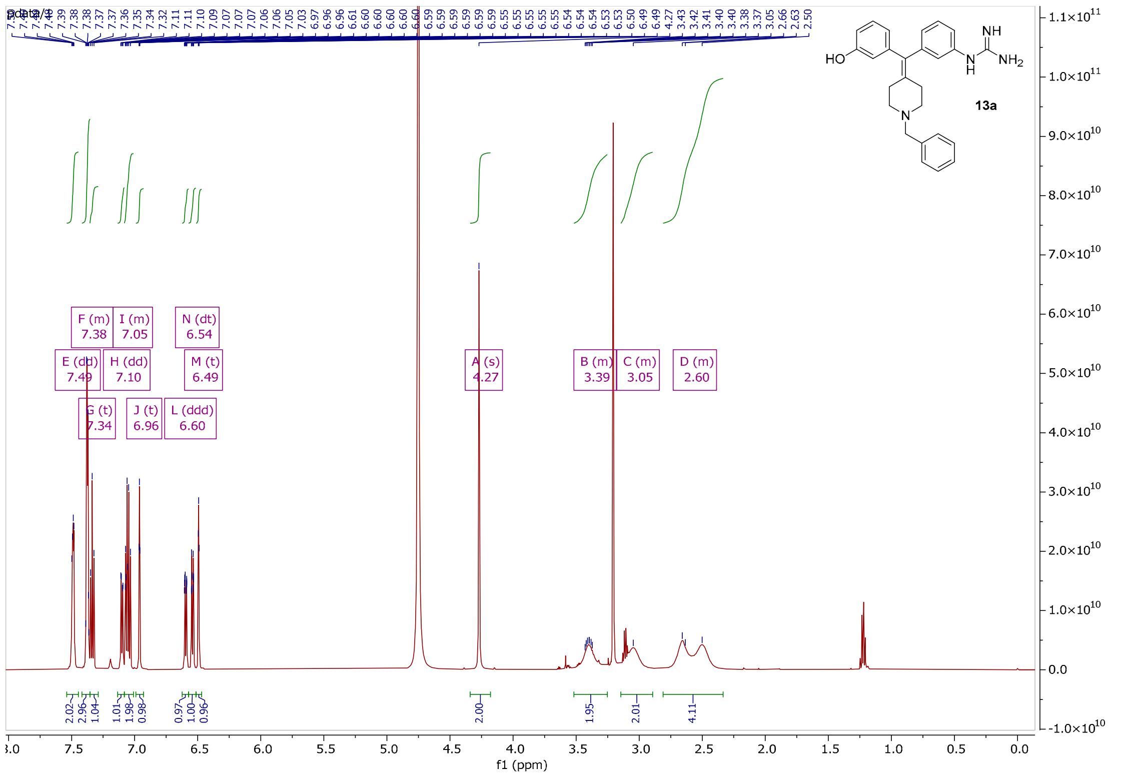

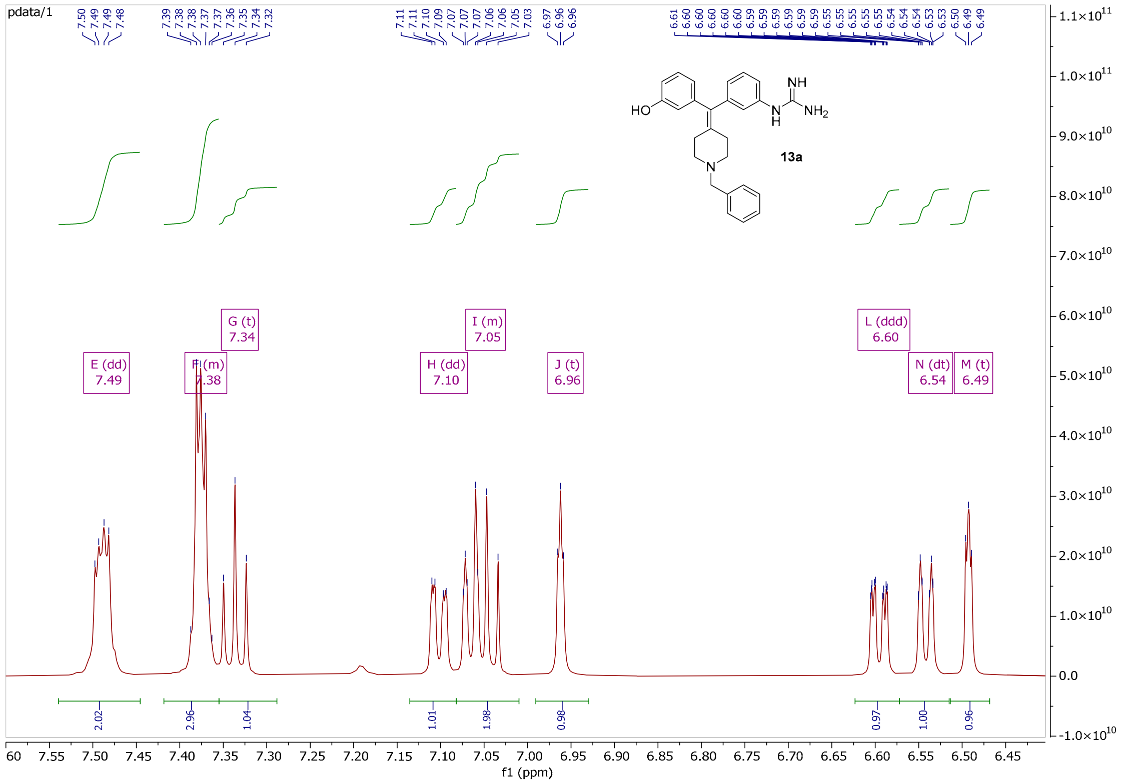

^{13}C NMR spectrum of 13a

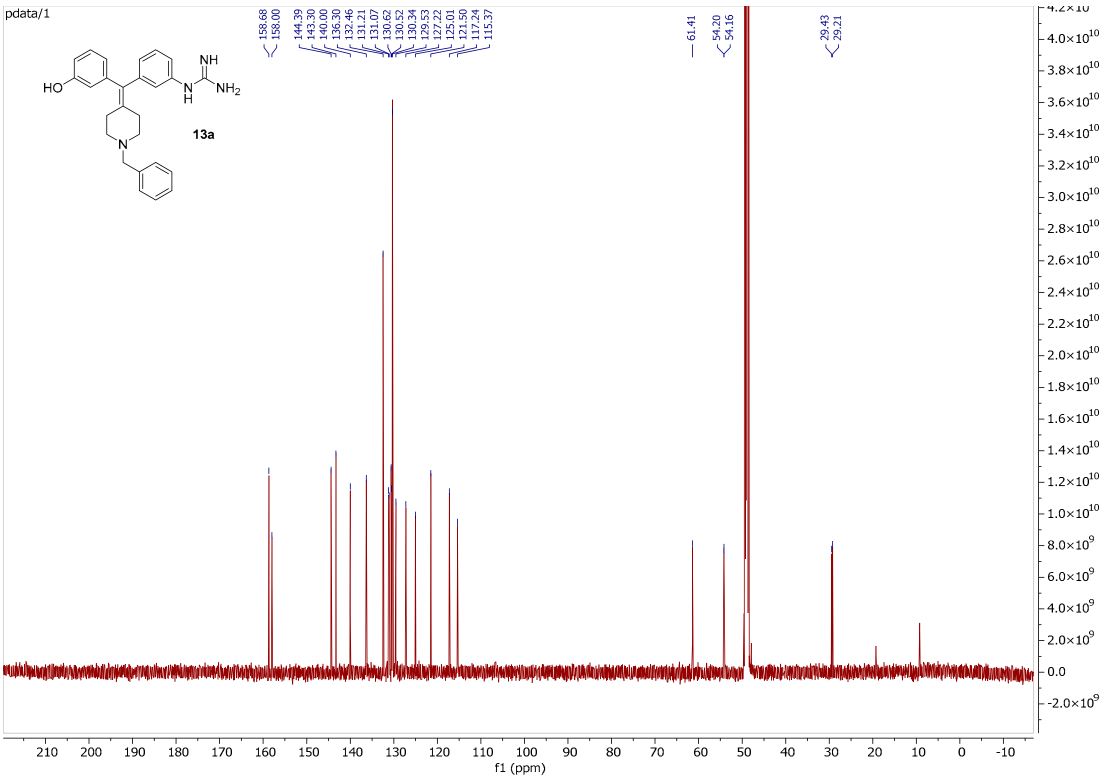

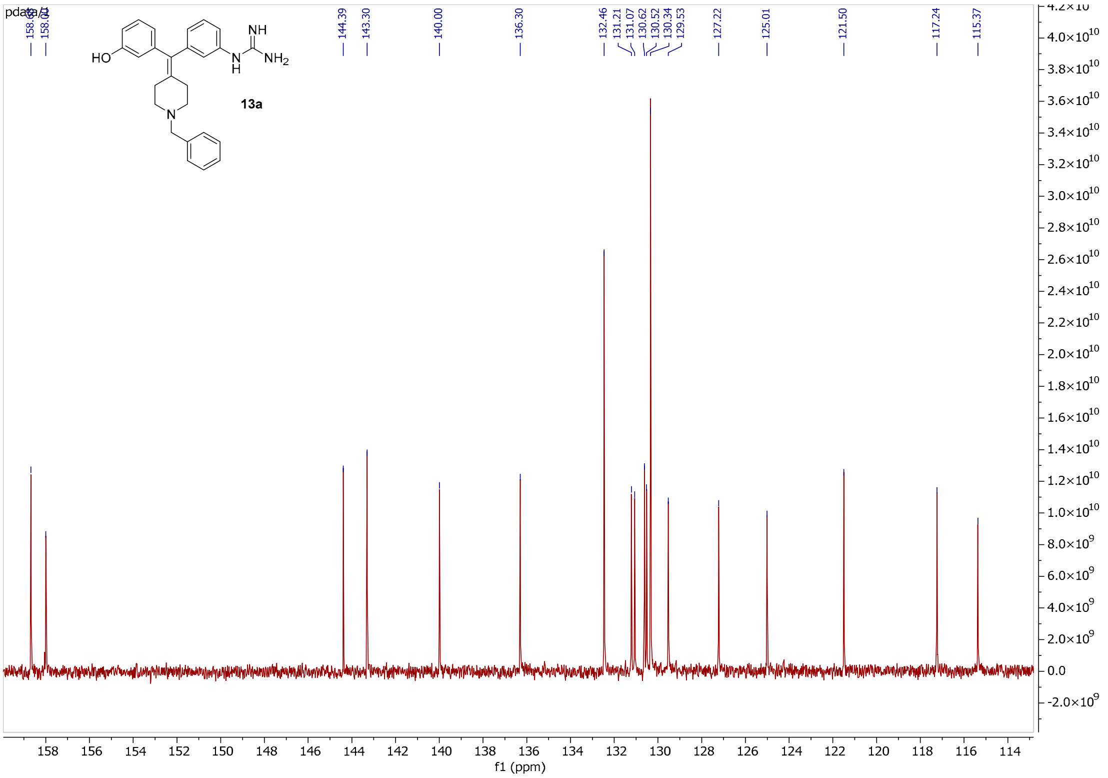

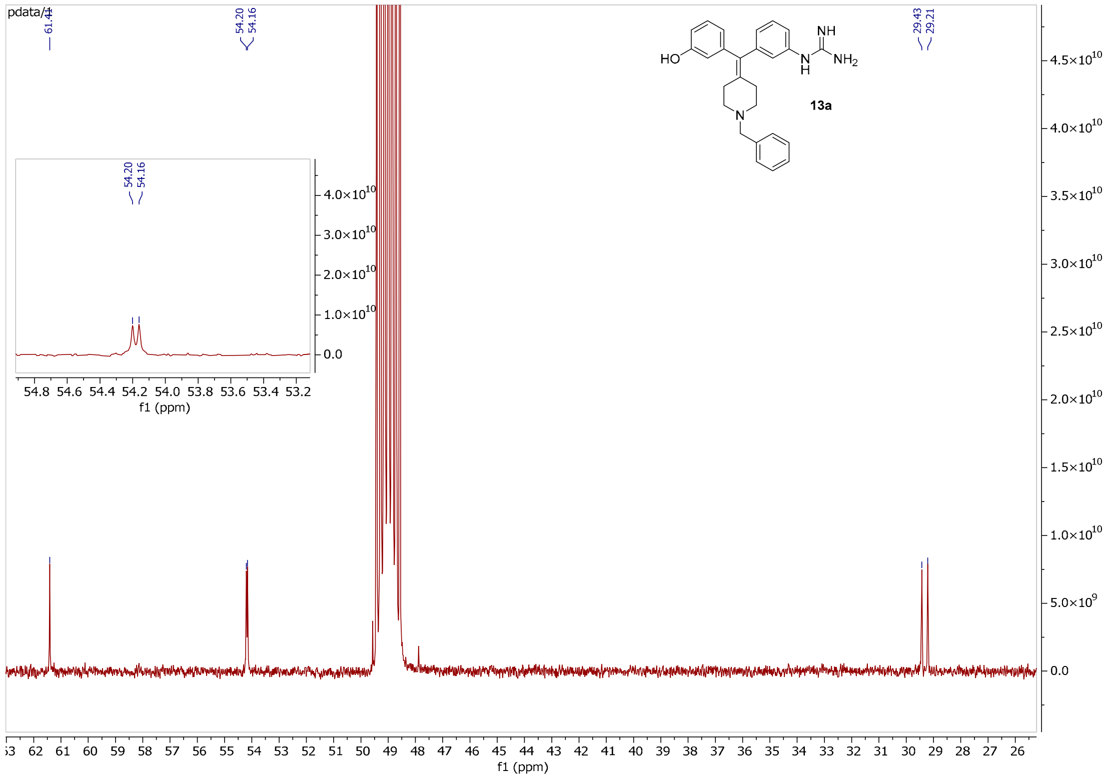

LCMS traces of 13a

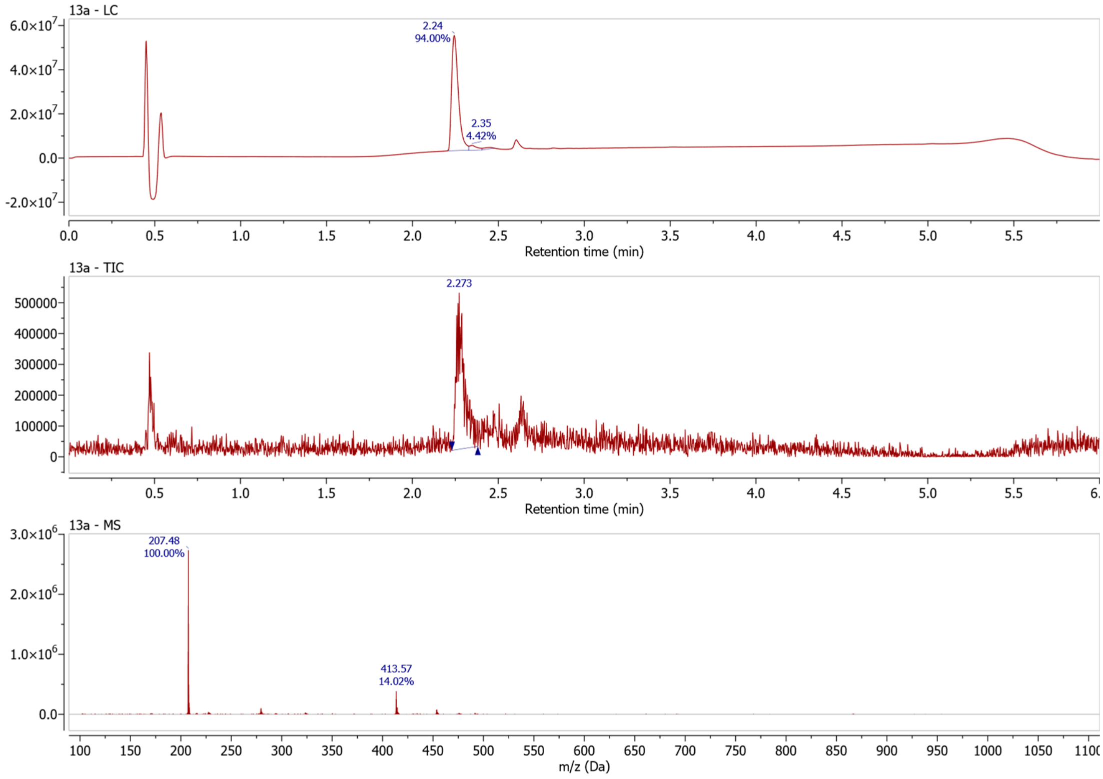

^{1}H NMR spectrum of 13b

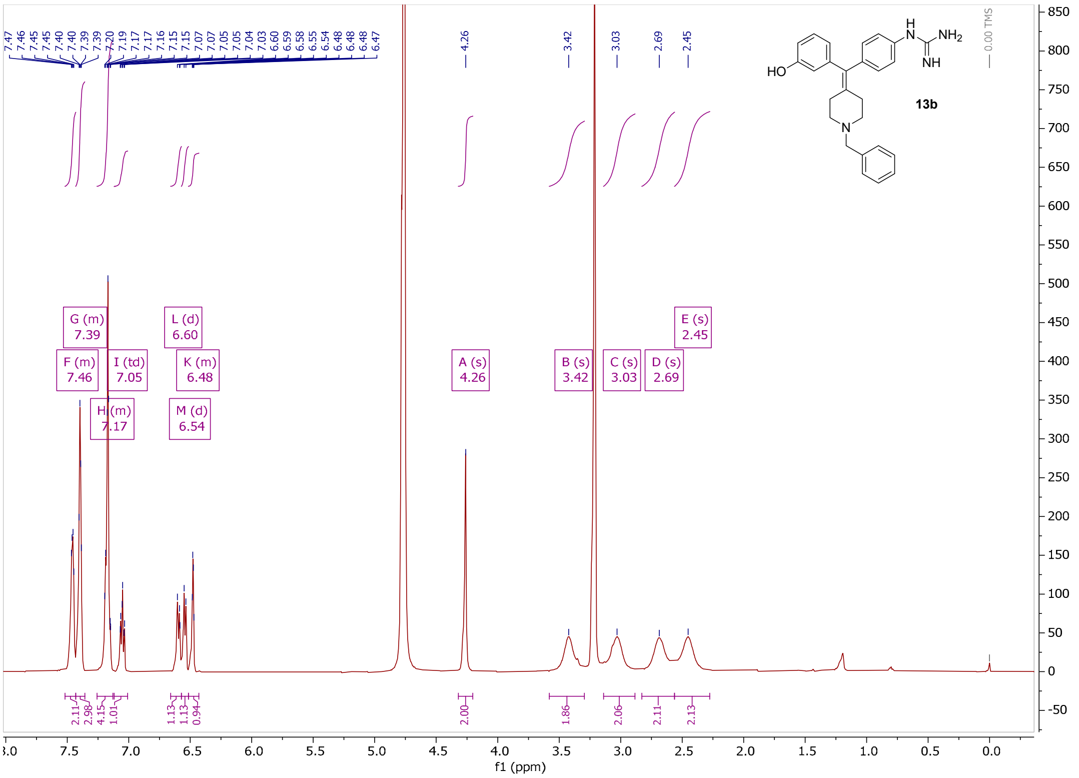

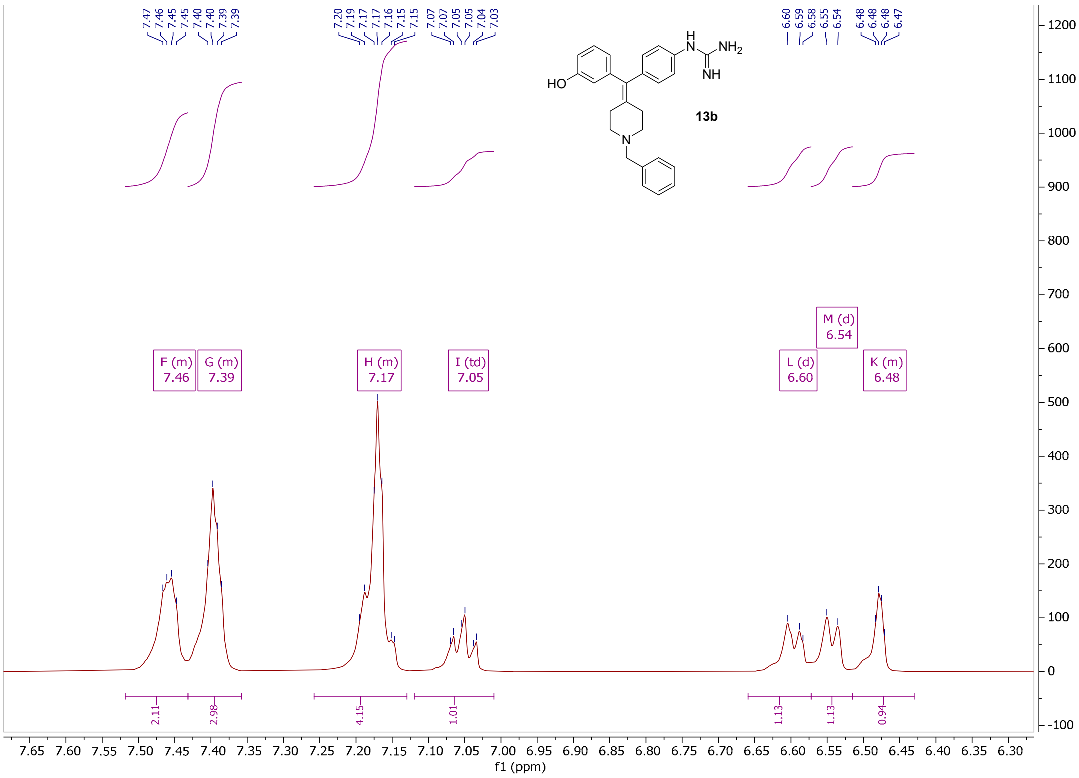

^{13}C NMR spectrum of 13b

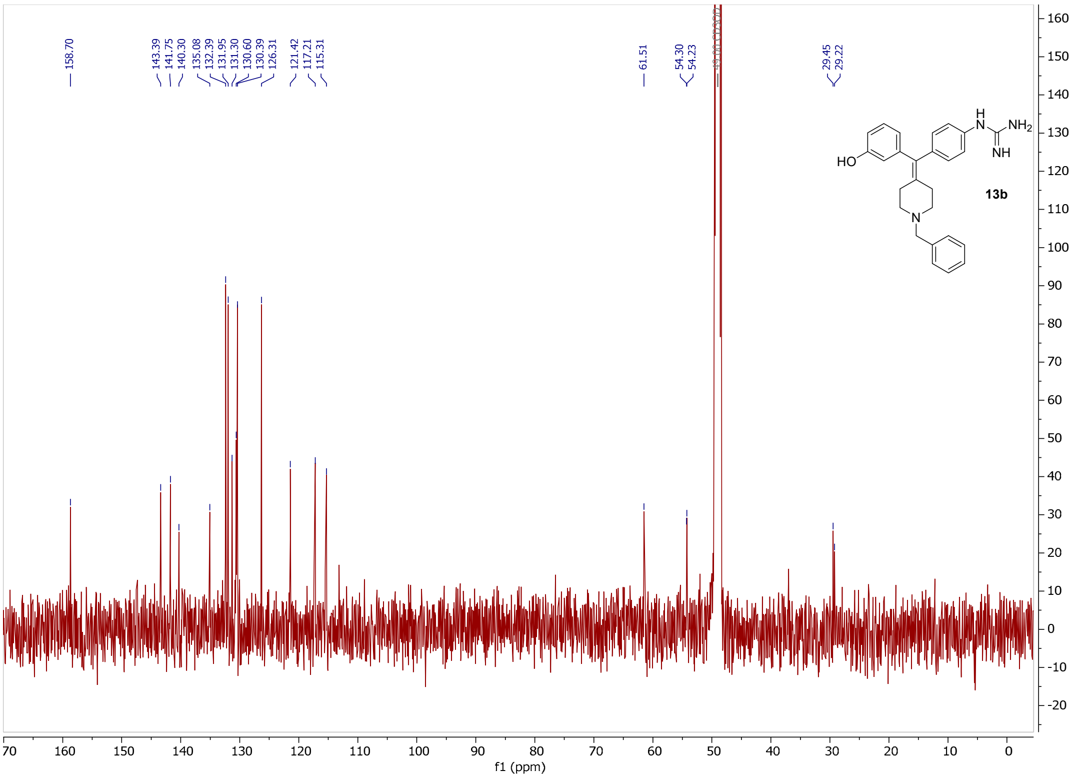

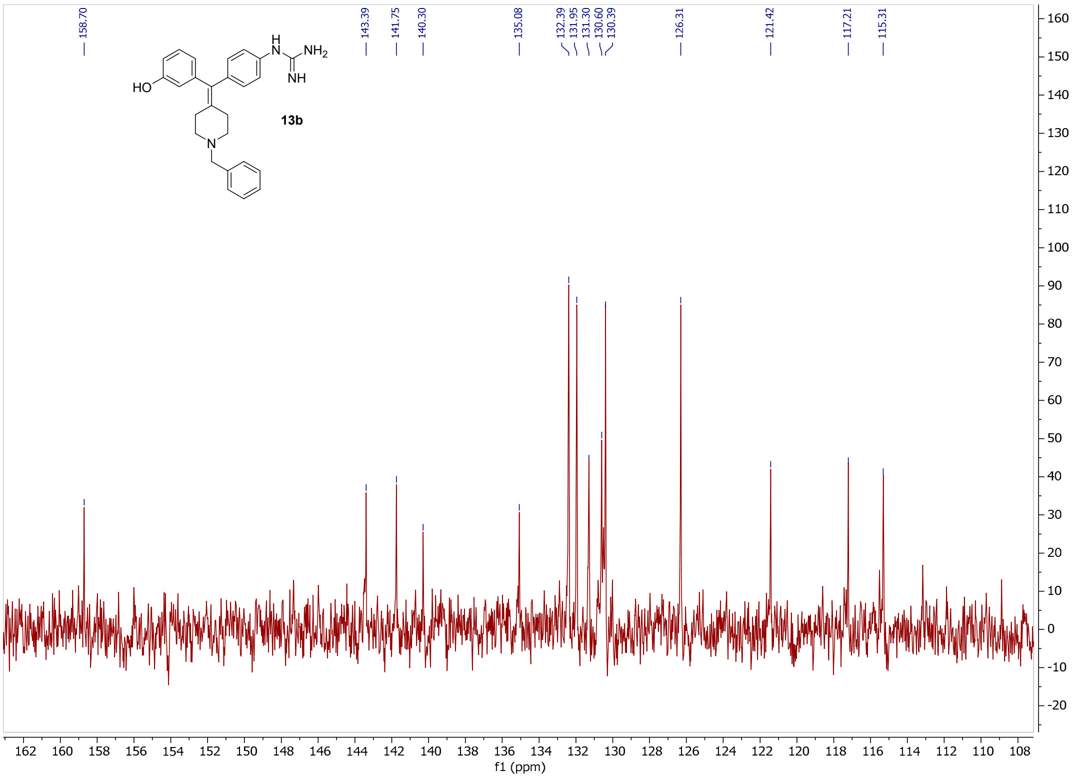

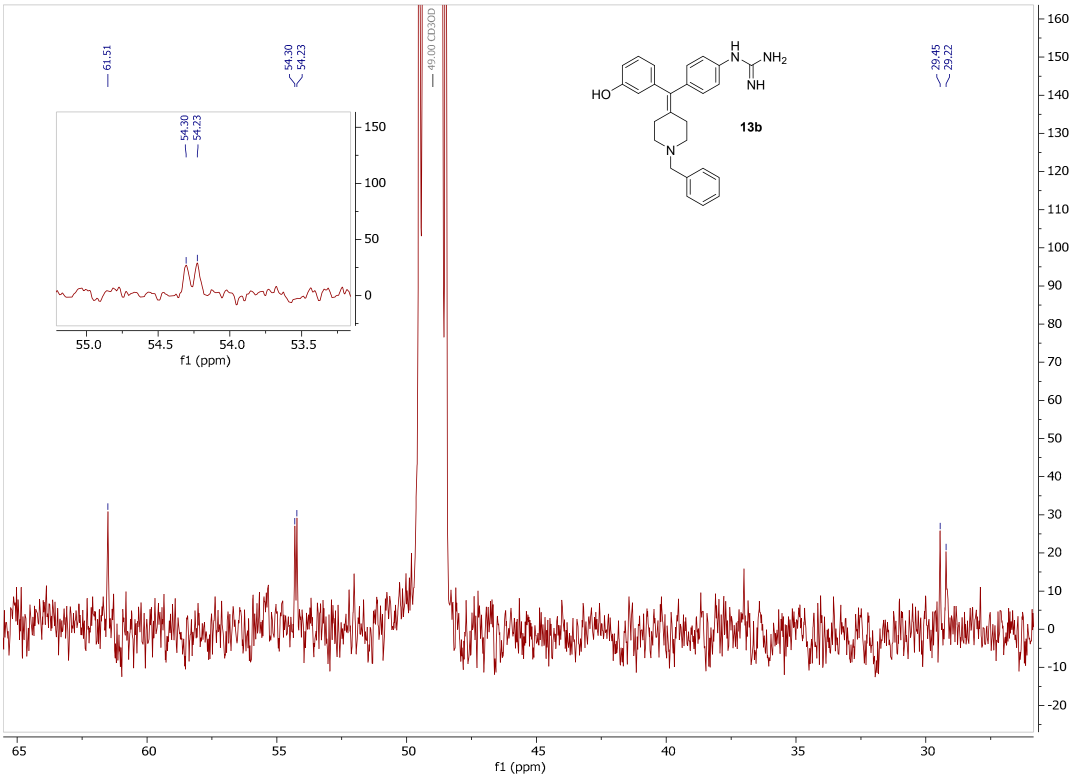

LCMS traces of 13b

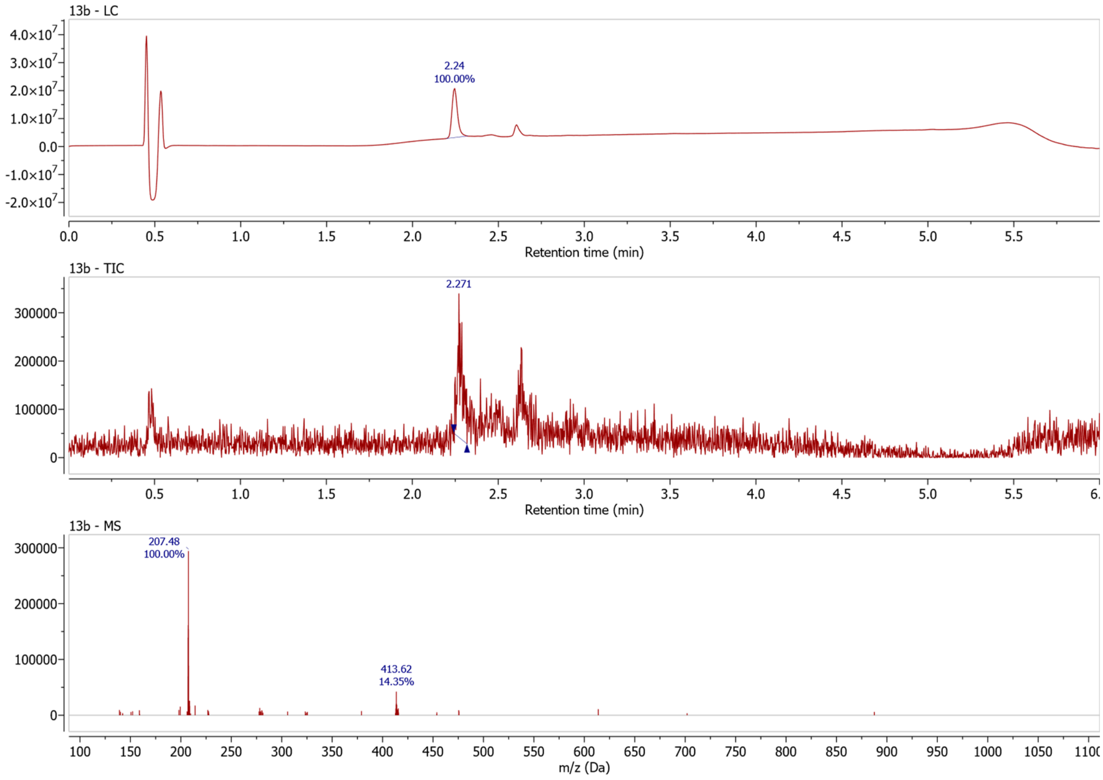

^{1}H NMR spectrum of 14a

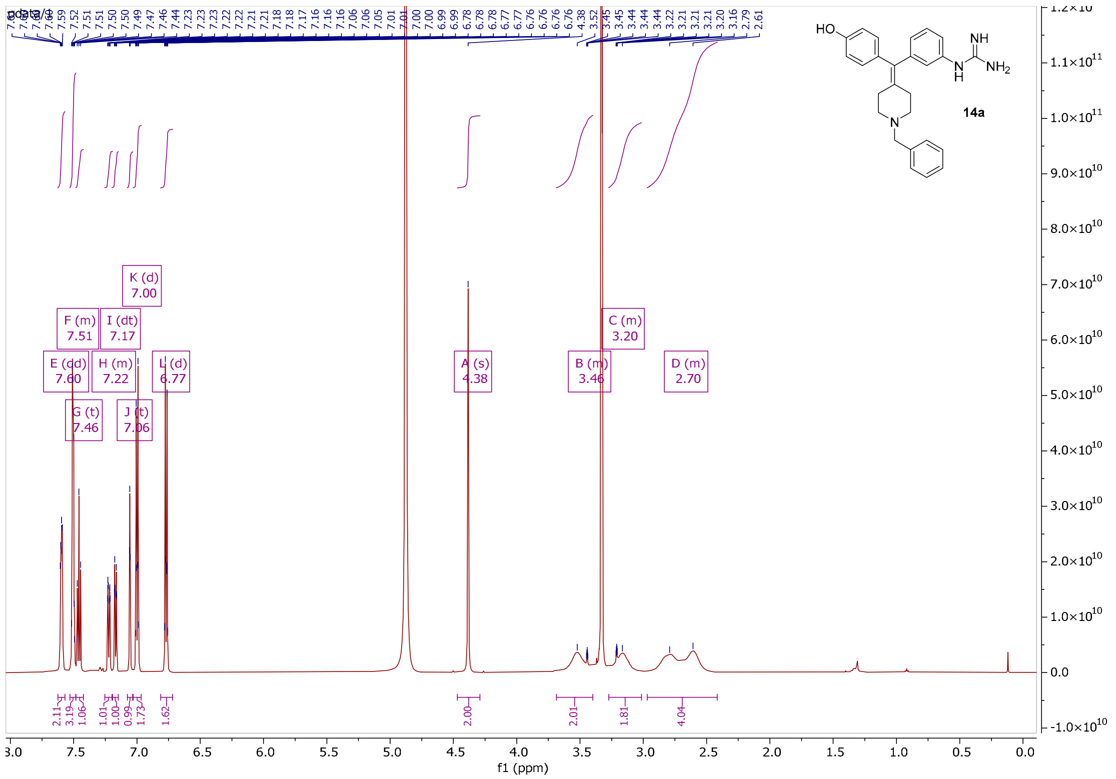

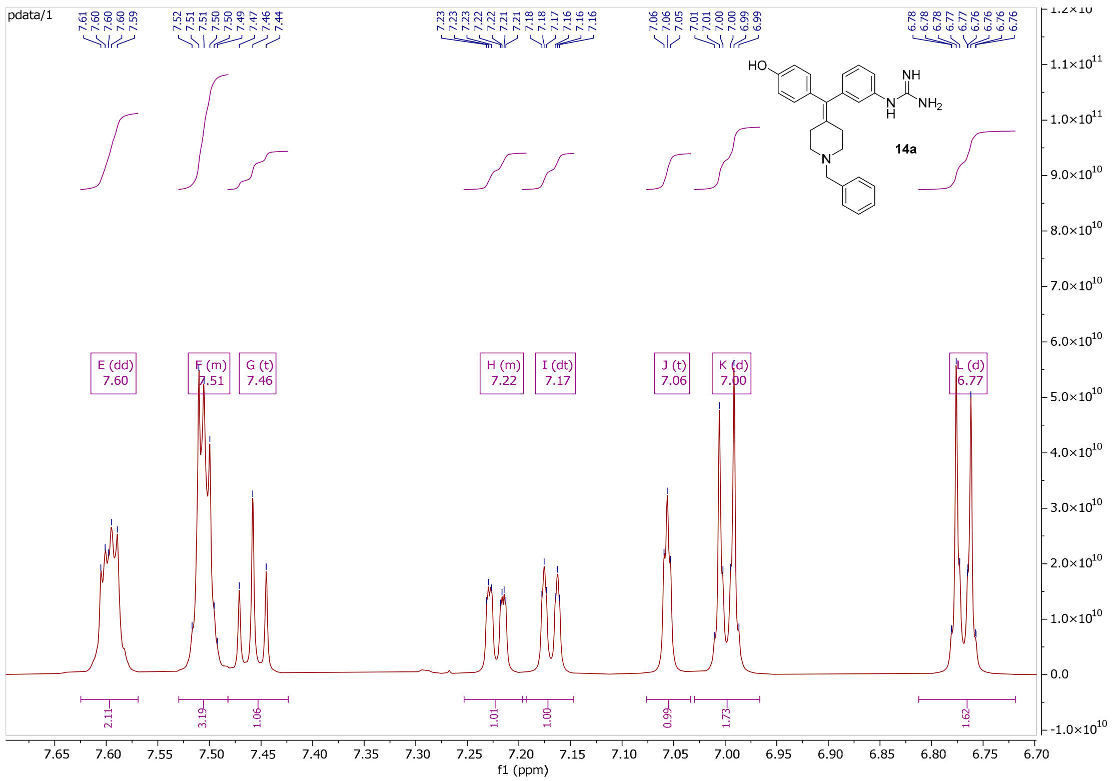

^{13}C NMR spectrum of 14a

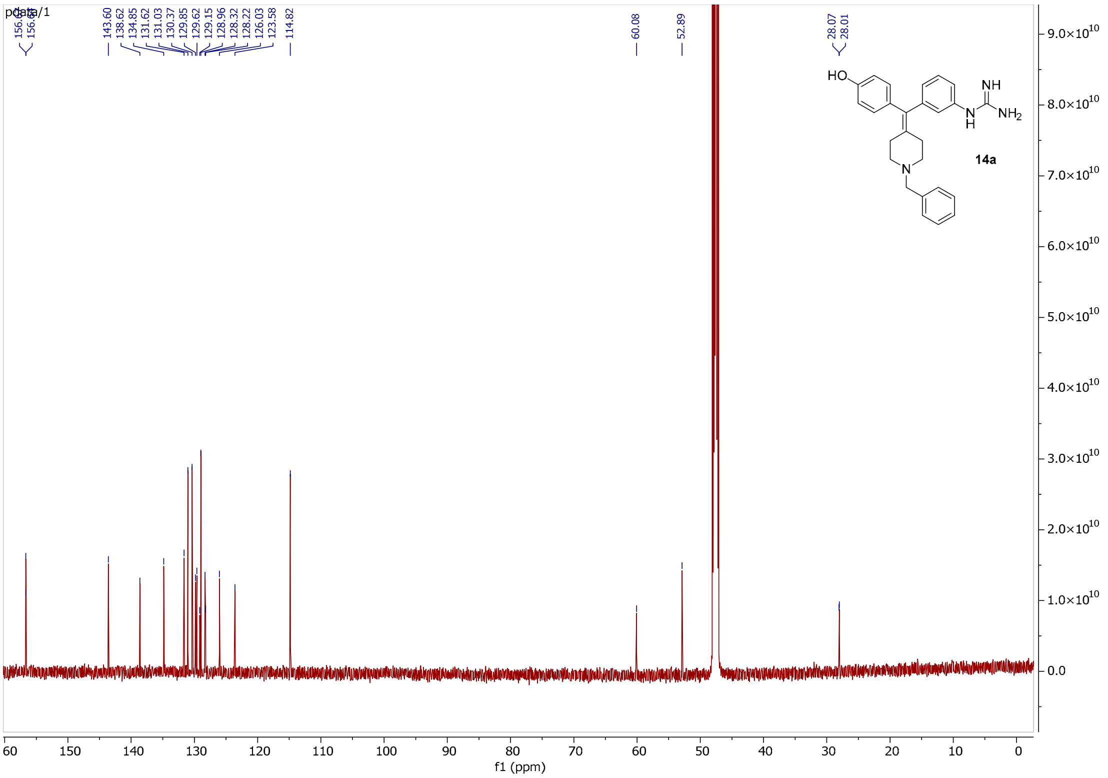

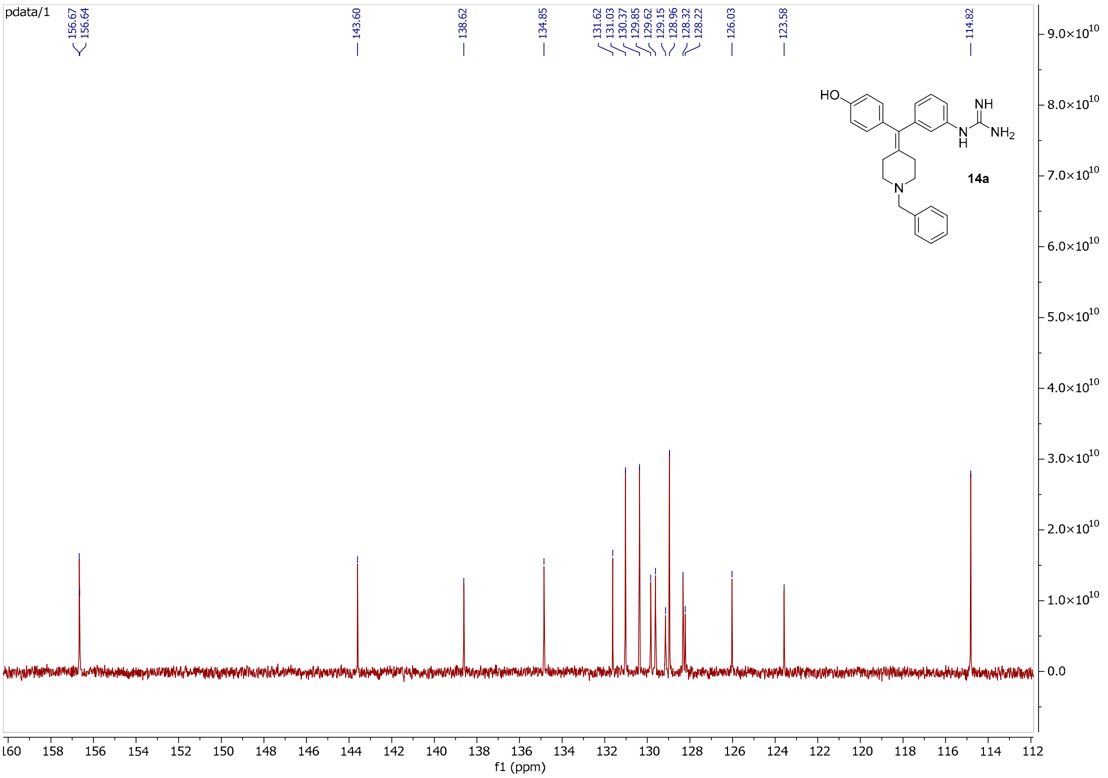

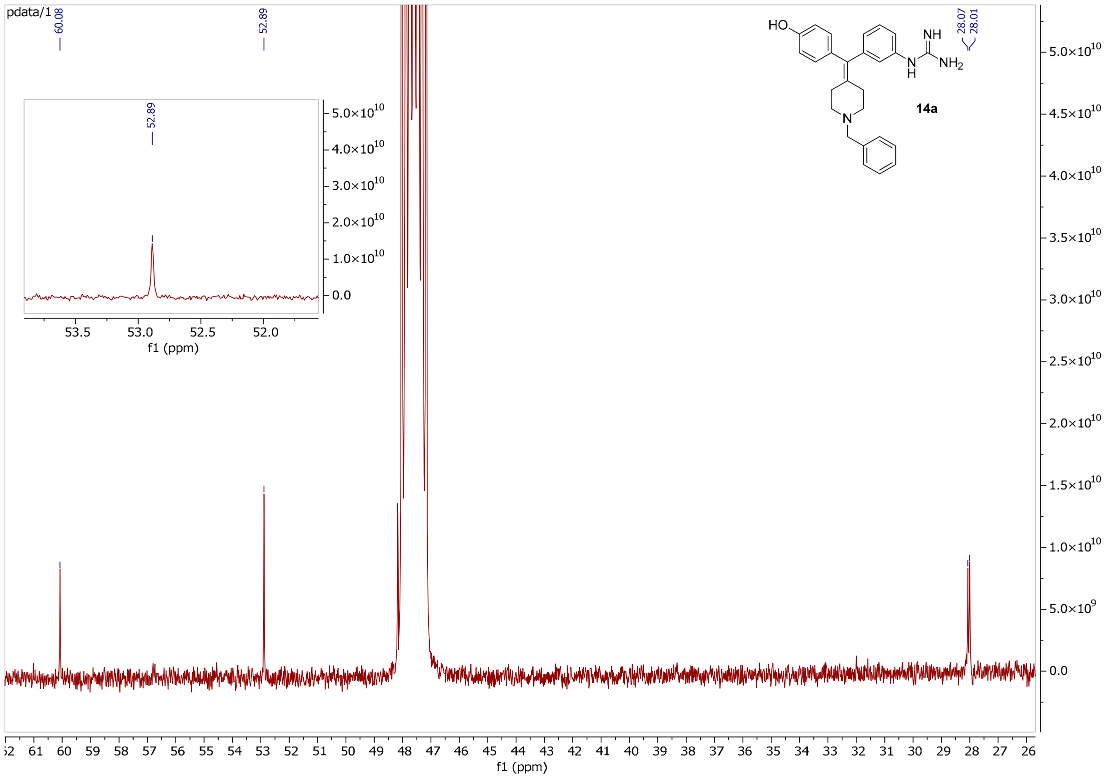

LCMS traces of 14a

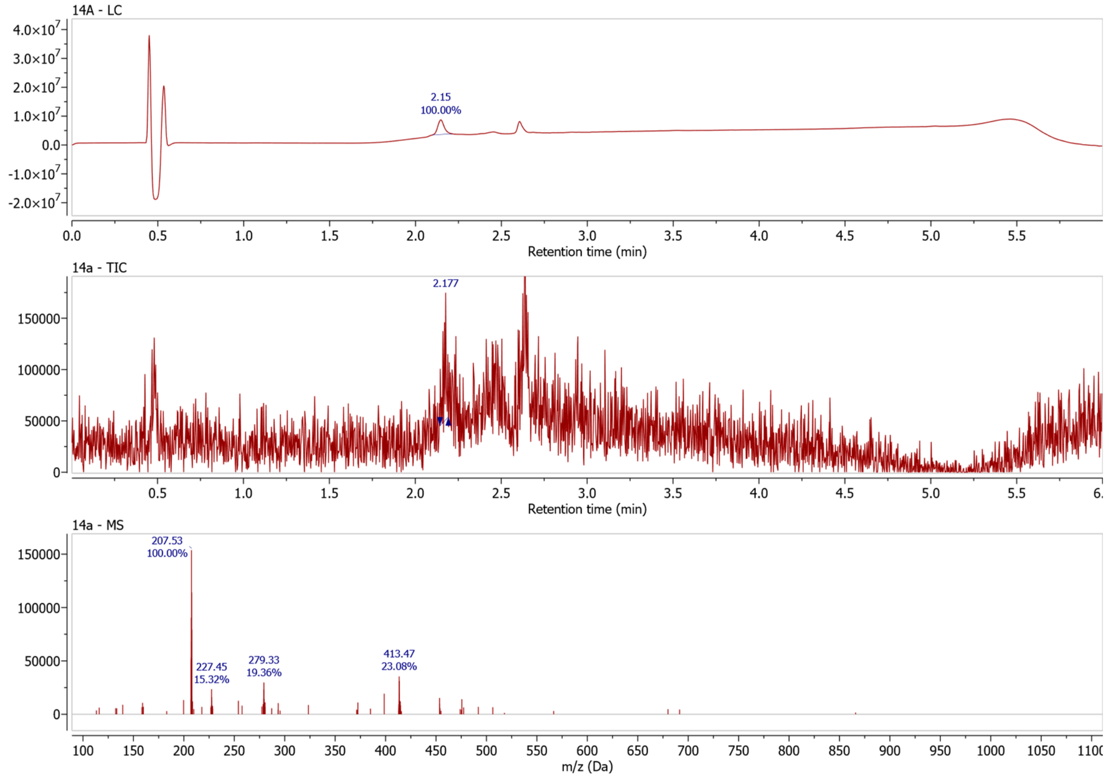

^{1}H NMR spectrum of 14b

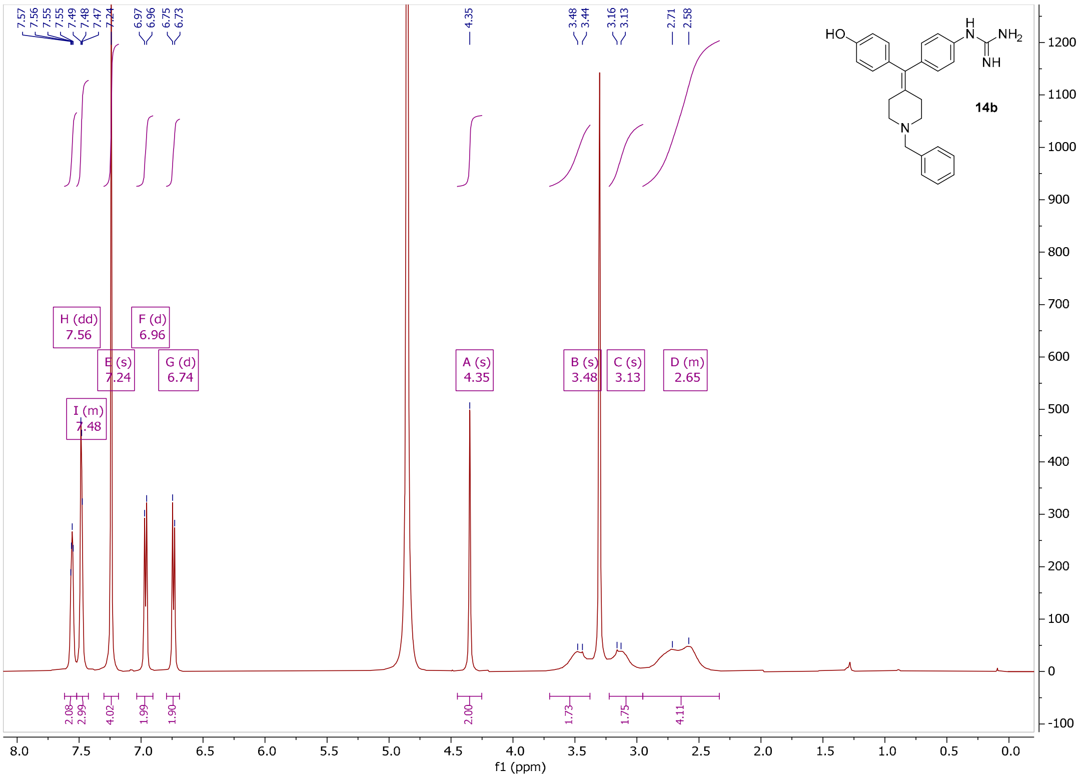

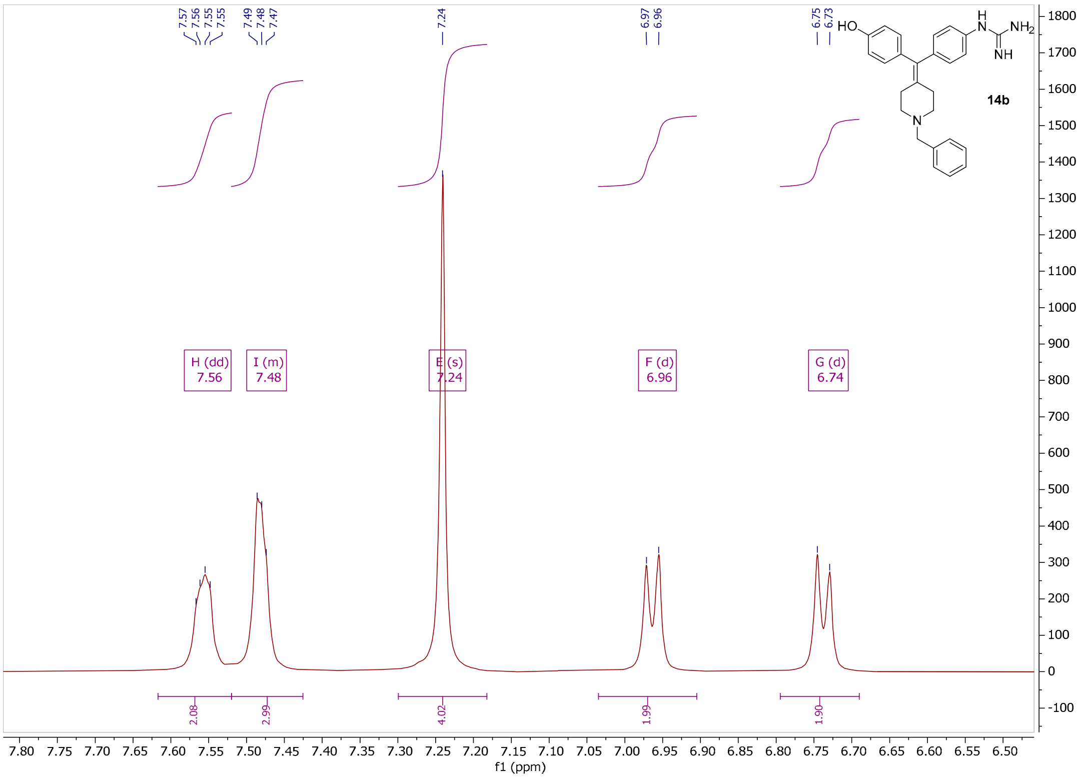

^{13}C NMR spectrum of 14b

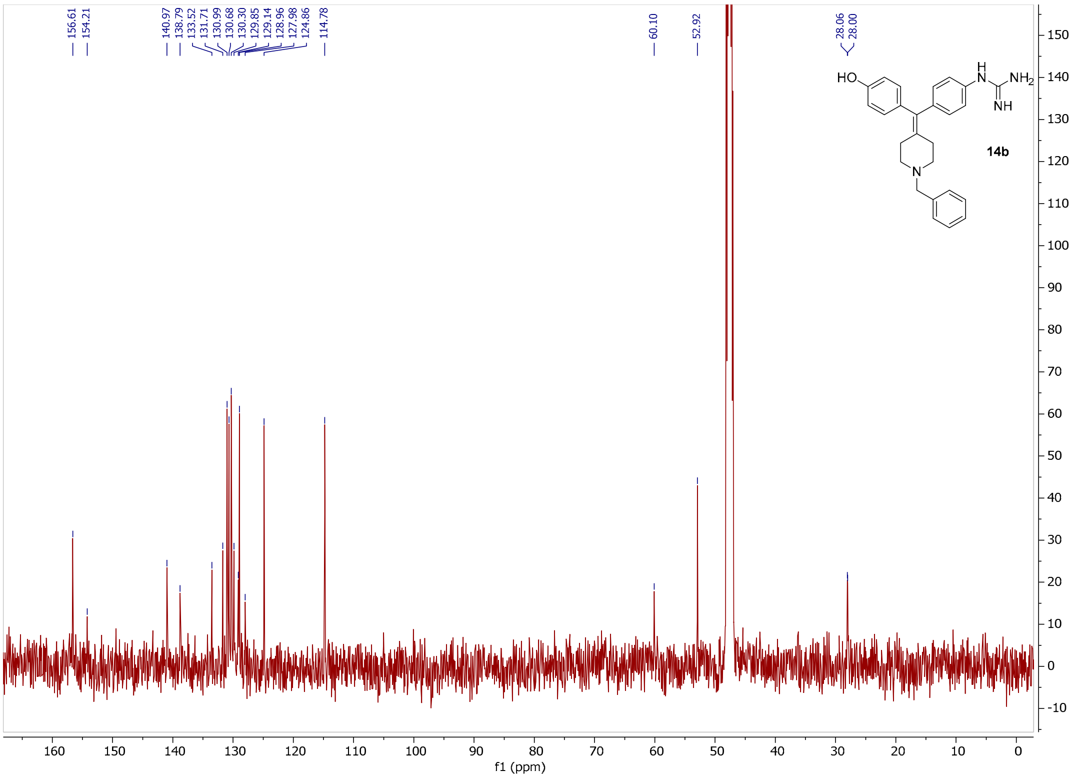

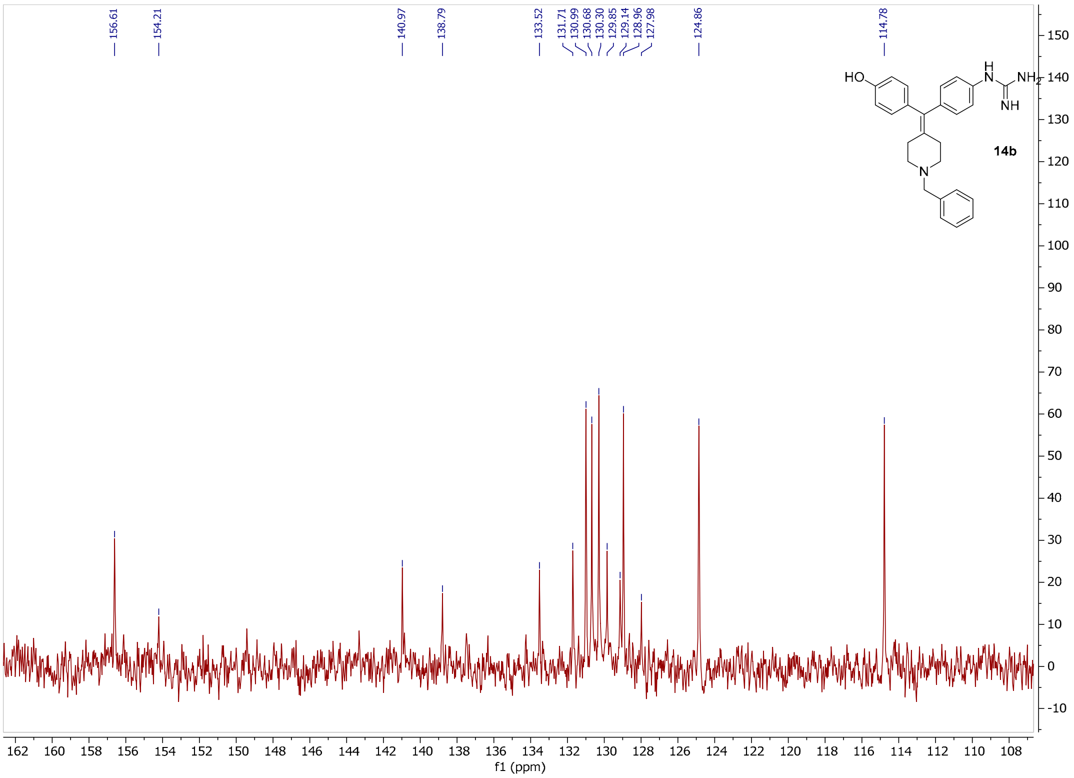

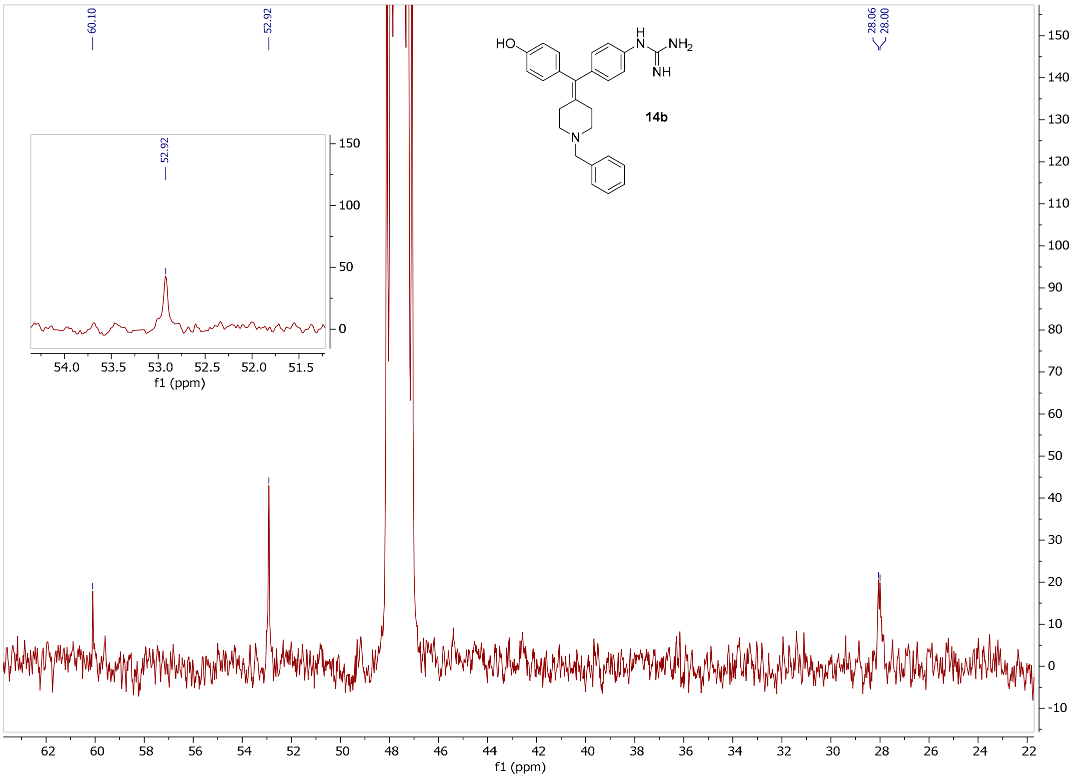

LCMS traces of 14b

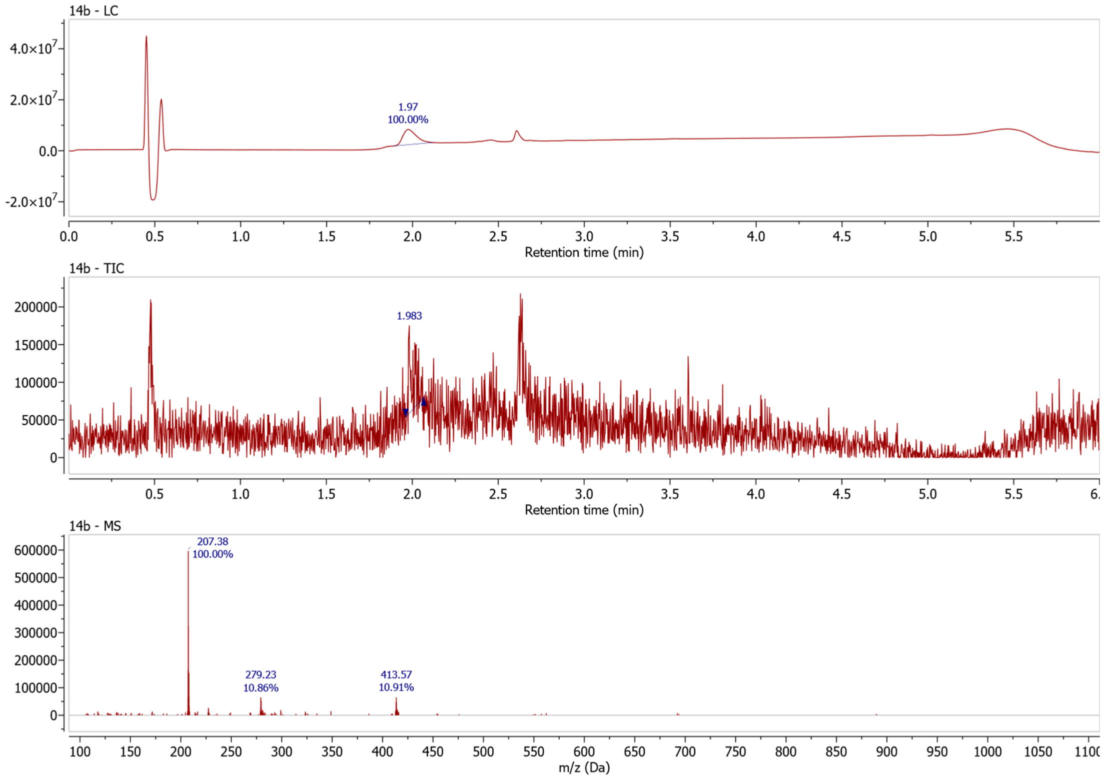

^{1}H NMR spectrum of 22a

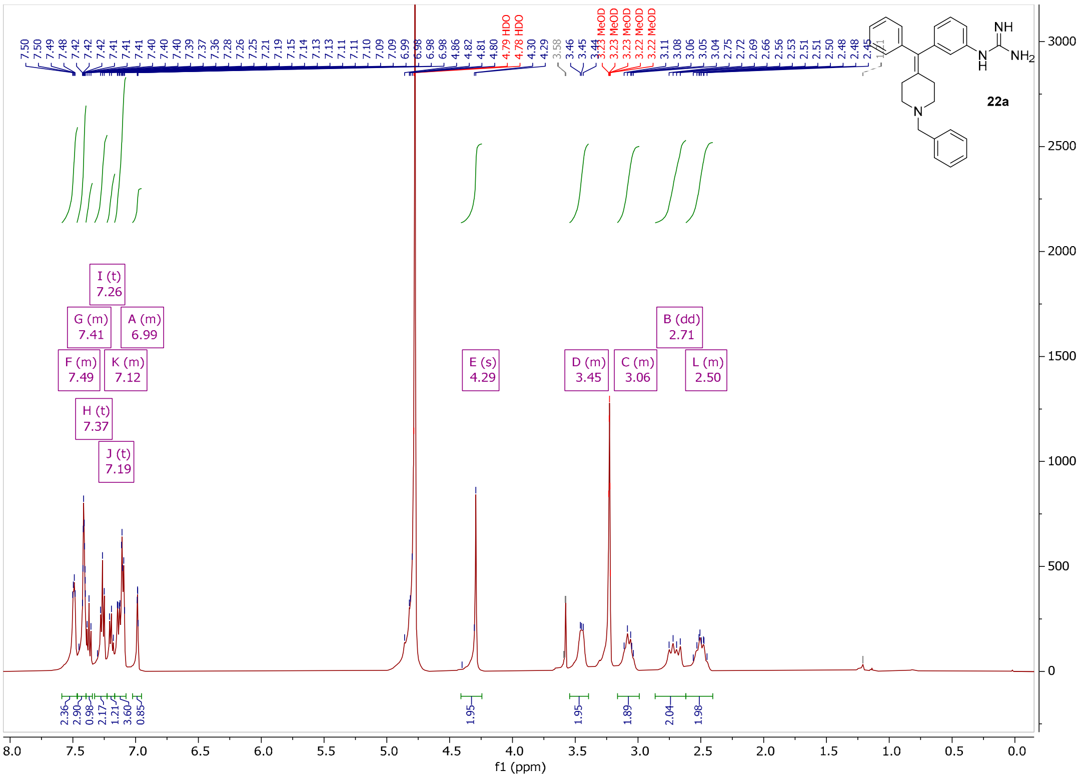

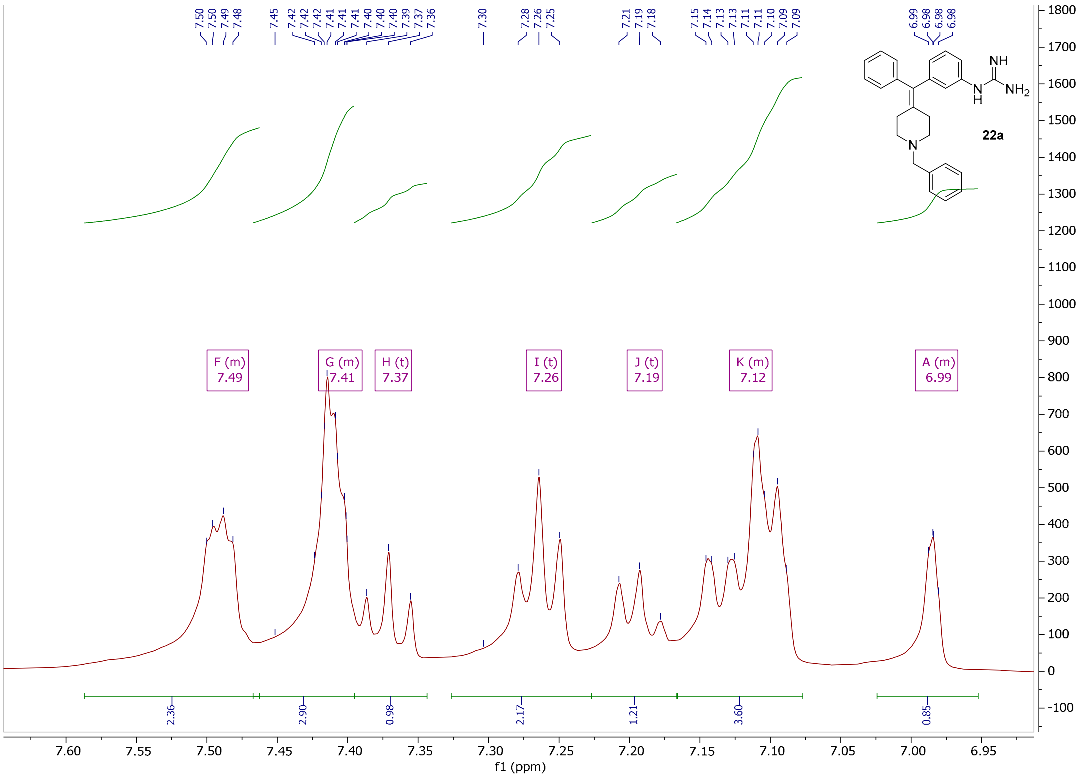

^{13}C NMR spectrum of 22a

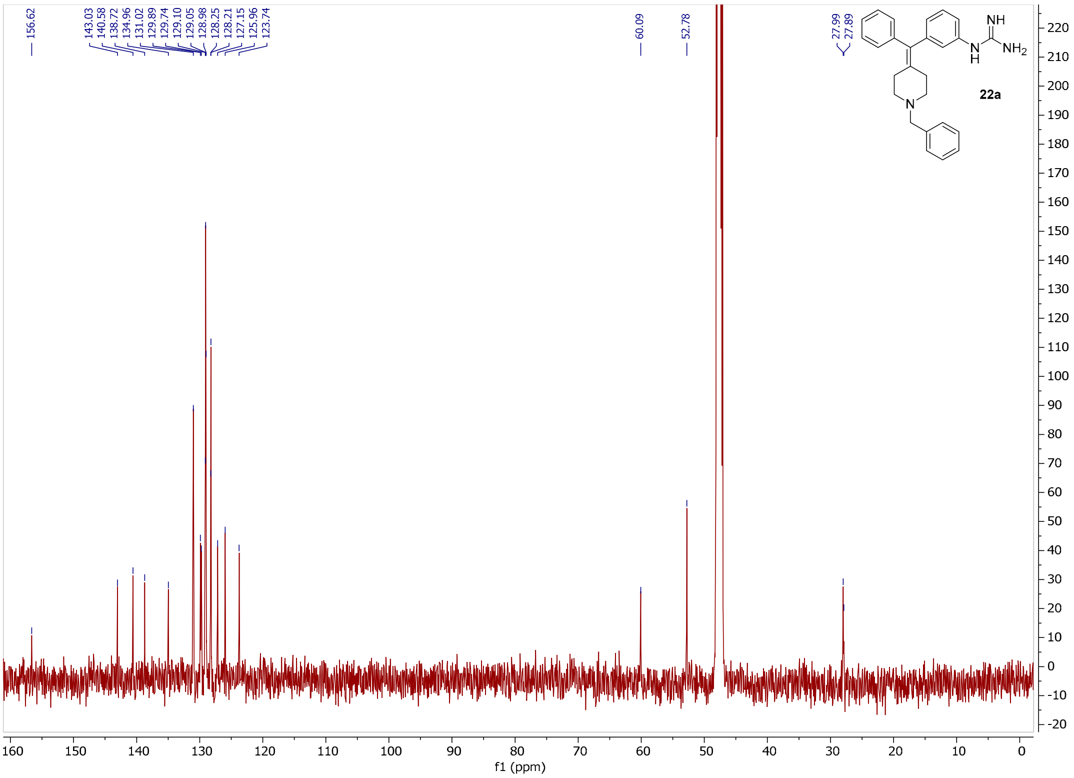

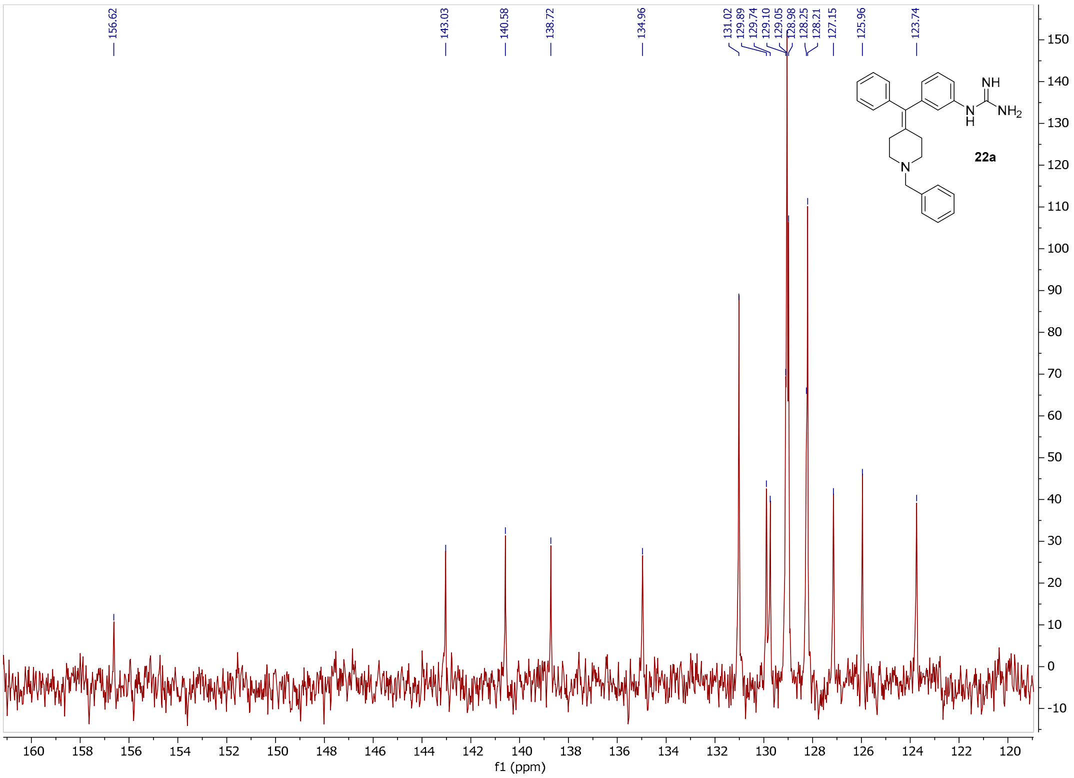

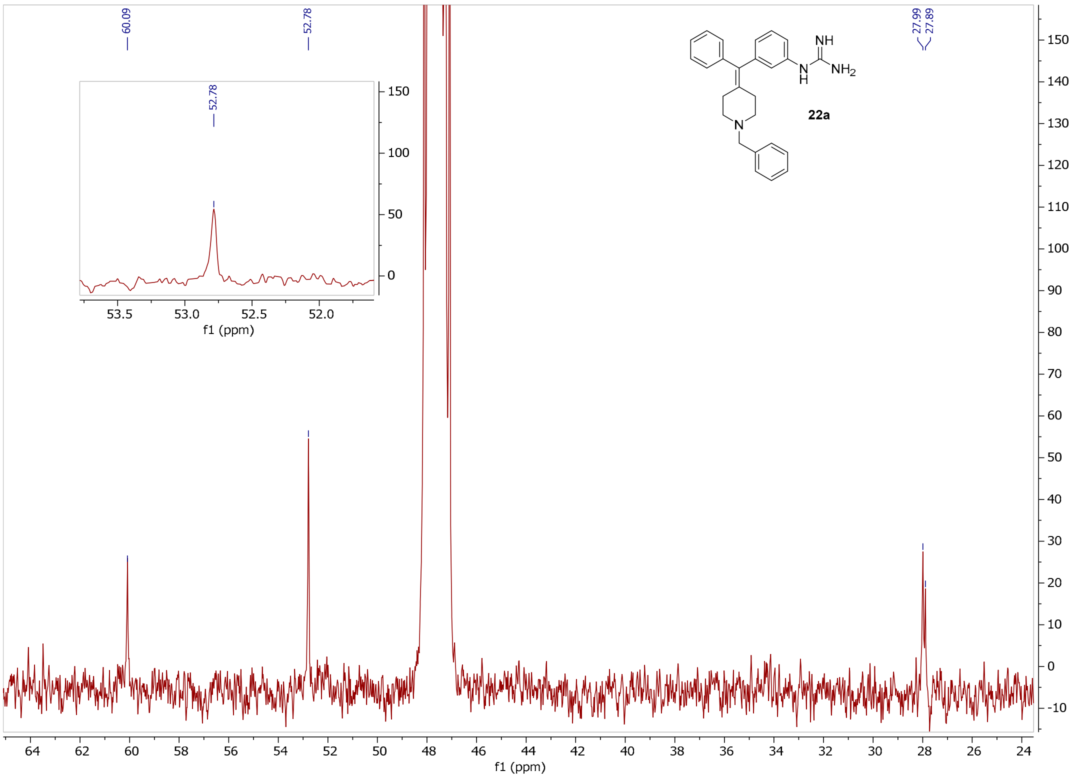

LCMS traces of 22a

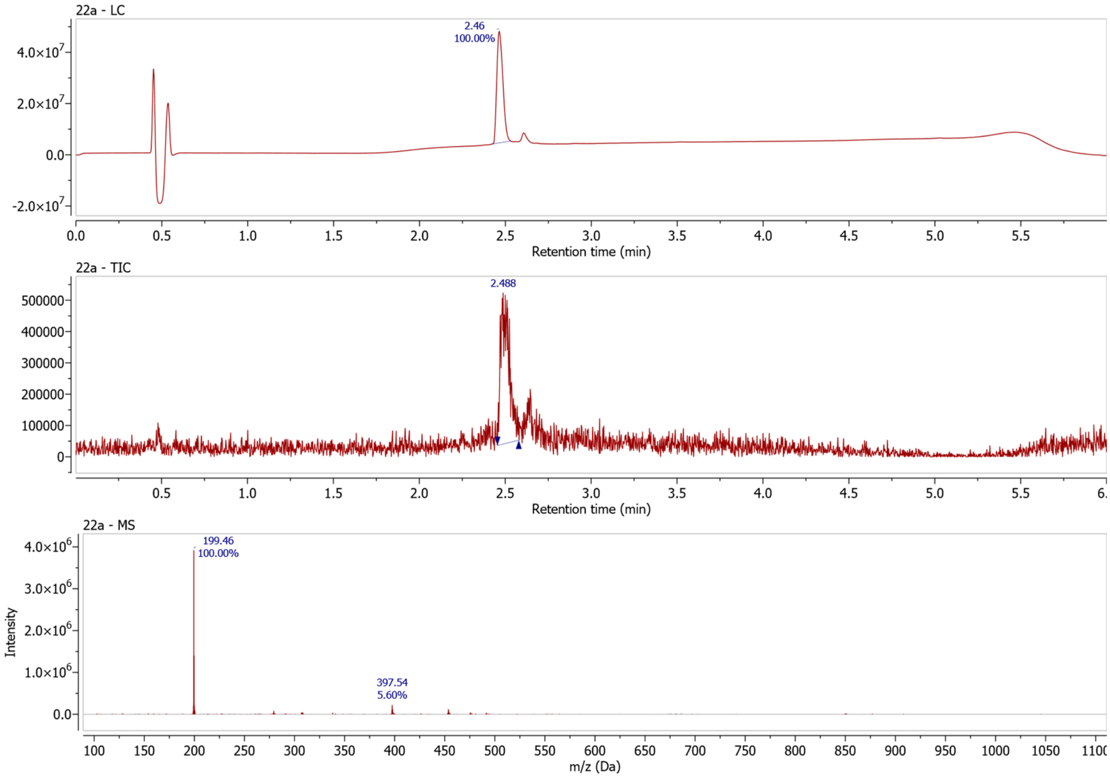

^{1}H NMR spectrum of 22b

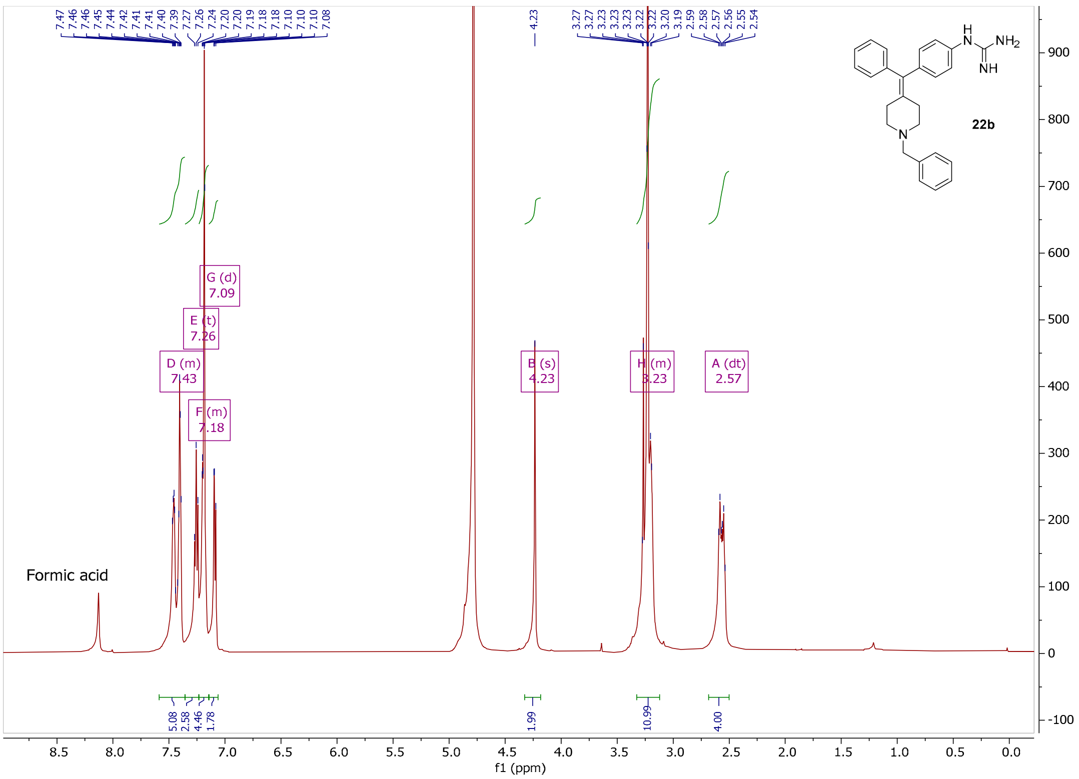

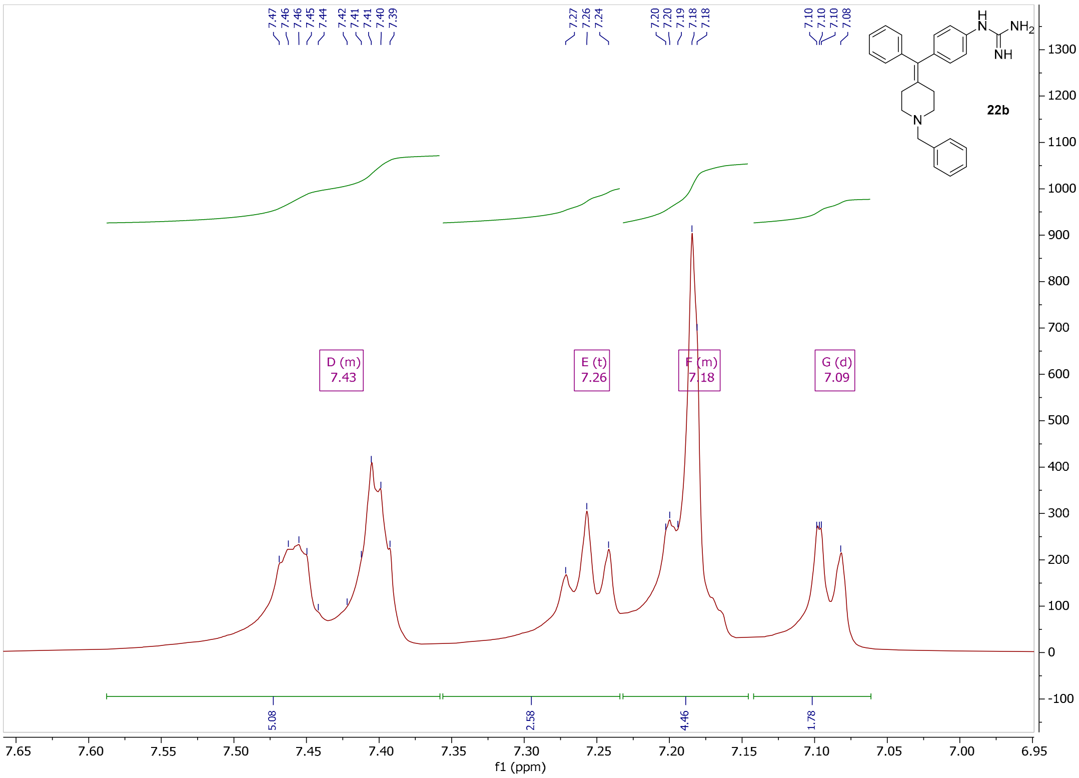

^{13}C NMR spectrum of 22b

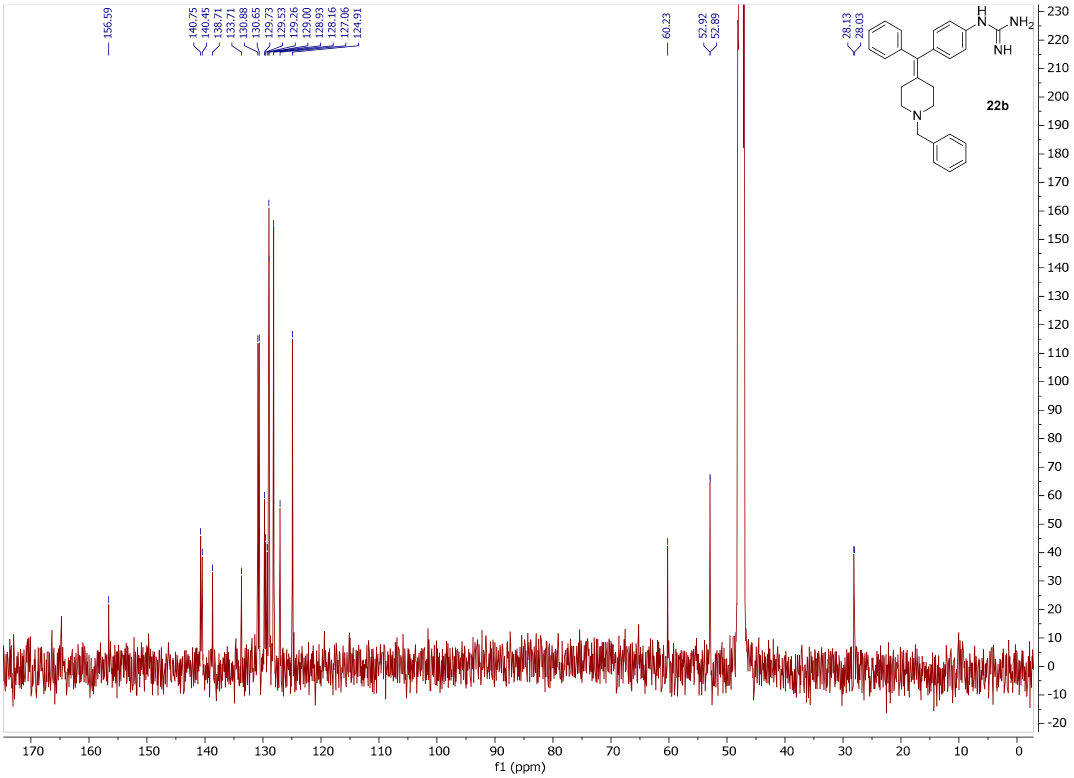

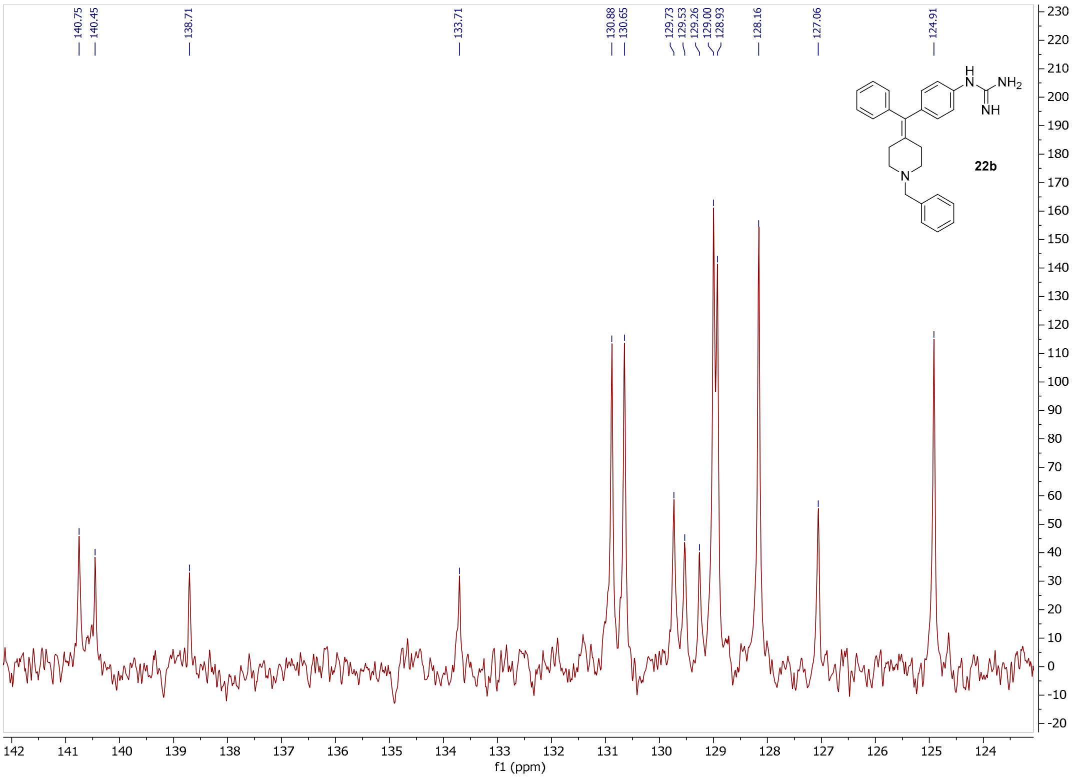

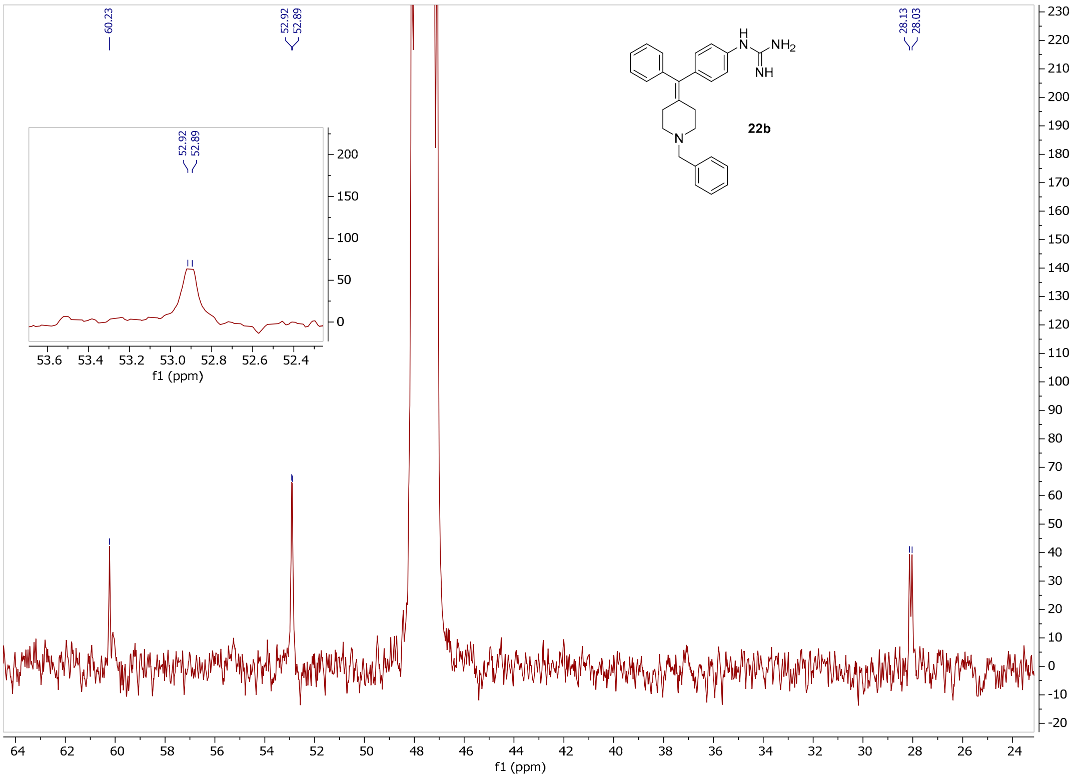

LCMS traces of 22b

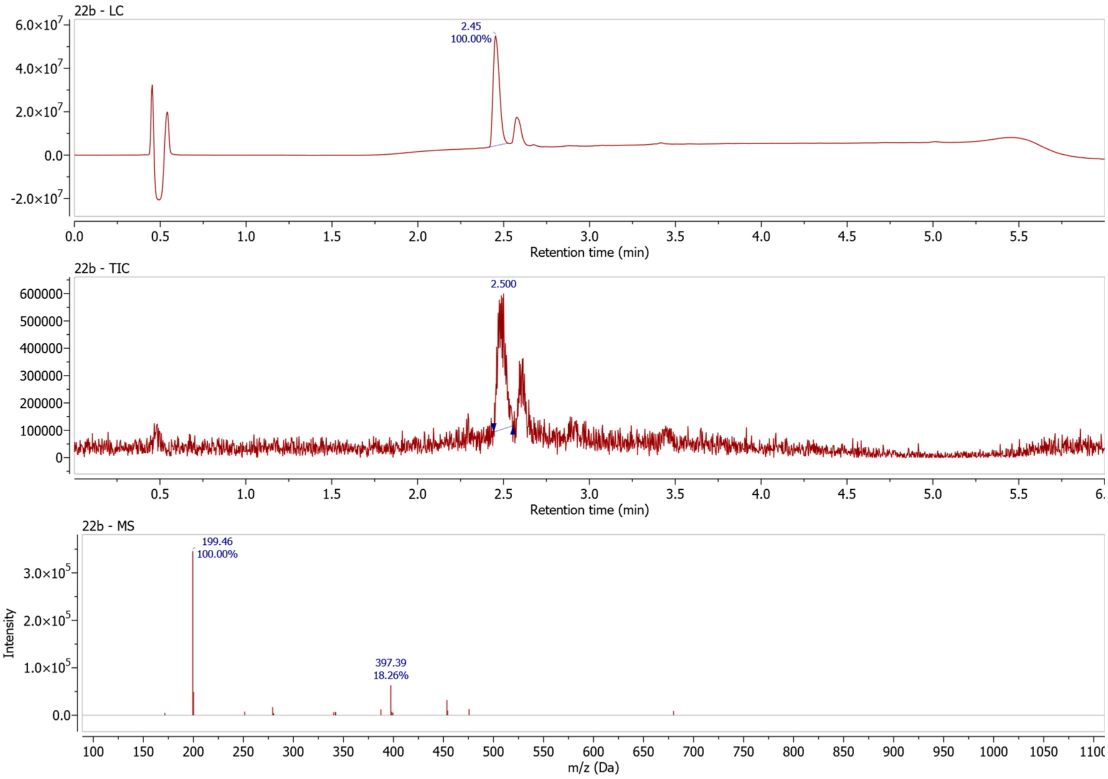

LCMS traces of blank

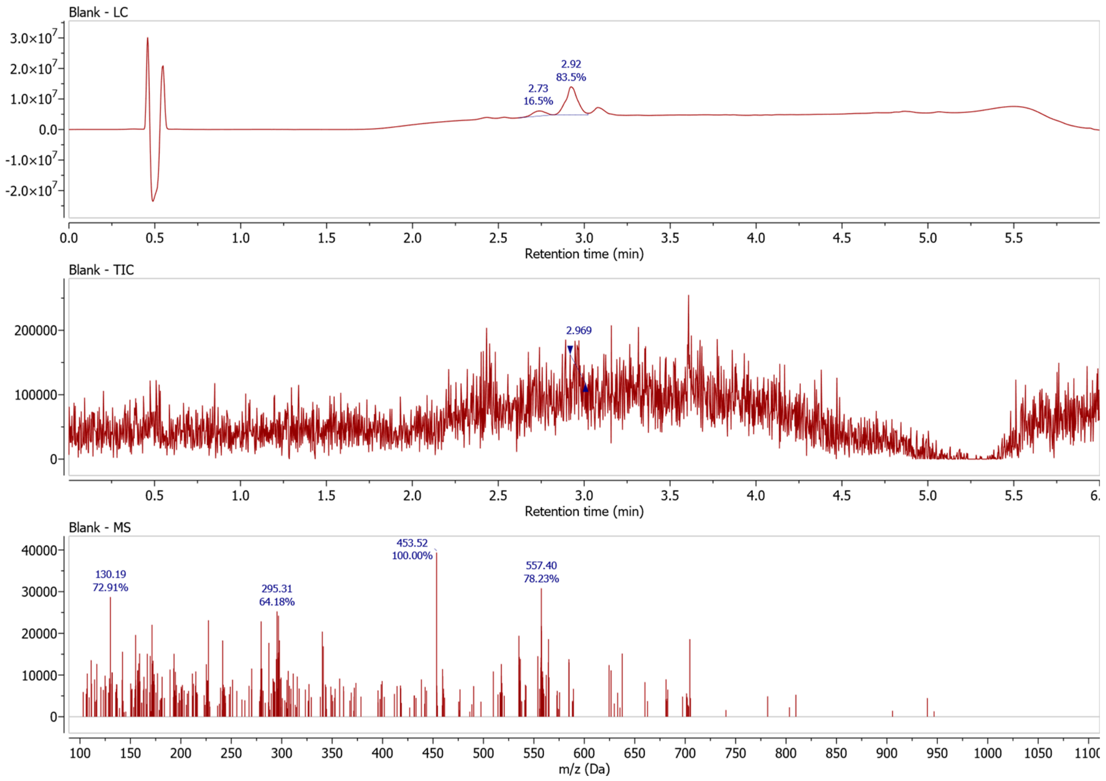

In vitro pharmacology.

Figure S1. Radioligand competition binding concentration-response curves of **13a-b, 14a-b**, and **22a-b** when tested against MOR, KOR, DOR, NPFF1-R and NPFF2-R. (See summary in main text, Table 1)

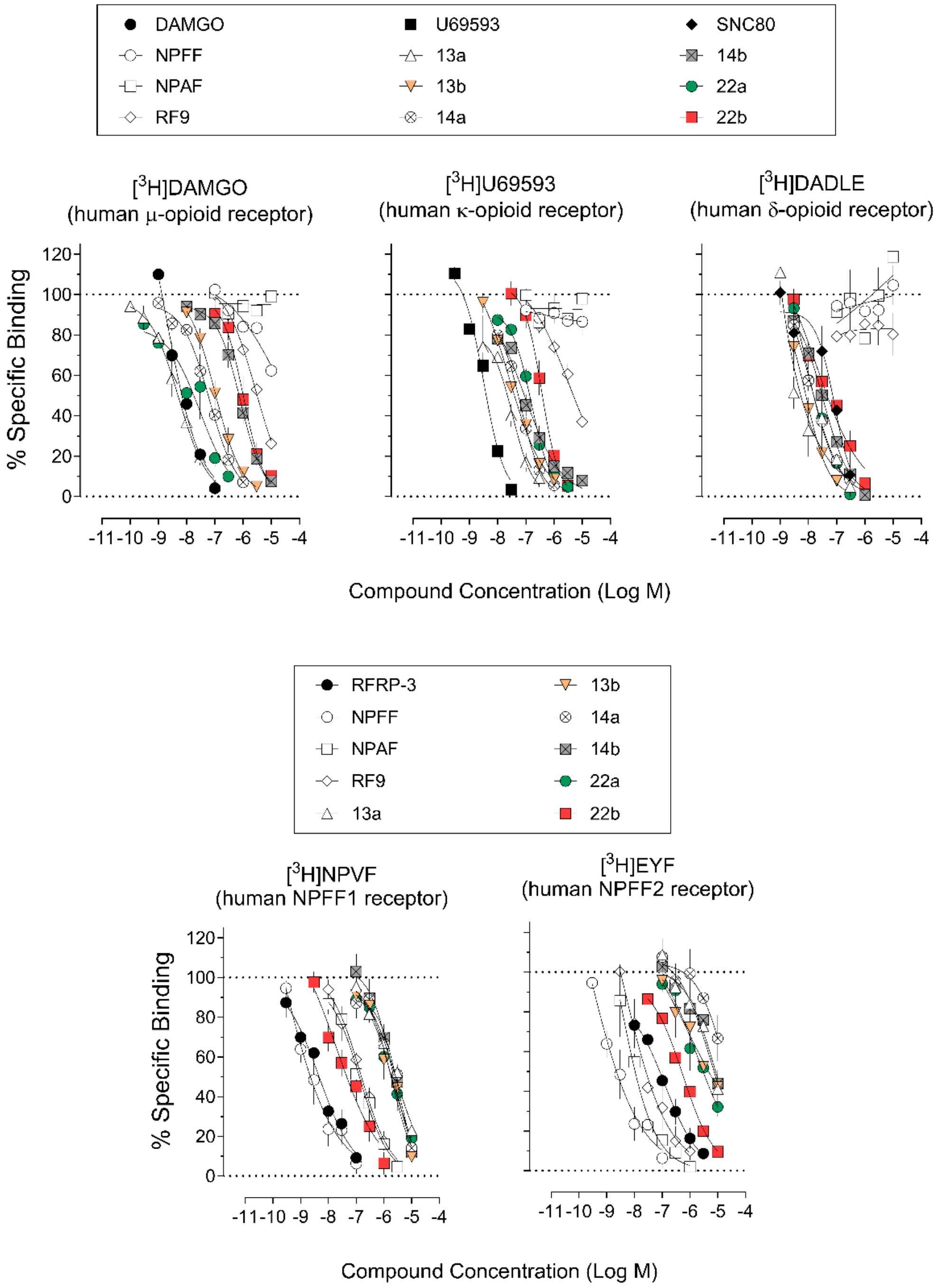

Figure S2. Concentration-response curves assessing functional agonist activities of **13a-b, 14a-b**, and **22a-b** when tested against MOR (left), KOR (middle) and DOR (right) in the [^{35}S]GTPγS assay. (See summary in main text, Table 2).

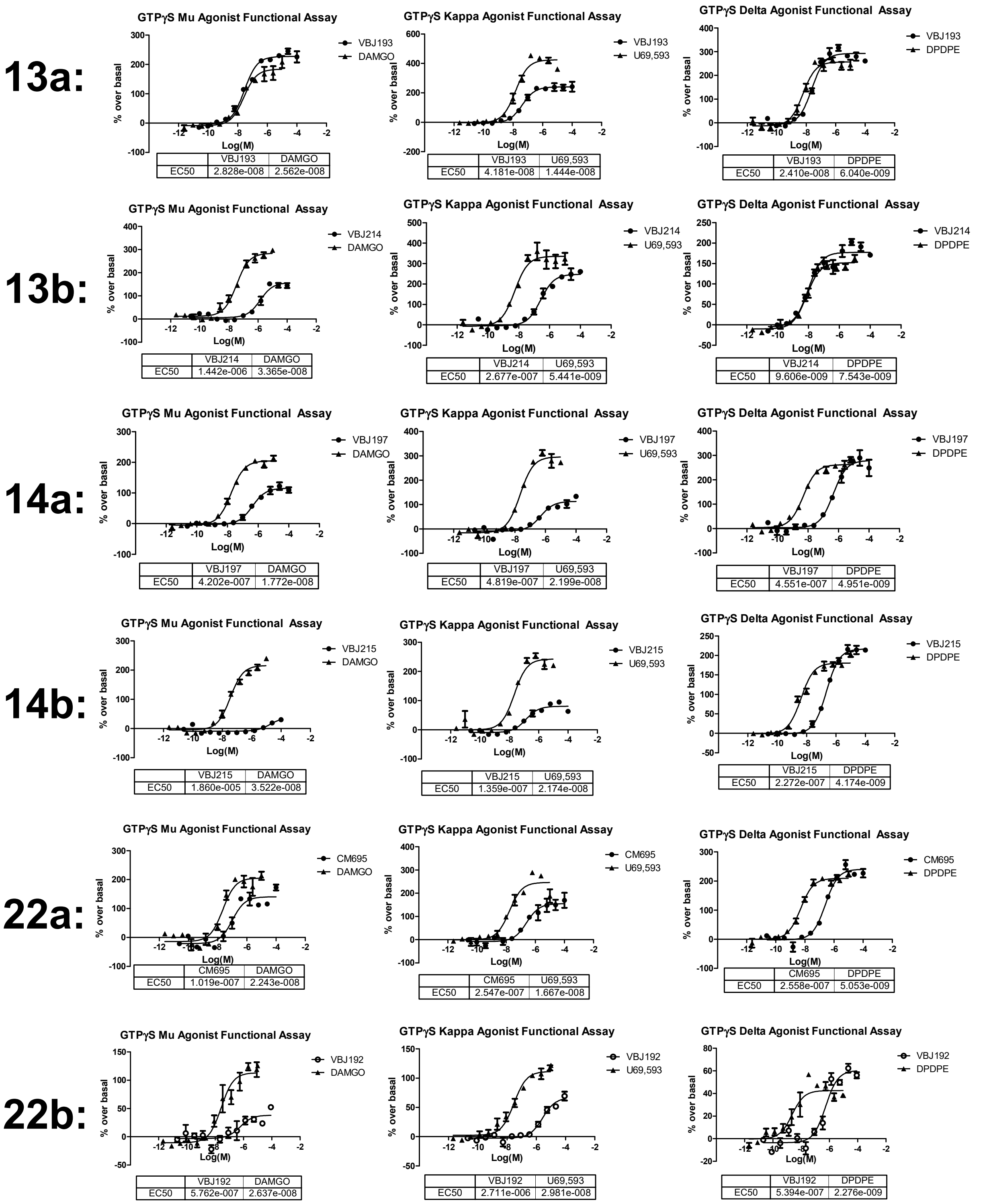

Table S1. Functional Activities of **13a-b, 14a-b**, and **22a-b** when tested alone in the cAMP assay at NPFF1-R and NPFF2-R.*

Functional Activity		
**Cpd.**	**hNPFF1-R** EC_{50} (nM)	**hNPFF2-R** EC_{50} (nM)
**NPFF**†	236 ± 43	2.3 ± 0.5
**NPVF**†	12 ± 2	133 ± 11
**1DMe**†	71 ± 14	2.7 ± 0.5
**RF9**‡	>10^4	NR
**13a**	>10^4	>10^4
**13b**	>10^4	>10^4
**14a**	>10^4	>10^4
**14b**	>10^4	>10^4
**22a**	>10^4	>10^4
**22b**	>10^4	>10^4

*EC_{50} values is the concentration of agonist that inhibits 50% of the intracellular cAMP production induced by 2 μM forskolin in recombinant CHO cells expressing hNPFF1 and hNPFF2 respectively; †Data taken from ref. (46); ‡Data taken from ref. (17); NR = not reported.

Pharmacokinetics.

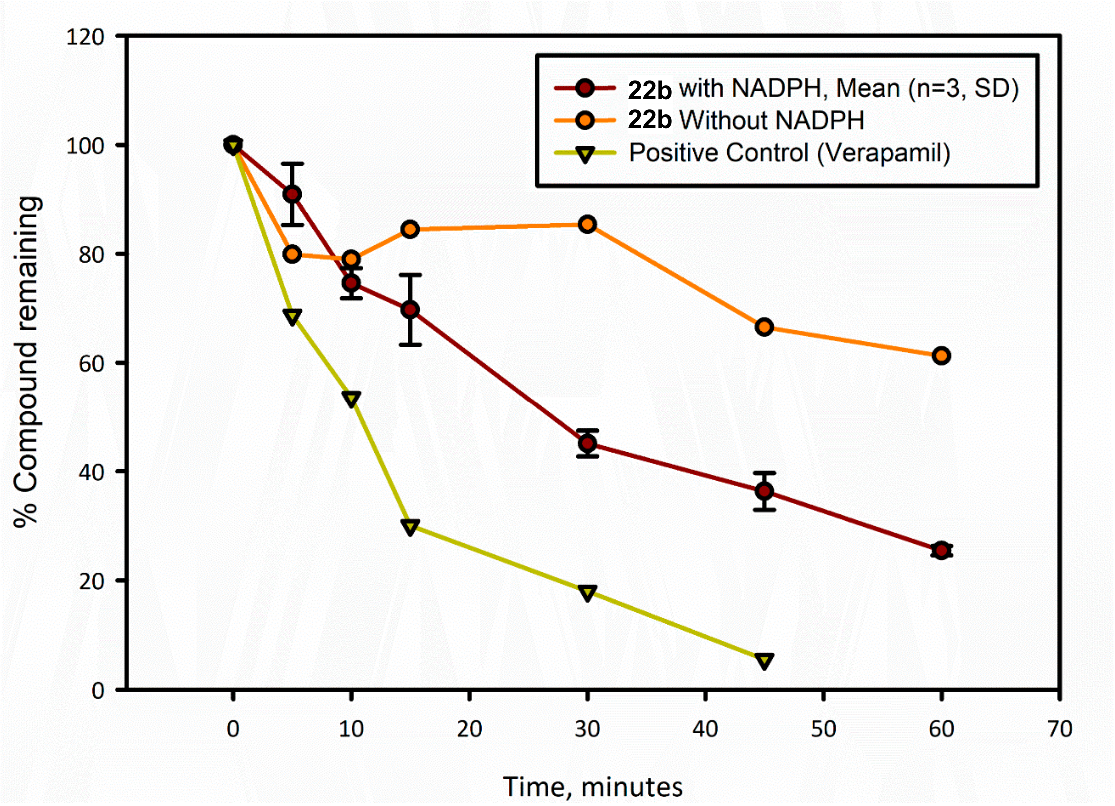

Figure S3. Rat Liver microsomal stability of compound **22b**

$$t_{1/2} = \frac{0.693}{k(min^{-1})}$$

$$CL_{int} = k(min^{-1}) * \frac{[V]incubation\ (mL)}{[P]incubation\ (mg)}$$

$$CL_{int,h} = CL_{int} * \frac{protein\ (mg)}{liver\ (g)} * \frac{liver\ (g)}{body\ weight\ (kg)}$$

Figure S4. Equations used for the calculation of i*n-vitro* half-life ($t_{1/2}$), intrinsic clearance (CL_{int}) and hepatic clearance ($CL_{int,h}$).

Table S2. Gradient elution conditions

Time, min	%A (0.02% TFA in Water)	% B (0.02% TFA in Methanol)
0.0	95	5
1.00	95	5
2.00	5	95
3.00	5	95
3.50	95	5
4.00	95	5

Table S3. MS/MS parameters

Transition	Cone voltage (V)	Collision energy (V)
397.20 > 202.26 (**22b**, quantifier)	20.0	70.0
397.20 > 247.07 (**22b**, qualifier)	20.0	36.0
406.03 > 203.04 (WA475, internal standard)	90.0	28.0

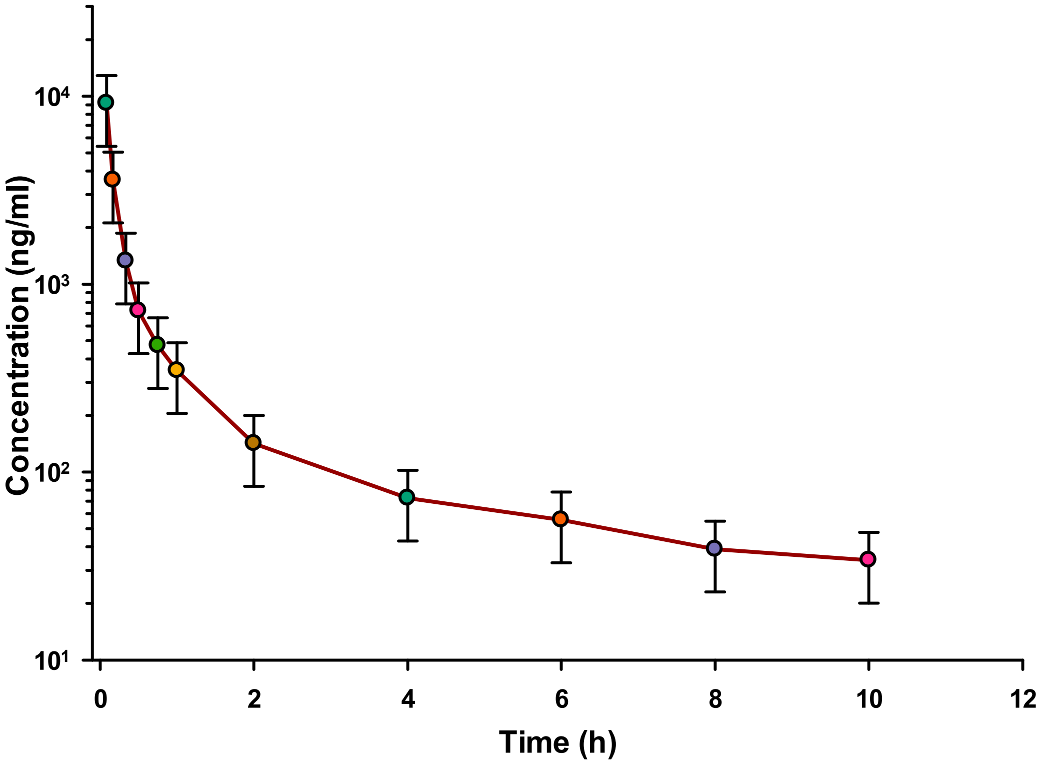

Figure S5. Plasma concentration-time profile of **22b** following intravenous administration (5 mg/kg) in *Sprague Dawley* rats (Mean, SEM).

REFERENCES (from main text, repeated here for convienence)

17. Simonin F*, et al.* (2006) RF9, a potent and selective neuropeptide FF receptor antagonist, prevents opioid-induced tolerance associated with hyperalgesia. *Proc. Natl. Acad. Sci. U. S. A.* 103(2):466-471.

46. Mollereau C*, et al.* (2002) Pharmacological characterization of human NPFF1 and NPFF2 receptors expressed in CHO cells by using NPY Y1 receptor antagonists. *Eur. J. Pharmacol.* 451(3):245-256.
